# Surrogate-assisted multi-objective topology optimization design method for prefabricated large-span Suspen-Dome based on structural-mapping-method and improved-PGSA

**DOI:** 10.1371/journal.pone.0361862

**Published:** 2026-11-02

**Authors:** Yuchen Dai, Ailin Zhang, Liuhaoxiang Wang, Caisen Wang, Guanhua Chen, Yuanyuan Li

**Affiliations:** 1 College of Architecture and Civil Engineering, Beijing University of Technology, Beijing, PR China; 2 Beijing Engineering Research Center of High-rise and Large-Span Pre-stressed Steel Structures, Beijing, PR China; University of Vigo, SPAIN

## Abstract

Existing optimization studies on large-span Suspen-Dome structures have mainly focused on either size optimization of the upper reticulated shell or topology optimization of the lower cable-strut system. Such a separated strategy prevents the integrated optimization of the whole structure, restricts the design space, and limits further improvement in structural performance. This study investigates the integrated optimization of prefabricated large-span Suspen-Dome structures by simultaneously considering the size, geometric configuration, and topology of the upper reticulated shell, together with the topology and cable forces of the lower cable-strut system. To ensure practical applicability, the design variables are classified into topological and size variables. The former define the structural configuration and connectivity, while the latter determine material-related properties. The optimization aims to maximize stiffness and minimize weight, subject to member slenderness, material strength, local stability, and overall structural stability constraints. An Multi-Layer Perceptron (MLP)-based candidate screening model is introduced to accelerate the optimization algorithm and reduce unnecessary ANSYS analyses. A partial full-stress design (PFSD) concept is proposed, in which the reticulated shell serves as a structural safety reserve. The proposed structural mapping method-improved plant growth algorithm (SMM-iPGSA) is validated through two examples. Results show that the optimized structures exhibit improved stability, more uniform stress distribution, and displacements well below the allowable limits.

## 1. Introduction

Suspen-Dome, a spatial structural system that blends rigidity with flexibility and was originally proposed by Mamoru Kawaguchi, comprise an upper single-layer reticulated shell coupled with a lower cable-strut system. Owing to their elegant form, rational load paths, cost-effectiveness, and suitability for long spans, Suspen-Dome has been widely adopted in large-scale facilities such as stadiums, exhibition centers, and airports [1–3].

The Suspen-Dome enhances the structural performance of the upper single-layer reticulated shell through its lower prestressed cable-strut system [4], thereby improving the overall load-carrying capacity. Optimization is one of the key issues for the Suspen-Dome. Current optimization studies typically focus either on prestress optimization of the Suspen-Dome-including topology optimization of the lower cable-strut system-or on sizing and geometric optimization of the upper reticulated shell. Zhang et al. [5] employed a particle swarm optimization (PSO) scheme in a second-stage optimization and proposed an effective prestress optimization method; however, the design variables were limited to cable prestress and cable cross-sectional areas of cable-strut system. Kitipornchai et al. [6] proposed a convenient cable-force optimization approach by exploiting the central symmetry of the Suspen-Dome. Pan et al. [7] adopted an Improved-PGSA with the objective of minimizing horizontal stress to optimize cable prestress. Kaveh and Rezaei [8] performed topology optimization of the Suspen-Dome using the Enhanced Colliding Bodies Optimization (ECBO) algorithm. Jiang et al. [9] optimized cable prestress with a hybrid PGSA-PSO method, demonstrating its feasibility. Shen et al. [10] proposed an ASDS, enabling the struts of the Suspen-Dome to adapt their configuration and stiffness to external environmental conditions.

Topology optimization, by employing mathematical and computational techniques, unlocks material performance and expands design creativity, and thus plays a pivotal role in structural optimization. Gao [11] et al. proposed an enhanced proportional topology optimization (EPTO) method by improving the proportional topology optimization algorithm and combining it with level set-based post-processing, which effectively reduced gray regions and generated smoother topological boundaries suitable for engineering applications. Wang [12] et al. developed a parameterized level set method (PLSM) for thermo-mechanical coupled topology optimization, where compactly-supported radial basis functions and an approximate re-initialization scheme were introduced to improve computational efficiency, numerical stability, and convergence. Cui [13] et al. combined an asymptotic concentration approach with isogeometric analysis (IGA) under the SIMP framework, which gradually eliminated intermediate-density regions and improved the accuracy of topology optimization results. Although these methods have achieved remarkable success in continuum topology optimization, they are primarily developed for continuum structures based on material distribution optimization. For spatial reticulated structures such as Suspen-Domes, topology optimization mainly involves discrete design variables, including member connectivity, cross-sectional properties, and prestressing, making these continuum-based methods difficult to apply directly. Xia et al. [14] proposed an integrated approach that simultaneously optimizes the shape and topology of free-form shells, which simplifies the coupled optimization problem and reduces computational complexity; however, size effects were not considered. Shi et al. [15] developed a shape-optimization method based on an improved GSL&PS-PGSA that effectively explores rational structural forms, enhances mechanical performance, and reduces sensitivity to geometric imperfections, but it does not address topology optimization of reticulated shells. Cai et al. [16] combined isogeometric analysis (IGA), the adaptive bubble method (ABM), and the finite cell method (FCM) to leverage their respective strengths in shell optimization, enabling concurrent shape and topology optimization of shell structures. Ruben Ansola et al. [17] proposed an automated framework that couples shape and topology optimization with the objective of maximizing shell stiffness; however, it does not accommodate alternative objective and constraint functions or multiple load cases. Thuan Ho-Nguyen-Tan [18] combined a gradient-based shape optimization method with a level-set-based topology optimization method to minimize the compliance of shell structures subject to a volume constraint. Saaranya Kumar Dasari et al. [19] employed a two-stage optimization strategy for shell topology optimization, which substantially reduced material usage and lowered the manufacturing- and transportation-related carbon footprint. Zhao et al. [20] conducted topology optimization of space truss structures using a numerical inverse hanging method in combination with the fully stressed design (FSD) principle, thereby enabling the automatic determination of member cross-sectional areas from internal forces and allowable design strength. Chen et al. [21] designed steel joints for spatial grid structures on the Grasshopper platform, achieving fully parametric topology-optimized configurations characterized by high design flexibility, strong integration, and good portability. Coen Bakker [22] proposed a method that simultaneously optimizes the topology of stiffening ribs and their layout on a base shell or plate, improving quality control and facilitating large-scale manufacturing.

Multi-objective optimization (MOO) has been extensively investigated in operations research and structural optimization. Branke [23] argued that MOO is essentially an interactive process, in which additional preference information is incorporated to guide the search toward satisfactory compromise solutions. This process is commonly referred to as multi-criteria decision analysis (MCDA) [24]. MCDA offers flexibility in decision making, enables holistic trade-off assessment, and supports efficient exploration of the solution space [25–27]. Ma [28] proposed a multi-objective prestress optimization approach for cable-strut structures that considers structural stability, economy, and feasibility, achieving optimal prestress schemes; however, the computational burden is relatively high. Dai et al. [29] enhanced convergence while preserving solution diversity by decomposing the objective space and applying a crowding-distance scheme. By finely managing the objective space, this approach addresses limitations of traditional algorithms in high-dimensional settings. Zhu et al. [30] performed multi-objective optimization of welded hollow-sphere joints with static stiffness and dynamic (natural) frequencies as objectives; the optimized joints exhibited greater deformation resistance and higher low-order frequencies, but the interaction between the joint and the overall structural system was not considered. Pan et al. [31] applied a multi-objective optimization framework to the early design of multifunctional sports venues, integrating interdisciplinary numerical assessments into design decision-making and thereby broadening and improving the efficiency of design-space exploration.

In multi-objective optimization, two objective functions are typically considered. Ohsaki et al. addressed tensegrity grid structures by maximizing the smallest eigenvalue of the tangent stiffness matrix while simultaneously minimizing compliance [32]; in some cases the objectives are modified to maximizing the smallest eigenvalue of the tangent stiffness matrix and minimizing prestress deviation [33]. Adam et al, by contrast, considered minimizing member stresses and maximizing overall structural stiffness [34].

In summary, although existing structural optimization methods are capable of addressing large-scale design variables and multi-objective optimization problems, and have achieved good application performance in several similar structural systems, the design problem of long-span Suspen-Dome structures still exhibits clear particularity. Specifically, a Suspen-Dome consists of an upper reticulated shell and a lower cable-strut system, and there exists a significant coupling relationship between them in terms of mechanical behavior, stiffness, and stability. Therefore, it cannot be simply regarded as two independent subsystems for separate optimization. Meanwhile, long-span Suspen-Dome structures usually possess a large number of members, complex topological forms, and significant coupling between geometric parameters and mechanical parameters, and their optimization variables often simultaneously involve multiple types of variables, including member cross-sectional sizes, cable prestress, structural geometric shape, and structural topology, which causes the optimization problem to exhibit the characteristics of high dimensionality, strong nonlinearity, and multi-constraint coupling. Particularly under long-span conditions, the control of overall structural stability and stiffness often becomes an important difficulty that distinguishes such optimization from that of general spatial structures.

To address the above issues, this study develops an SMM-iPGSA-based multi-objective optimization method for the coupled design of long-span Suspen-Dome structures. The originality of the present study does not lie in re-proposing the structural mapping method or iPGSA themselves, but in extending them to a unified discrete optimization problem in which the topology, geometry, and member sections of the upper reticulated shell are optimized together with the topology, member sizes, and cable prestress of the lower cable–strut system. This treatment differs from conventional sequential or subsystem-oriented optimization because the design variables of the two mechanically coupled subsystems are allowed to interact during the search process under the same structural constraints.

The methodological significance of this extension lies in establishing a unified design-variable representation and optimization procedure for the topology–size–prestress coupled problem of Suspen-Dome structures. Structural mass and the aggregate axial stiffness index are adopted as the optimization objectives, while member strength, slenderness, local stability, overall stability, and other structural requirements are incorporated into the same optimization model. The SMM provides the geometric and topological mapping required when the structural configuration changes, whereas iPGSA is used to search the resulting mixed discrete design space. An MLP-based classifier is employed only as an auxiliary candidate-screening tool to reduce unnecessary finite-element analyses, and the PFSD strategy is used as a structural design criterion to coordinate material utilization in the lower cable–strut system with the stiffness and stability reserve of the upper reticulated shell; neither is claimed as an independent general-purpose optimization theory.

The method is evaluated at three levels. First, the search performance of iPGSA is examined using benchmark functions and a reticulated-shell optimization example with comparison algorithms. Second, the complete optimization procedure is applied to a 100 m-span Kiewitt–Lamella Suspen-Dome and a 110 m-span rib-ring Suspen-Dome to examine its applicability to different structural configurations. Third, additional validation is conducted through MLP cross-validation, load-sensitivity analysis, stochastic robustness tests, and a controlled comparison between sequential and integrated optimization strategies. The latter comparison is specifically introduced to quantify the effect of allowing the upper and lower subsystems to participate in the same optimization process, rather than attributing the resulting improvement solely to the underlying search algorithm.

## 2. Structural mapping method and improved PGSA

### 2.1. Structural mapping method

The Structural Mapping Method (SMM) is a force-density-based design procedure for prefabricated, large-span Suspen-Dome with single-layer reticulated shells. It comprises two stages: (i) constructing a two-dimensional planar pattern that unambiguously specifies nodal planar coordinates and member connectivity; and (ii) performing form-finding using an improved force-density method. In combination, these stages enable rapid, convenient, and flexible synthesis of large-span Suspen-Dome reticulated shells while effectively mitigating the adverse effects of geometric and fabrication deviations.

**Method details:**

(1) First, provide the planar layout algorithm with the target circumferential segmentation angles, ring spacing, and the prescribed span of the reticulated shell. The form-finding algorithm then outputs the nodes, their coordinates, and a connectivity matrix. Note that this connectivity matrix is not a topology (adjacency) matrix; rather, it is an **n** × 2 edge list that records node–node incidences, which substantially reduces the computational burden compared with the **n** × **n** adjacency representation used in conventional approaches.
(2) Number the nodes of the reticulated shell; each member is defined by a pair of node indices (i, j) in the connectivity matrix, where the first row corresponds to the first member, and so on. Members are ordered as follows: first-ring radials, first-ring circumferentials, then subsequent rings in sequence. The outermost (last) ring is designated as fixed boundary nodes. Such indexing facilitates subsequent computation and data handling; with these identifiers, nodes and members can be processed efficiently within the improved force-density method, enhancing both computational efficiency and accuracy. Details are shown in Fig 1.

**Fig 1:**
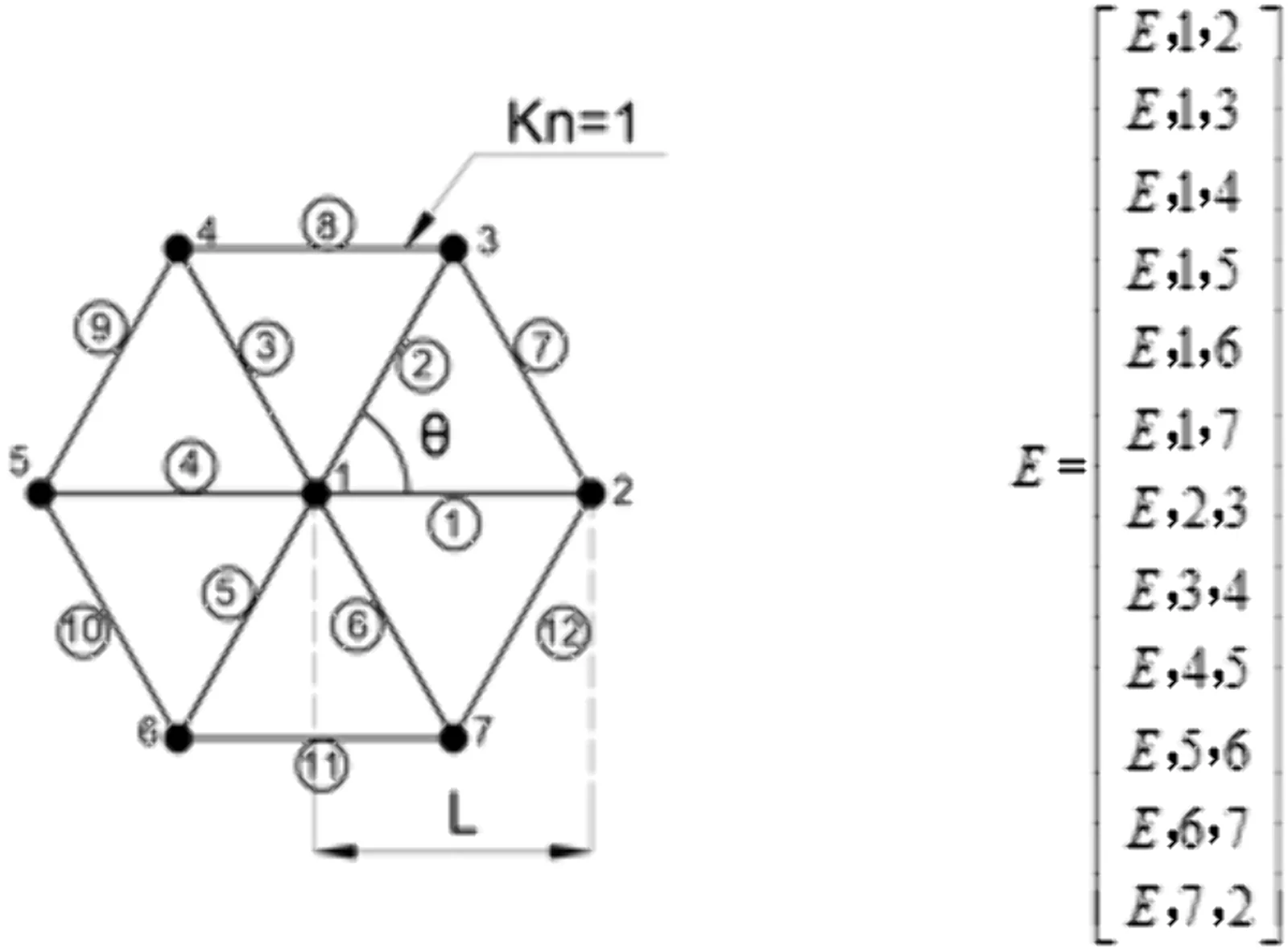
Connectivity matrix.

Fig 1 illustrates the connectivity matrix of the planar structure, which is expressed in the form of E, I_1_, and I_2_. Here, E denotes the element, namely, a structural member, whereas I_1_ and I_2_ denote the start and end nodes of the element, respectively. This representation has two advantages. First, it can be directly interfaced with commercial computational software such as ANSYS and mathematical computing software such as MATLAB. Second, it avoids the considerable computational cost associated with the n × n matrix used in the original method by reducing the connectivity representation to an n × 2 matrix, thereby facilitating subsequent operations.

(3) Input the node indices, nodal coordinates, and member connectivity into the improved force-density algorithm. In the connectivity matrix, the first column denotes the start node and the second denotes the end node, with each row corresponding to a single member (element) ID. However, a fixed orientation is not required-the order of the two node indices is immaterial—which enhances the generality of the algorithm. Fig 2 has shown that how the member-connectivity matrix is constructed, we now consider a simple single-layer reticulated shell.

**Fig 2:**
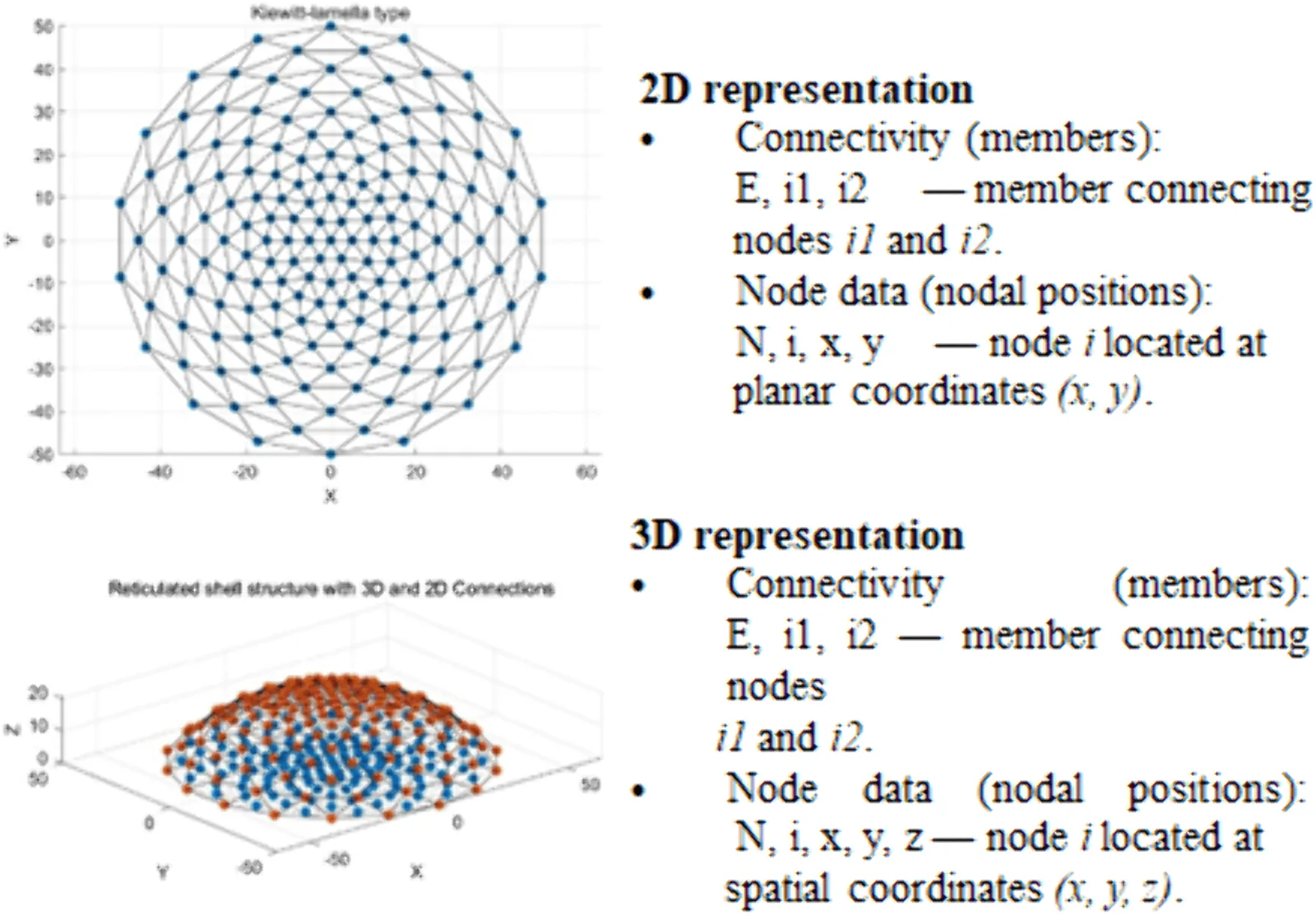
Schematic of the Structural Mapping Method (SMM).

From the perspective of the method mechanism, the applicable object of the proposed method is not limited to a specific structural type, but to regular discrete spatial structures with clear topological relationships. For such structures, as long as the structural system can be abstracted as a spatial connection network composed of nodes and linear members, and a unified geometric parameter system, topological relationship, and mechanical analysis model can be further established, the proposed method remains valid. In other words, the differences among different reticulated shells or long-span spatial structures are mainly reflected in the variations in node arrangement, member connection pattern, and parameter value range, rather than in changes to the basic data representation form and solution procedure on which the proposed method relies. Therefore, the proposed method theoretically has potential applicability for extension to other regular reticulated shell structures and similar long-span spatial structures.

### 2.2. Improved PGSA

The Plant Growth Simulation Algorithm (PGSA), first proposed in 2005 by Li et al. [35], is a novel intelligent optimization method rooted in the theory of morphogen concentration. Inspired by plant phototropism, the feasible domain of an optimization problem is regarded as the plant’s growth environment, while variations of the objective function define the growth signal, i.e., the morphogen concentration. In this way, PGSA simulates the growth dynamics of plants that “rapidly” develop toward the global optimum.

To further enhance the algorithm’s ability to locate the global optimum within the solution space, we introduce a random parallel search mechanism together with a best-value elimination mechanism. The specific improvements are as follows:

(1) **Random parallel search mechanism.**

After the original hybrid step-length search, construct a candidate matrix of size ***m×(5 × n)***, where m is the number of design variables and n is the number of feasible candidate sets. Introduce a random index matrix ***I∈{1,…,5n}***^***m×q***^, where **q** is the prescribed number of random resamplings; each entry ***I***_***ij***_ indexes a column of the candidate matrix. In parallel, generate a random objective-selection matrix ***R∈***$R^{m\times q}$(e.g., drawing either discrete objective labels or random weights over the objectives). The pair ***(I, R)*** enables randomized, parallel re-sampling and evaluation of candidates, which helps the search escape local optima and increases the efficiency of approaching the global optimum.

(2) **Best-value pruning mechanism.**

At each iteration, discard all candidate variable sets whose objective value exceeds the previous iteration’s best (for minimization problems; analogously, discard those below the best for maximization). Coupled with the random parallel search, this pruning rapidly shrinks the effective search region, improving search efficiency and accelerating convergence.

### 2.3. Optimization performance of the improved PGSA

The optimization performance is validated using the numerical examples presented in [36]. Objective function 1 is a unimodal function, whereas Objective function 2 is a multimodal function.

Example 1:


$$\begin{array}{c} \text{min}f\left(\overset{―}{x}\right)={\left(x_{\text{1}}+\text{3}\right)}^{\text{2}}+{\left(x_{\text{2}}-\text{2}\right)}^{\text{2}} \end{array}$$

$$\begin{array}{c} \text{s}.\text{t}.\;-\text{20}\leq x_{i}\leq \text{2}\text{0}\;i=\text{1},\text{2} \end{array}$$ (1)

$$\begin{array}{c} \text{min}f\left(\overset{―}{x}\right)=\left({\left(x_{\text{1}}^{\text{2}}-\text{3}\right)}^{\text{2}}-x_{\text{1}}\right)+\left({\left(x_{\text{2}}^{\text{2}}-\text{3}\right)}^{\text{2}}-x_{\text{2}}\right) \end{array}$$

$$\begin{array}{c} \text{s}.\text{t}.\;-\text{2}.\text{5}\leq x_{i}\leq \text{2}\;i=\text{1},\text{2} \end{array}$$ (2)

where $x_{i}$ denotes the optimization variable. For the unimodal function, It can be seen from Fig 3(a) its feasible domain is [−20,20], while the feasible domain of the multimodal function is [−2.5,2], and the required accuracy for both is 0.1. It can be seen from Fig 3(b) that Growth Space Limited & Parallel Search-based Plant Growth Simulation Algorithm (GSI&PS-PGSA) reaches the vicinity of the optimal value 0 after 13 growth steps and attains the theoretical optimal value 0 after 110 growth steps, whereas PGSA only obtains the current optimal value of 176.57 at this stage. In contrast, iPGSA requires only 6 growth steps to reach the optimal value 0, reducing the number of growth steps by 94.5%. For the multimodal function, $\overset{―}{x}=$ (1.8,1.8) is the theoretical global optimal solution, and the corresponding theoretical optimal value is 3.4848. In the original study, four points, $x_{\text{1}}=(-\text{0}.\text{5},\text{0}.\text{3}),\;x_{\text{2}}=(\text{0}.\text{5},\text{0}.\text{3}),x_{\text{3}}=(-\text{0}.\text{5},-\text{0}.\text{3}),x_{\text{4}}=(\text{0}.\text{5},-\text{0}.\text{3})$, were selected for optimization. When the set of growth points was 50, GSI&PS-PGSA reached the global optimal solution from the four initial points with probabilities of 98%, 75%, 80%, and 56%, respectively, after an average of 52, 75, 39, and 54 search steps, whereas iPGSA could reach the global optimal solution from all four initial points with a probability of 100% after an average of 22 search steps, which is only about 1/3–1/4 of the number of search steps required by the original algorithm. This verifies the stability and efficiency of the proposed algorithm. Table 1 presents the statistical results for Example 1.

**Table 1:** Statistical results of repeated independent runs of iPGSA and comparison algorithms on benchmark functions.

Test function	Algorithm	Success rate (%)	Average number of iterations
Unimodal function	PGSA	100	2000+
	GSI&PS-PGSA	100	110
	iPGSA	100	6
Multimodal function	PGSA	–	–
	GSI&PS-PGSA	77	55
	iPGSA	100	22

**Fig 3:**
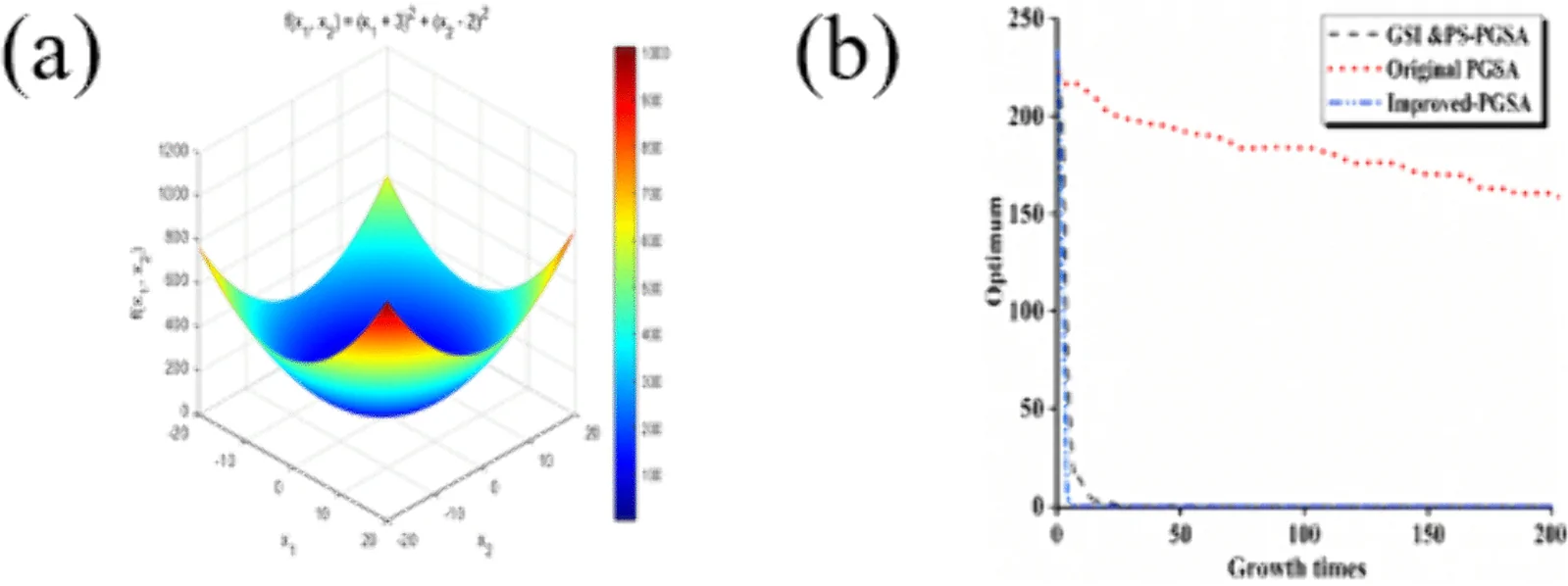
Unimodal function. (a) Unimodal function. (b) Improvement effect.

Example 2:

In this example, the single-layer reticulated shell reported in Ref [37]. is selected to investigate and compare the advantages and disadvantages of the iPGSA method with other traditional heuristic algorithms. As shown in Fig 4, the structure has a span of 70 m, a rise of 20 m, and a rise-to-span ratio of 1/3.5, with a total of 10 rings, 930 members, and 331 nodes. The outermost ring nodes are arranged as fixed hinged supports, and welded hollow spheres are adopted at the joints. Q235 steel tubes are used, with a design strength of f=215 N/mm^2^, an elastic modulus of E= 2.06 ×10^5^ N/ mm^2^, a Poisson’s ratio of ν = 0.3, and a density of ρ  =7.85 ×10^3 kg^/m^3^.

**Fig 4:**
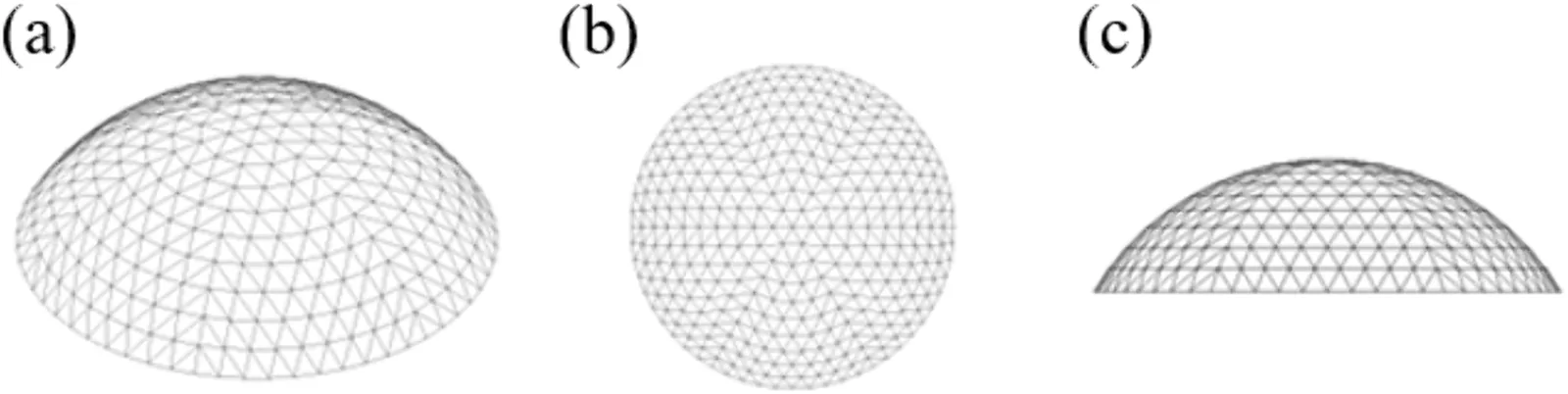
Model of the single-layer spherical reticulated shell structure of type K6. a) Axonometric drawing. b) Floor plan. c) Elevation view.

In the original study, seismic action was not considered, and only the sectional optimization analysis of the structure under static load conditions was carried out. From the inner ring to the outer ring, every two rings of circumferential members were taken as one group, and every two rings of radial members were taken as one group, resulting in a total of ten groups of variables. The remaining load conditions and constraint conditions were all consistent with those in the original paper. To ensure consistency with the settings adopted in the original study, the maximum number of iterations in ANSYS was set to 50 for all algorithms, while the population size was set to 10. The population size is referred to as SwarmSize in PSO and as the number of growth points in iPGSA. The convergence time and iteration numbers of different heuristic algorithms are shown in the Table 2:

**Table 2:** Comparison of algorithm convergence behavior.

	iPGSA	GA [37]	ANGA [37]	PSO
Convergence time	12min	47min	49min	36min
Number of iterations	18	25	27	22

### 2.4. Summary

Compared with the original PGSA, the proposed method enhances the global exploration capability through a random parallel search mechanism, thereby improving the possibility of escaping from local optima; through the optimal-value elimination mechanism, invalid searches are reduced, and the convergence efficiency is improved. Meanwhile, after being combined with the Structural Mapping Method, the algorithm can more effectively deal with the coordinated optimization of multiple types of variables in Suspen-Dome structures, including topology, geometry, and cross sections. Therefore, the proposed method shows improvement over the original PGSA in terms of global search capability, convergence speed, and stability.

## 3. Topology optimization workflow for prefabricated long-span Suspen-Dome based on the structural mapping method and improved-PGSA

### 3.1. Formulation of the mathematical model

#### 3.1.1. Objective functions.

(1) **Structural weight**

Large-span spatial structures, due to their extensive span and numerous members, are highly sensitive to their self-weight. Excessive structural self-weight reduces material utilization efficiency, limits the potential span capacity, and decreases the contribution ratio of prestressed cables, thereby undermining the inherent characteristic of the cable-strut dome that combines “rigidity and flexibility.” Therefore, minimizing structural weight is one of the optimization objectives to balance material usage, safety, and cost. The objective function is expressed as:


$$\begin{array}{c} M(x)=\sum_{e}^{N_{e}}\rho \cdot A(x)\cdot L(x),f_{\text{1}}(x)=\text{min }\left(M(x)\right) \end{array}$$ (3)

M(x) represents the structural weight.

(2) Aggregate axial stiffness index

The Suspen-Dome consists primarily of upper reticulated-shell members, struts, tie rods, and prestressed cables, all of which participate in structural load transfer mainly through axial forces. For candidate structures with different topological configurations and cross-sectional arrangements, the overall level of member axial stiffness can be used to characterize their relative resistance to deformation. Therefore, the sum of the member axial stiffnesses, EA/L, is adopted in this study to establish an aggregate axial stiffness index:


$$\begin{array}{c} K(x)=\sum_{e}^{N_{e}}\frac{E\cdot A(x)}{L(x)},f_{\text{2}}(x)=\text{max }\left(K(x)\right) \end{array}$$ (4)

K(x) represents aggregate axial stiffness index.

#### 3.1.2. Constraint conditions.

In the topology optimization model, solutions that fail to satisfy the imposed constraints are assigned limiting conditions, thereby ensuring that the solution space remains within the feasible domain. This treatment improves both the practicality and the validity of the optimization results.

(1) **Displacement Constraint**

In structural topology optimization design, the limitation of displacement extrema serves as a critical constraint to ensure both the safety and functionality of the structure. By prescribing the maximum allowable displacement within the design domain, the required performance can be guaranteed. According to the *Technical Specification for Space Grid Structures*(JGJ 7–2010), the corresponding limit values are defined as follows:


$$\begin{array}{c} \delta \leq \frac{L}{\text{4}\text{0}\text{0}} \end{array}$$ (5)

where δ denotes the maximum deflection.

(2) **Allowable Slenderness Ratio**

The slenderness ratio governs the stability performance of structural members, and its expression is given as:


$$\begin{array}{c} \lambda =\frac{l_{\text{0}}}{i}\leq [\lambda ] \end{array}$$ (6)

where λ is the slenderness ratio, $l_{\text{0}}\;$is the effective length of the member, and *i* is the radius of gyration.

(3) **Member Section Strength**

The internal force of a member shall not exceed the material strength. The strength constraint can be expressed as:


$$\begin{array}{c} \frac{N}{A_{n}}+\frac{\sqrt{M_{x}^{\text{2}}+M_{y}^{\text{2}}}}{\gamma_{m}W_{n}}\leq f \end{array}$$ (7)

(4) **Stability of Compression–Bending Members**

In large-span spatial structures, members are often subjected to combined loading conditions, and their stability constitutes an essential constraint. The corresponding expression is given as:


$$\begin{array}{c} \frac{N}{\varphi Af}+\frac{\beta M}{\gamma_{m}W(\text{1}-\text{0}.\text{8}\frac{N}{N_{Ex}^{\prime }})f}\leq \text{1}.\text{0} \end{array}$$ (8)

(5) **Structural Safety Factor**

The overall stability of the structure governs its failure mode and serves as a critical criterion for addressing variations in load values, material imperfections, and construction errors. The corresponding expression is given as:

Eigenvalue Buckling Safety Factor:


$$\begin{array}{c} \lambda_{cr}\geq \text{4}.\text{2} \end{array}$$ (9)

Nonlinear Buckling Safety Factor:


$$\begin{array}{c} \lambda_{taeget}\geq \text{2}.\text{0} \end{array}$$ (10)

In Eqs. (5)–(10), the meanings of the parameters are listed in Table 3.

**Table 3:** Constraint variables.

Parameter name	Parameter symbol
Maximum vertical displacement limit	δ
Span	L
Slenderness ratio	λ
Effective length of the member	$l_{\text{0}}$
Radius of gyration	i
Design bending moments about the 𝑥 and 𝑦 axes at the same section; Bending moment under stability	$M_{x},M_{y},M$
Section plastic development factor	$\gamma_{m}$
Net section modulus, Section modulus	$W_{n},W$
Net area, Area	$A_{n},A$
Design strength	f
Stability coefficient	φ
In-plane Euler force, Axial force	$N_{Ex}^{\prime },N$
Equivalent Bending Moment Coefficient	β
Eigenvalue Buckling Safety Factor	$\lambda_{cr}$
Nonlinear Buckling Safety Factor	$\lambda_{target}$

#### 3.1.3. Design variables.

(1) **Topology Optimization Variables**

In this study, the parameters controlling the structural configuration are treated as topology optimization variables. These variables include: the number of circumferential rings ***Kn***, the number of radial members ***Nx***, the cable force ***N***, and the interval number of the cable-strut system ***interval***.

(2) **Size Optimization Variables**

Variables that modify the cross-sectional dimensions of individual members are referred to as size optimization variables. These primarily include the cross-section index ***x***, the cross-sectional area of the struts and ties ***a***, and the lengths of the struts and ties ***l***_***push***_ and ***l***_***pull.***_

#### 3.1.4. Complete mathematical model.

Based on the objective functions, design variables, and constraint conditions described above, the normalized topology–size–prestress collaborative optimization model for the Suspen-Dome can be expressed as follows:


$$\begin{array}{c} minF(x)=[\begin{array}{c} f_{\text{1}}(x)\\ f_{\text{2}}(x) \end{array}]=[\begin{array}{c} \frac{\text{M}(x)}{M_{\text{0}}}\\ \frac{K_{\text{0}}}{\text{K}(x)} \end{array}] \end{array}$$ (11)

$$\begin{array}{c} s.t.\;g(x)\leq \text{0};\;\left\{ \begin{array}{c} @rg_{\text{1}}(x)=\delta_{max}-\delta_{allow}\leq \text{0}\\ g_{\text{2}}(x)=\sigma_{i,max}-\sigma_{i,allow}\leq \text{0}\\ g_{\text{3}}(x)=\lambda_{i,max}-\lambda_{i,allow}\leq \text{0}\\ g_{\text{4}}(x)=\lambda_{cr,target}-\lambda_{stable_{a}llow}\leq \text{0}\\ g_{\text{5}}(x)=\lambda_{eig,allow}-\lambda_{eig}\leq \text{0}\\ g_{\text{6}}(x)=\lambda_{nl,allow}-\lambda_{nl}\leq \text{0}\\ g_{\text{7}}(x)=-min[T(x)]\leq \text{0} \end{array}\right. \end{array}$$ (12)

$$\begin{array}{c} x=\left[x_{topo,\;}x_{geo\;},x_{sec},x_{pre}\right];\left\{ \begin{array}{c} @cx_{topo}={\left[Kn,Nx,interval\_Kn,interval\_Nx,q_{input}\ldots \right]}^{T}\\ x_{sec}={\left[x_{s\text{1}},x_{s\text{2}}\ldots x_{sn},x_{st\text{1}},x_{st\text{2}}\ldots x_{stn}\right]}^{T}\\ \;\;\;\;\;\;\;x_{geo}={\left[f,l_{pull\text{1}},l_{pull\text{2}}\ldots l_{pulln},l_{push\text{1}},l_{push\text{2}}\ldots l_{pushn}\right]}^{T}\\ x_{pre}={\left[N_{\text{1}},N_{\text{2}}\ldots N_{n}\right]}^{T} \end{array}\right. \end{array}$$ (13)

#### 3.1.5. Suspen-Dome structural model.

The basic topology optimization model of the Suspen-Dome structure is shown in the Fig 5, in which *Kn* = 8, *Nx* = 12, ${interval}_{Kn}$=1*,*$\;{interval}_{Nx}$=1. The dimensional parameters are not further presented in Fig 5.

**Fig 5:**
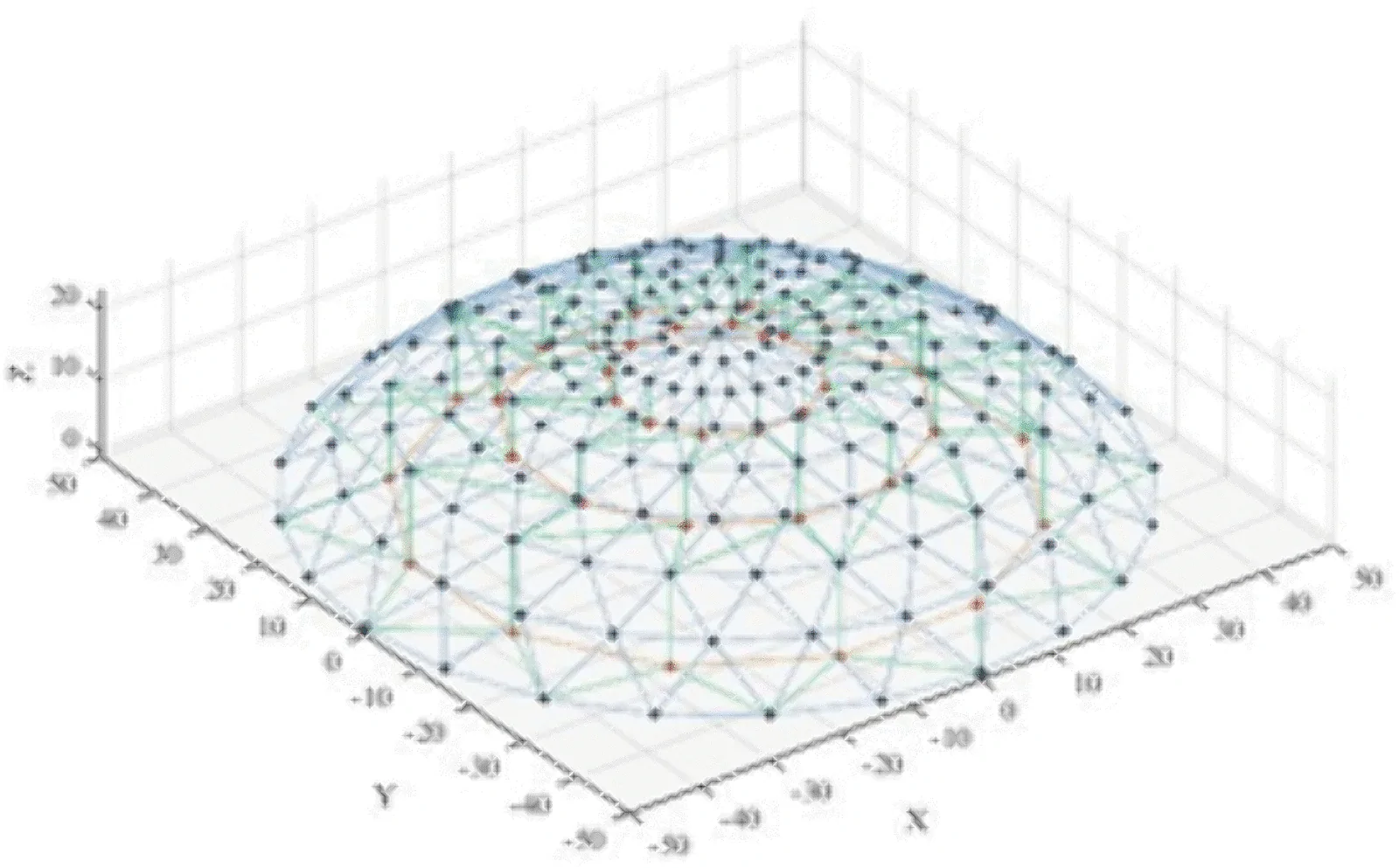
Basic Topology Optimization Model of the Suspen-Dome Structure.

### 3.2. New methods in topology optimization

#### 3.2.1. Cable force optimization method.

Reference [38] indicates that when the prestress distribution of the lower cable-strut system in a Suspen-Dome structure is reasonable, the lower cable-strut system under external loads can be regarded as elastic supports with appropriate stiffness. In this approach, the elastic modulus of the cables is multiplied by a certain factor while the initial strain is set to zero. The structural static response under specific loading conditions is then computed, and the internal force values of the lower cables are extracted and directly used as the design prestress values of the structural cables. This method is referred to as the *elastic support method*. However, Reference [38] also points out that when the internal forces obtained through the elastic support method are directly or indirectly used as the prestress design of the structural cables, the resulting prestress level tends to be excessively high.

The conventional prestressing method involves identifying a set of initial strains such that, under the combined action of prestress and self-weight, the internal forces in the cables of the cable-strut dome are equal to the design values. This approach, known as the tension compensation method, relies heavily on the specification of the initial strains, making it difficult to obtain accurate prestress values.

Fig 6 illustrates the proposed method, which integrates the tension compensation method with the elastic support method. First, the elastic support method is employed to control the cable forces within the vicinity of their target values; subsequently, the tension compensation method is applied in an iterative manner to obtain more accurate prestress values. This hybrid approach not only addresses the issue of overestimated prestress levels associated with the elastic support method but also mitigates the excessive dependence on initial strain assumptions inherent in the tension compensation method.

**Fig 6:**
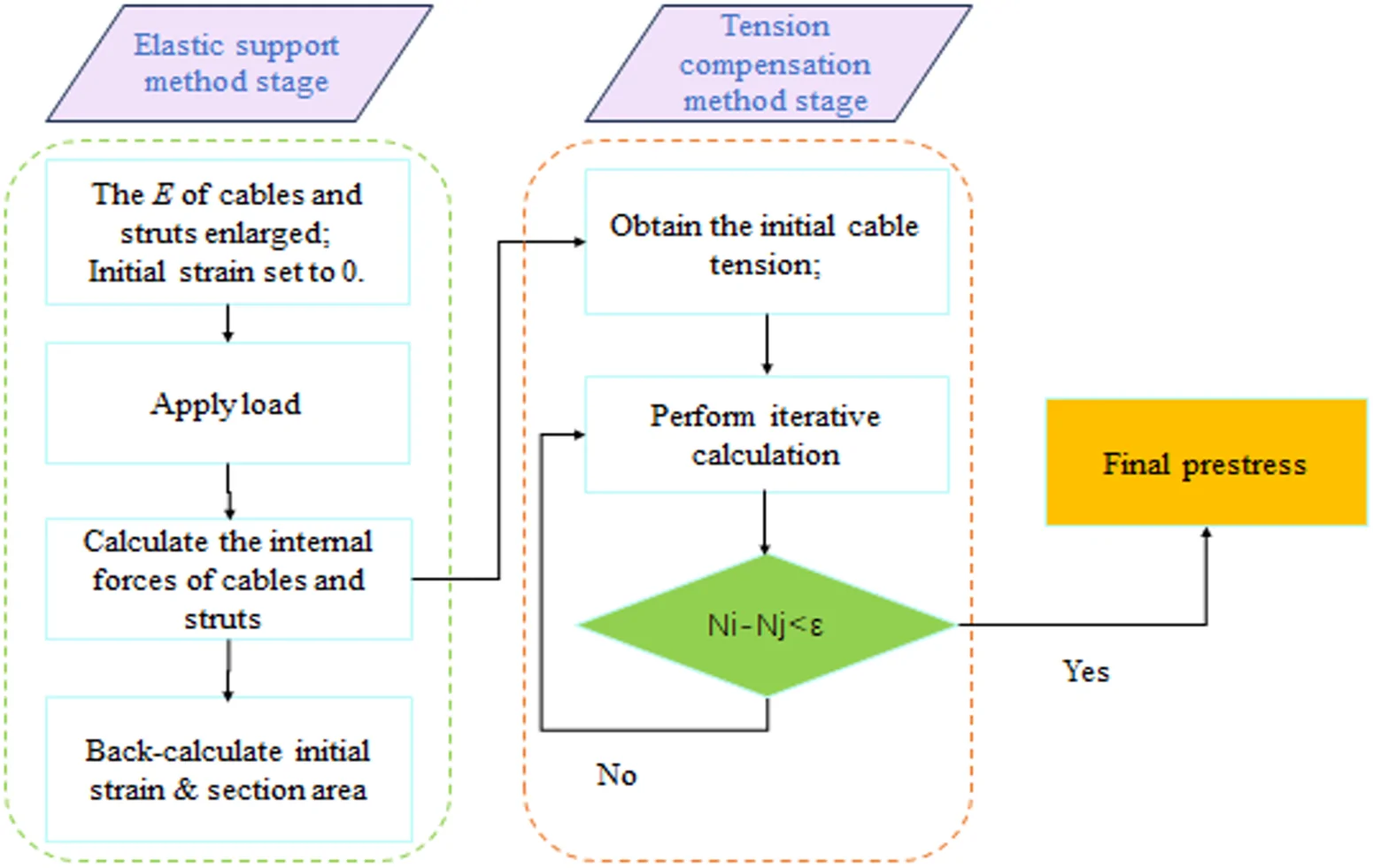
Flowchart of Prestress Calculation Methods.

#### 3.2.2. Pointer method based on BESO concept.

The Bi-directional Evolutionary Structural Optimization (BESO) method, proposed by Xie in 1997, aims to iteratively modify the topology toward an optimal configuration. Building upon the BESO concept, this study proposes a *pointer method* that adjusts structural stiffness and topology parameters to achieve optimal performance after topology optimization. The specific approach is described as follows:

(1) **Pointer Method for Topology Parameters**

With structural weight minimization and structural stiffness maximization as the optimization objectives, a composite function Φ is defined as follows:


$$\begin{array}{c} \Phi =\omega_{\text{1}}\overset{―}{K}+\omega_{\text{2}}\overset{―}{W} \end{array}$$ (14)

where


$$\begin{array}{c} \overset{―}{K}=\frac{K}{K_{\text{0}}} \end{array}$$ (15)

$$\begin{array}{c} \overset{―}{W}=\frac{W_{\text{0}}-W}{W_{\text{0}}} \end{array}$$ (16)

Here, a sensitivity analysis is conducted. All variables of the composite function Φ depend on the member quantity (density) parameter ρ. The derivative of Φ with respect to the topology variables is thus evaluated as:


$$\begin{array}{c} \frac{\partial \Phi }{\partial \rho }=\omega_{\text{1}}\frac{\partial \overset{―}{K}}{\partial \rho }+\omega_{\text{2}}\frac{\partial \overset{―}{W}}{\partial \rho } \end{array}$$ (17)

That is, when $\frac{\partial \Phi }{\partial \rho }>\text{0}$, increasing the number of topological components may improve the value of the composite function; conversely, when $\frac{\partial \Phi }{\partial \rho }<\text{0}$, reducing the number of topological components may improve the value of the composite function. According to the current iteration value, if the number of topological components is increased in this iteration, it indicates that, under the current weight setting, a greater number of members is beneficial, and this direction can be maintained. If the number of topological components is reduced in this iteration, it indicates that a smaller number of members is beneficial, and the adjustment direction needs to be reversed. In addition, the contributions of stiffness and weight need to be examined. If the decrease in stiffness is dominant, the number of topological components should be increased to enhance stiffness; if the increase in weight is dominant, the number of topological components should be reduced to decrease redundancy. Here, $\frac{\partial \Phi }{\partial \rho }$ does not represent the element density sensitivity in the traditional density method, but rather a heuristic indicator used to determine the direction of topological increase or decrease.

The standard gradient of this optimization problem is expressed as:


$$\begin{array}{c} \rho_{i}^{j+\text{1}}=\rho_{i}^{j}+\alpha \frac{\partial \Phi }{\partial \rho_{i}} \end{array}$$ (18)

The coefficients in function Φ are determined by the stiffness value and weight value of the initial model. Because the magnitudes of stiffness and weight differ substantially, using conventional weighting methods would render the weight contribution negligible, thereby failing to influence the pointer direction. To address this issue, a piecewise weighting scheme is adopted to dynamically adjust the composite function value, thus enabling real-time regulation of the pointer direction. The specific function is given as follows:


$$\begin{array}{c} \Phi =\left\{ \begin{array}{c} @r\omega_{\text{1}}\frac{K}{K_{\text{0}}}+\omega_{\text{2}}\frac{W_{\text{0}}-W}{W_{\text{0}}}\;if\;W\leq W_{\text{0}}\\ \omega_{\text{2}}\frac{K}{K_{\text{0}}}+\omega_{\text{1}}\frac{W_{\text{0}}-W}{W_{\text{0}}}\;if\;W>W_{\text{0}} \end{array}\right. \end{array}$$ (19)

Here, $\omega_{\text{1}}$ and $\omega_{\text{2}}$ are obtained from engineering experience, with $\omega_{\text{1}}$=0.7 and $\omega_{\text{2}}$=0.3, respectively. After applying this piecewise dynamic weighting adjustment, stiffness is prioritized as long as the structural weight does not exceed its value from the previous iteration, while weight considerations are simultaneously incorporated. Once the weight exceeds the allowable threshold, weight reduction is enforced even at the expense of partially sacrificing structural stiffness. This strategy prevents stiffness from completely dominating the optimization direction and enables the pointer method for multi-objective optimization.

(2) **Pointer Method for Stiffness Parameters**

The above approach provides a regulation strategy at the macroscopic level of the objective function. Here, within the inner optimization loop, the pointer method is employed to iteratively adjust the structural stiffness parameters.


$$\begin{array}{c} \Psi =\left\{ \begin{array}{c} @r\frac{K_{i}-K_{\text{0}}}{K_{\text{0}}}\geq \text{5}\%\;id=id+\text{1}\\ -\text{5}\%<\frac{K_{i}-K_{\text{0}}}{K_{\text{0}}}<\text{5}\%\;id=id\\ \frac{K_{i}-K_{\text{0}}}{K_{\text{0}}}\leq -\text{5}\%\;id=id-\text{1} \end{array}\right. \end{array}$$ (20)

Fig 7 illustrates that if the positive stiffness error exceeds 5%, the pointer moves toward increasing stiffness; if the negative error exceeds 5%, it moves toward reducing stiffness; and if the error remains within the permissible range, the pointer position remains unchanged.

**Fig 7:**
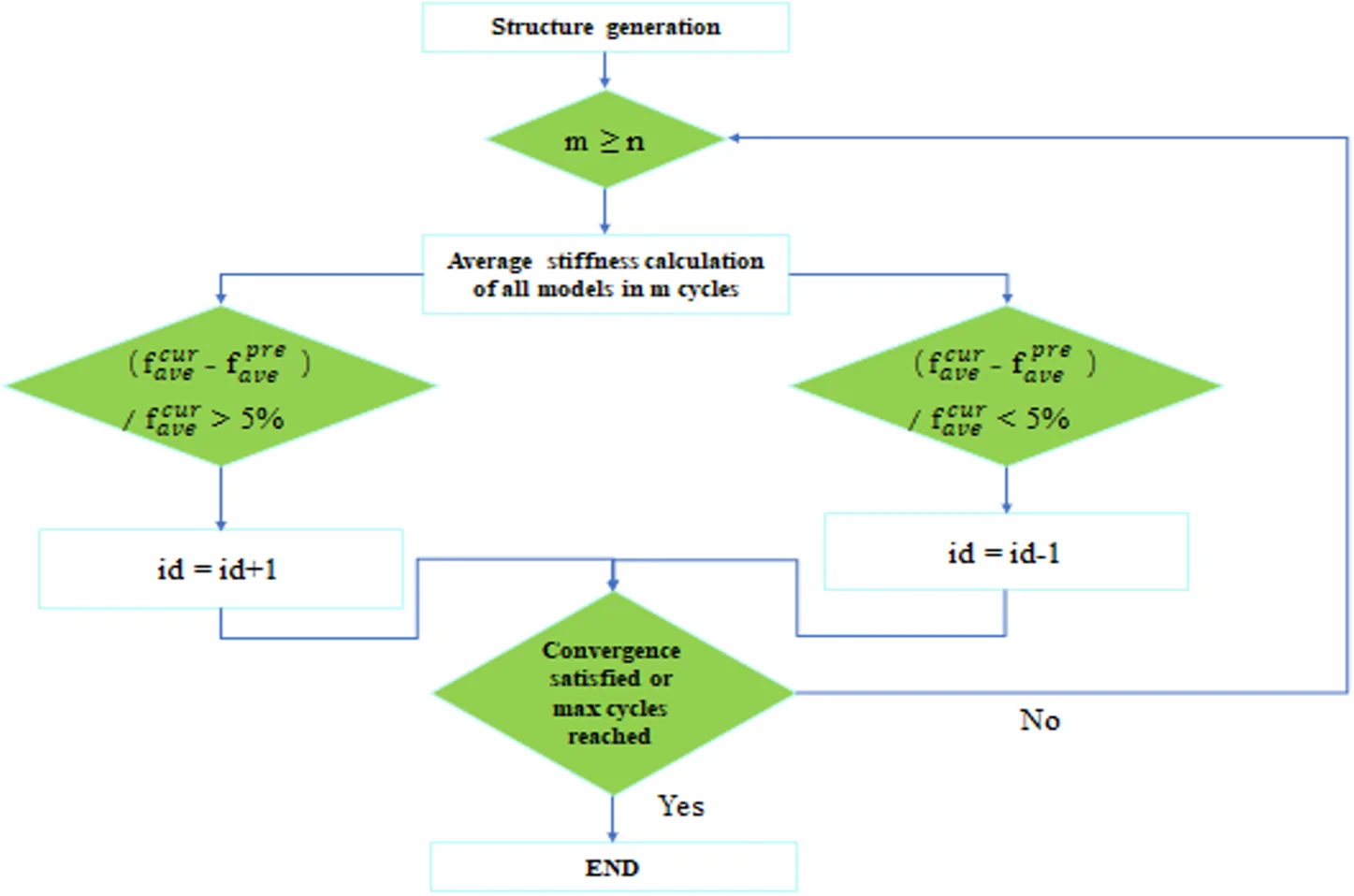
Pointer Method for Stiffness Parameters.

#### 3.2.3. Multi-objective optimization based on SMM-iPGSA.

Currently, the PGSA method involves only single-objective optimization, typically focusing on either structural weight or horizontal stress. In this study, an improved PGSA method is employed to simultaneously optimize structural weight and stiffness, marking the first application of PGSA to multi-objective structural optimization.

Moreover, the optimization process explicitly considers global stability and jointly optimizes the cross-sectional areas and lengths of the struts and ties.

#### 3.2.4. Partial full-stress design.

Considering that the Suspen-Dome consists of an upper reticulated shell and a lower cable-strut system, which differ markedly in their load-bearing mechanisms and structural functions, this study proposes a Partial Full-Stress Design (PFSD) strategy. Its basic concept is not to apply the full-stress design criterion uniformly to the entire structure, but rather to adopt different design criteria according to the mechanical characteristics of the individual subsystems. The lower cable-strut system primarily provides prestress and regulates the internal-force distribution of the structure. Because the cables mainly sustain axial tension and are not susceptible to compressive buckling, adopting a near-full-stress design enables the material properties of high-strength steel cables to be more fully utilized, thereby improving prestress efficiency and material utilization. By contrast, the upper reticulated shell not only transfers vertical loads but also provides overall structural stiffness and stability reserve. The behavior of its members is jointly influenced by overall stability, local buckling, and initial geometric imperfections. A simple application of the full-stress design principle may therefore result in excessively small member sections, thereby reducing structural stiffness and stability reserve. Accordingly, an appropriate stress margin is retained for the upper reticulated shell to ensure sufficient overall stiffness and stability. Therefore, PFSD is not a new structural theory, but a design strategy developed on the basis of the “rigid–flexible interaction” characteristics of the Suspen-Dome. Its purpose is to improve the material utilization efficiency of the lower cable-strut system while maintaining an adequate safety reserve in the upper reticulated shell, thereby achieving a balance between structural lightweighting and safety.

#### 3.2.5. Neural-network-assisted candidate screening strategy.

To reduce repeated finite-element analyses in the SMM-iPGSA optimization process, a lightweight multilayer perceptron (MLP) classifier was introduced as a candidate screening model. The model was not used to replace high-fidelity structural analysis, but only to remove clearly infeasible candidates before ANSYS verification.

The input vector of the MLP was defined as


$$\begin{array}{c} x=[rings,\;cut,\;q_{input},\;{sec}_{max},\;{sec}_{min},\;{sec}_{mean},\;{sec}_{var},\;{sec}_{std}] \end{array}$$ (21)

where rings and cut denote the number of rings and circumferential divisions of the reticulated shell, respectively; $q_{input}$ is the mapping parameter; and ${sec}_{max},\;{sec}_{min},\;{sec}_{mean},\;{sec}_{var},\;{sec}_{std}$ are the statistical descriptors of the section IDs. The output was the feasibility probability of the candidate section combination. A sample was labelled as feasible only when all displacement, strength, slenderness, and stability constraints were satisfied.

The MLP consisted of three hidden layers with 64, 32, and 16 neurons, respectively. ReLU activation functions were adopted in the hidden layers, and the model was trained using the binary cross-entropy loss and the Adam optimizer. The batch size, initial learning rate, weight-decay coefficient, and maximum number of training epochs were set to 64, 0.001, 0.00001, and 200, respectively. Early stopping with a patience of 30 epochs was employed to reduce overfitting. A probability threshold of 0.2 was used to convert the predicted feasibility probability into a binary label. Since the purpose of the classifier was to avoid incorrectly eliminating feasible candidates, the relatively low threshold was adopted to prioritize the recall of feasible samples. The same threshold was used in model validation and subsequent candidate screening. The dataset consisted of candidate structures generated during the SMM-iPGSA optimization process and contained 1,348 valid samples, including 809 feasible samples and 539 infeasible samples. All samples were analyzed using ANSYS. A sample was labeled as feasible only when the displacement, strength, slenderness-ratio, and stability constraints were all satisfied.

The testing results are shown in Fig 8. The MLP achieved an accuracy of 0.9212, a precision of 0.9467, a recall of 0.9581, and an F1-score of 0.9524. Therefore, the model can effectively preserve most feasible candidates while eliminating part of the infeasible candidates before ANSYS analysis.

**Fig 8:**
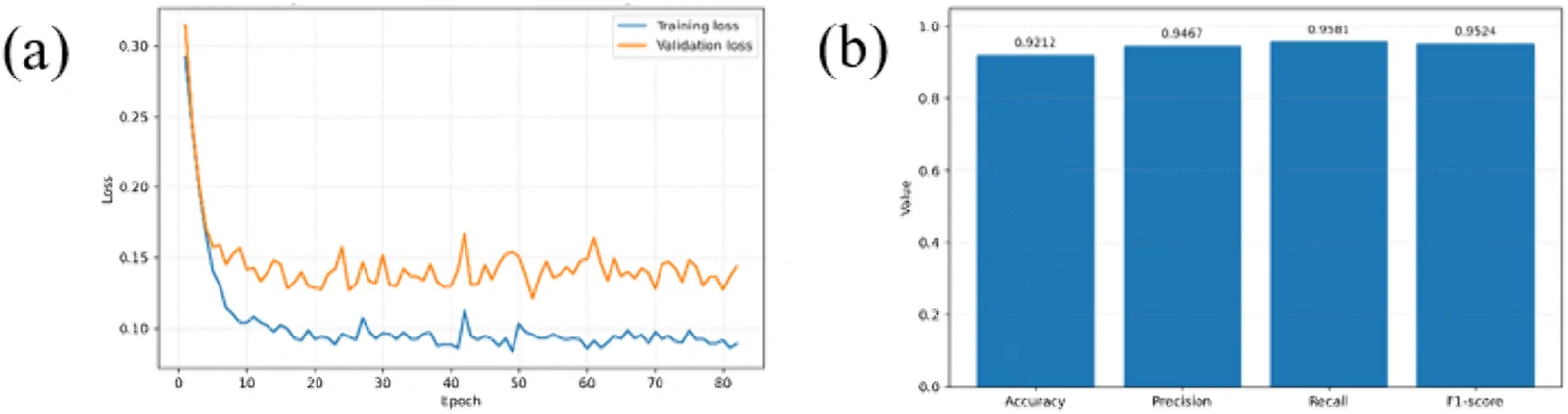
Training convergence and testing performance of the MLP screening model. (a) Loss curve. (b) Testing performance.

To evaluate the sensitivity of the model to dataset partitioning, five-fold stratified cross-validation was further conducted. The mean values and standard deviations of the four classification metrics are listed in Table 4, indicating that the screening performance remained stable under different data partitions.

**Table 4:** Average computational time.

	Independent testing result	Five-fold cross-validation
Accuracy	0.9212	0.9120 ± 0.0056
Precision	0.9467	0.9234 ± 0.0130
Recall	0.9581	0.9636 ± 0.0042
F1-score	0.9524	0.9431 ± 0.0054

Since the inference time of the MLP is negligible compared with ANSYS analysis, the reduction in ANSYS calls can be regarded as the main source of computational acceleration. The average computational time was reduced by approximately 25.6%. Therefore, the proposed MLP-assisted screening strategy can improve optimization efficiency while retaining ANSYS verification for final candidate solutions. The second case study in Section 2.3 was used to evaluate the computational efficiency of the MLP-assisted screening strategy. In Table 5, this can be illustrated that the average computational time was reduced from 716 s without MLP acceleration to 533 s with MLP acceleration.

**Table 5:** Average computational time.

	Average computational time (s)
MLP-assisted acceleration	533s
Without MLP acceleration	716s

### 3.3. Topology optimization workflow

In this study, MATLAB and ANSYS are integrated to perform topology optimization of prefabricated long-span cable-strut domes based on the mapping method and the improved PGSA algorithm. The optimization process is illustrated in Fig 9.

**Fig 9:**
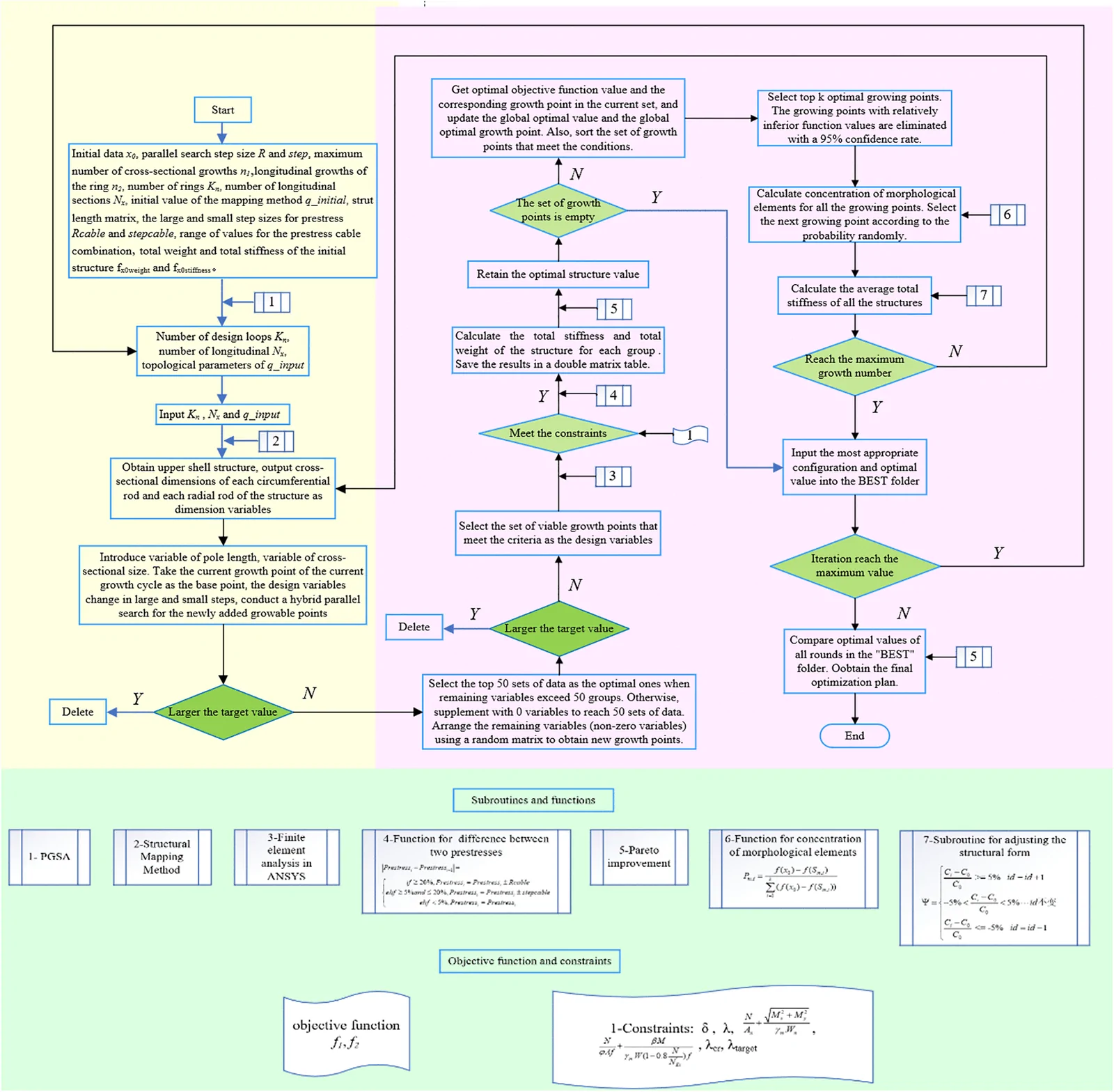
Topology Optimization Computational Workflow.

The workflow is divided into two main program modules: the algorithmic procedures are implemented in MATLAB, while structural analysis and result output are performed in ANSYS.

**MATLAB Program Workflow**

The MATLAB program for the topology optimization proceeds as follows:

1 **Initialization**

Input the initial structural information. Define the initial data $x_{\text{0}}$, the step sizes ***R*** and ***step*** for the parallel search, the maximum growth iterations $n_{\text{1}}$ for cross-sections and $n_{\text{2}}$ for rings and radials, the number of rings ***Kn*** and radials ***Nx***, the initial mapping value $q_{initial}$, the strut length matrix, the large and small step sizes for prestress ***Rcable*** and ***stepcable***, the range of prestress combinations, as well as the initial total structural weight ${fx}_{\text{0}weight}$ and stiffness ${fx}_{\text{0}stiffness}$.

2 **Outer Loop**

For the given topological parameters ***Kn*** (number of rings) and ***Nx*** (number of radials), apply the small-scale PGSA to determine their optimal values.

3 **Mapping**

Generate the upper reticulated shell structure using the mapping method and export the cross-sectional dimensions of each circumferential and radial member as size optimization variables.

4 **Parallel Search Expansion**

Introduce the strut length and cross-section variables. Around the current growth point, vary the design variables using large and small step sizes. Perform hybrid parallel search to generate new candidate growth points, discard variables exceeding target thresholds, and retain the remaining candidates.

5 **Candidate Filtering**

If more than 50 candidate sets remain, retain only the top 50; otherwise, supplement with zero variables to reach 50 sets. Apply random matrices to redistribute remaining non-zero variables, remove infeasible or duplicate candidates, and perform a second screening to discard variables exceeding the target values.

6 **Structural Analysis**

The preliminarily filtered growth-point set is first evaluated by the MLP-based candidate screening model, and candidates with low feasibility probabilities are discarded. The remaining candidates are submitted to ANSYS for structural analysis, where displacement, internal force, stress, and stability responses are obtained for final constraint checking. Only candidates satisfying all constraints are retained.

7 **Prestress Adjustment**

Compare the current and previous prestress values. If the difference exceeds 5%, adjust the initial prestress ***Rcable***; if the difference lies between 1% and 5%, adjust ***stepcable*** instead.

8 **Objective Evaluation**

Compute the total stiffness and weight for each candidate structure and record them in the table. Apply Pareto optimization to retain the best solutions.

9 **Feasibility Check**

If no feasible candidate remains, proceed directly to Step 15; otherwise, continue.

10 **Global Best Update I**

Identify the best objective value and corresponding candidate set. Update the global best solution and sort the feasible set accordingly.

11 **Global Best Update II**

Reconfirm the best objective value and global optimum after sorting the feasible set.

12 **Top-k Retention**

Retain only the top k candidates; discard the others by eliminating 5% of the worst-performing solutions and proceed to Step 13.

13 **Morphogen Concentration Sampling**

For all retained candidates, compute morphogen concentrations and randomly select growth points for the next iteration based on assigned probabilities.

14 **Pointer Adjustment**

Calculate the average stiffness of all structures in the current round. If the difference between consecutive rounds exceeds 5%, adjust the pointer toward the better structure; otherwise, retain the current pointer direction.

15 **Iteration Check I**

If the maximum number of growth iterations $n_{\text{1}}$ is reached, proceed to Step 16; otherwise, return to Step 4.

16 **Result Storage**

Save the best structural configuration and objective value in the *BEST* folder for the current topology.

17 **Iteration Check II**

If the maximum number of outer iterations is reached, proceed to Step 18; otherwise, restart from Step 2 with updated pointer directions.

18 **Final Pareto Optimization**

Perform a final Pareto optimization across all iterations using the results stored in the folder to obtain the global optimal solution.

To further clarify the algorithm implementation parameters, the maximum capacity of the candidate pool used for selecting the next growth point in each iteration was set to k = 50. Duplicate solutions, out-of-bound solutions, and solutions that violated the prescribed topological rules were first removed, after which the remaining candidates were preliminarily screened using the MLP model. The MLP classification threshold was set to 0.2. Candidate structures with a predicted feasibility probability of no less than 0.2 were submitted to ANSYS for finite-element verification, whereas the remaining candidates were discarded in advance. Candidate solutions satisfying all structural constraints were further subjected to Pareto non-dominated analysis, in which dominated solutions were removed and non-dominated solutions were added to the updated Pareto solution set. No maximum capacity was imposed on the Pareto solution set, whereas k = 50k = 50k = 50 was used only to limit the number of candidates participating in the selection of the next growth point. The candidate pool was ranked in descending order according to morphogen concentration. The maximum numbers of iterations for the inner optimization loop and the outer topology loop were set to 300 and 10, respectively. The corresponding loop was terminated when the maximum number of iterations was reached or when the set of valid candidates became empty. No additional objective-value-stability criterion was adopted in this study.

## 4. Topology optimization of prefabricated kiewitt–lamella long-span Suspen-Dome

### 4.1. Initial structure

The initial structure has a span of 100 m, a rise of 25 m, and a rise-to-span ratio of 1/4. Steel members are Q345B tubular sections with a design strength of f =310N/mm^2^, Poisson’s ratio ν= 0.3, density ρ = 7850 kg/m^3^, and elastic modulus E_1_ = 2.06 × 10^5^N/mm^2^. Prefabricated joints are employed [39]. The cables are made of Grade 1670 semi-parallel steel wire strands with an elastic modulus of E_2_ = 1.9 × 10^5^N/mm^2^ and an ultimate tensile strength of f_u_ =1670N/mm^2^. According to the design code, the allowable design strength of cables should not exceed 40–55% of the ultimate tensile strength. Here, 40% is adopted, giving a maximum allowable cable stress of 668 N/mm^2^ under all load cases.

To ensure the practical constructability of the generated topologies, the number of circumferential divisions was determined according to the grid-size control principle, such that the circumferential and radial member lengths remained within the same order of magnitude, approximately 3–8 m. Accordingly, the 100 m-span Kiewitt–Lamella Suspen-Dome considered in this section adopted a division scheme of [8,26], whereas the 110 m-span rib-ring Suspen-Dome examined in Section 5 adopted an equal-division scheme of [20,40]. These schemes produced relatively uniform grid cells while balancing member stability, uniformity of structural response, and construction economy.Moreover, to maintain structural symmetry, ***Kn*** must take even values. For the single-layer reticulated shell, the SMM is employed to determine the layout of each ring of members. To guarantee the rational length of radial members, the feasible domain of the number of rings ***Nx*** is restricted to [8,15]. For the lower cable-strut system, the topological variable ***interval*** represents the number of skipped rings between successive cable layers. The value of ***interval*** is defined as an integer within the range [0, n], where n is the maximum number of skipped rings determined by the upper shell ring number ***Nx***. The maximum value of ***n***_***1***_ is 4. Considering the layout of radial cables in the Lamella configuration, the feasible value of *interval* is also related to the circumferential division number ***Kn***, with a maximum value of ***n***_***2***_ = 6.

The prestress of the lower cable-strut system is initially determined using the elastic support method. The cross-sections of the struts and ties are then back-calculated based on the internal forces obtained from this method, while the cross-sectional areas of the prestressed cables are determined according to the layout of the cable system. With fewer cable rings, the cable cross-sectional area increases progressively from the inner ring to the outer ring. After the initial prestress of the cables is assigned, an iterative procedure is employed to determine the precise prestress values. To prevent cable relaxation, the minimum initial prestress is set to 100 kN.

### 4.2. Constraint conditions and layout of optimization parameters

The constraints are divided into two categories: (i) member-level constraints and (ii) overall stability constraints, as specified in Section 2.

When performing topology optimization of the Suspen-Dome using the SMM-iPGSA, the initial configuration is set as follows: the circumferential division number ***Nx*** = 20, the number of rings ***Kn*** = 10; all groups of members are initially assigned cross-section type No. 60; the initial input parameter ***q_input_initial*** = 2000. The large step size ***R*** = 3, the small step size ***step*** = 1; the prestress adjustment large step size ***Rcable*** = 30 kN and small step size ***stepcable*** = 10 kN. The equivalent nodal load is applied as F=−150 kN. The initial ***interval*** of the cable-strut system, both radially and circumferentially, is set to zero. The maximum number of iterations in each inner loop is 300, and the maximum number of big iterations is 10.

The initial weight of the structure is 1.38 × 10^3^ t, and the initial stiffness is 4.60 × 10^11^. With the minimization of total structural weight and the maximization of total structural stiffness as the optimization objectives, the mathematical model is defined in Section 3.1. The initial structural configuration is shown in Fig 10.

**Fig 10:**
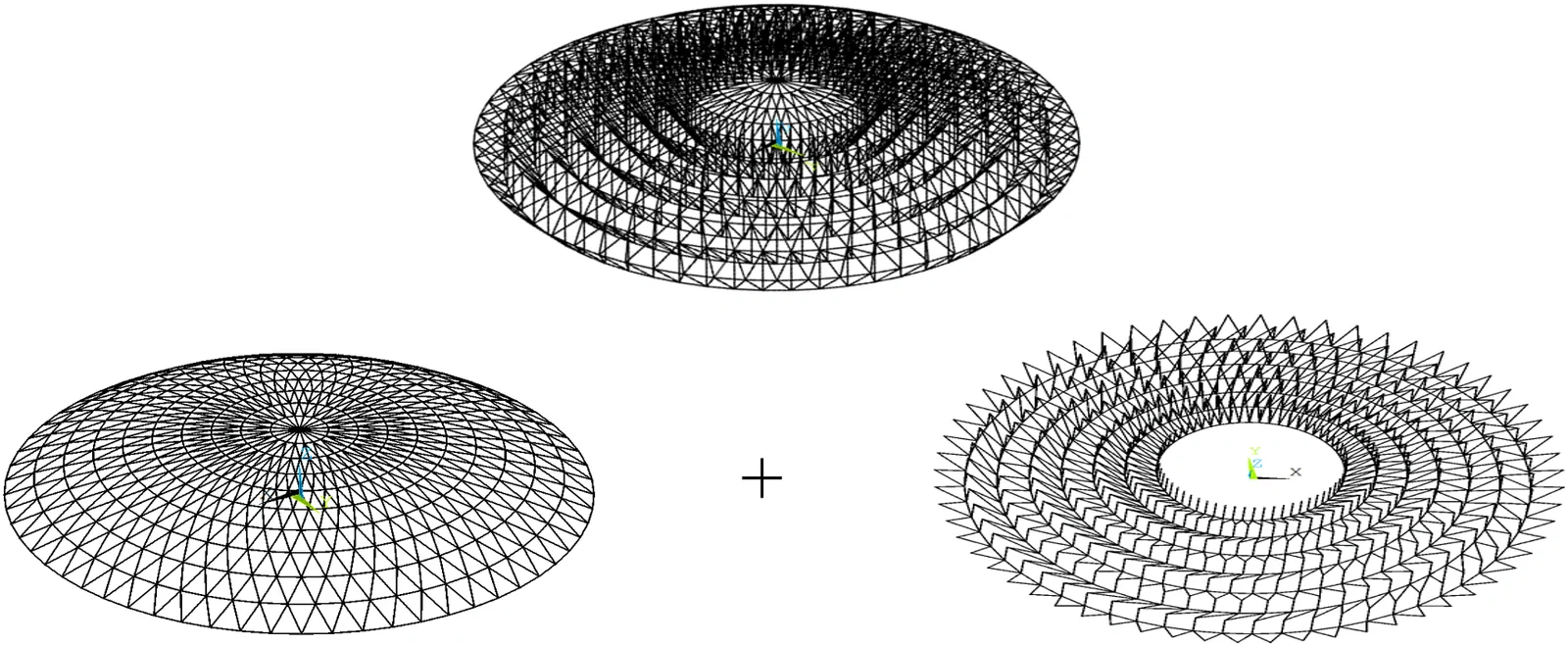
Initial Suspen-Dome model. (span = 100 m, rise = 25 m, ***Nx*** = 20, ***Kn*** = 10).

Figs 11 and 12 illustrate the evolution of the structural configuration and parameter adjustments of the Kiewitt–Lamella Suspen-Dome during the topology optimization process. After six major iterations, the o ptimal solution and corresponding configuration were identified. The detailed optimization results are presented in Table 6.

**Table 6:** Topology optimization results of the prefabricated long-span Kiewitt–Lamella Suspen-Dome.

Optimization result	
Span	100m
Rise	14.30m
Rise-to-span ratio	0.1430
***Nx***	12
***Kn***	11
Radial interval number	1
Circumferential interval number	3
Cable prestress design values for each ring (kN, from inner to outer)	142.26, 198.02, 188.82, 211.63
Strut lengths of each ring (m)	6, 6, 6, 7
Strut cross-sectional areas of each ring (cm^2^)	50.70, 50.70, 50.70, 69.10
Tie lengths of each ring (m)	11.91, 13.45, 15.59, 18.19
Tie cross-sectional areas of each ring (cm^2^)	71.30, 90.90, 122.00, 166.00
Total number of members in the reticulated shell	1044
Section IDs of circumferential members (from inner to outer)	39,40,40,43,43,41,39,41,41,41,39
Section IDs of radial members (from inner to outer)	41,40,37,40,43,39,40,40,40,37,43
Maximum vertical displacement	−20.90 mm
Total structural mass	271.56
Nonlinear buckling safety factor	3.32
Eigenvalue buckling safety factor	8.15
$q_{input}$	1800

**Fig 11:**
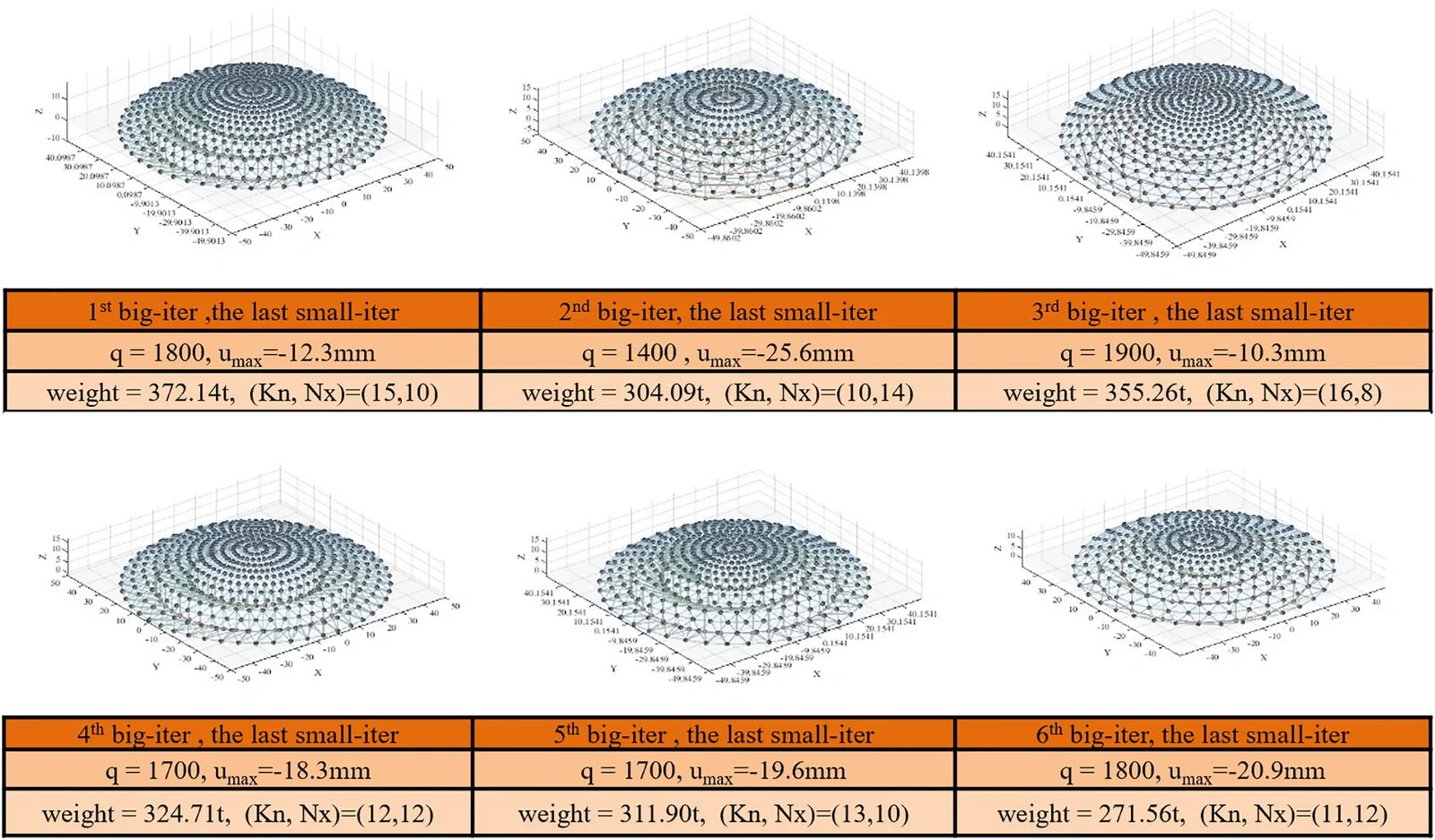
Topology-optimized configuration and parameter variations of the prefabricated long-span Kiewitt–Lamella Suspen-Dome.

**Fig 12:**
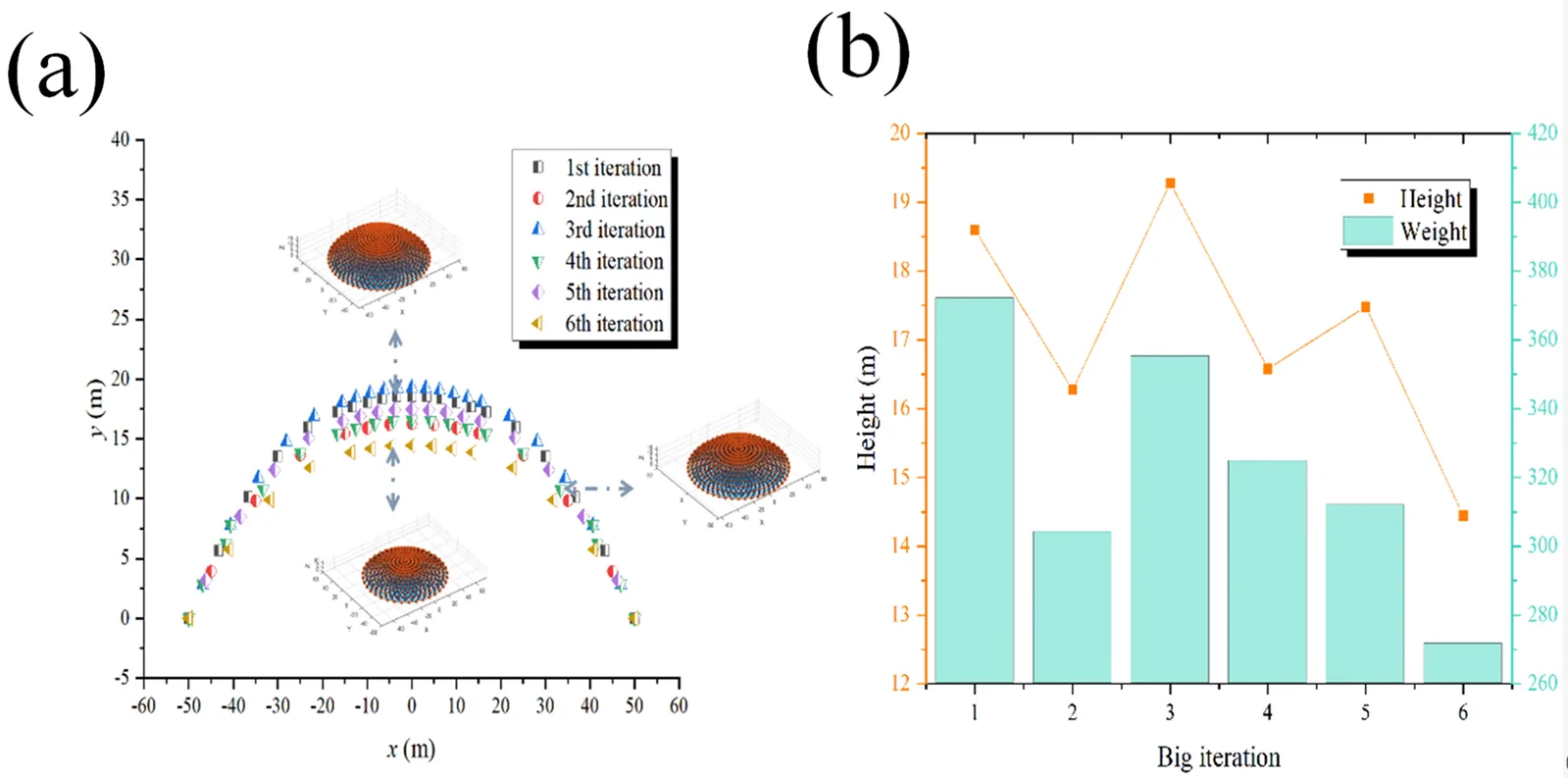
Parameter variations during the topology optimization process. (a) geometric configuration changes in major iterations. (b) variations of rise and weight in major iterations.

To eliminate the mass-scale effect, this study employs the stiffness-to-weight ratio ***K***_***spec***_ ***= K/W*** to evaluate the integrated efficiency of “lightweight–stiff” design, where ***W*** denotes the structural weight and ***K*** the stiffness. Fig 13(a)shows that, as the topology and cross-sections were progressively over six major iterations, ***K***_***spec***_ exhibited a fluctuating upward trend and reached its maximum in the sixth iteration. This indicates that the final scheme achieves higher equivalent stiffness under the same weight (or lower self-weight for the same stiffness requirement), thus yielding optimal material utilization efficiency. It should be emphasized that all candidate schemes satisfy displacement, stress, and buckling constraints; therefore, the observed improvement does not result from sacrificing safety margins, but rather from more direct load paths and more rational cross-sectional and prestress distributions.

**Fig 13:**
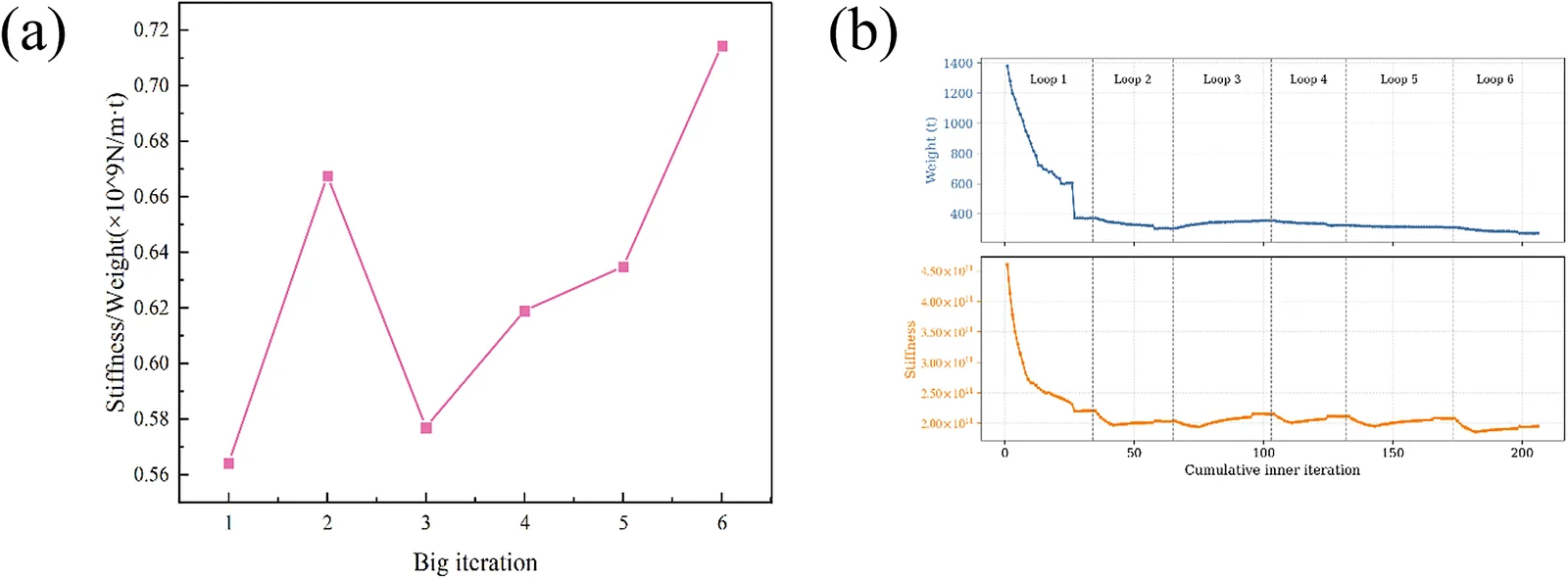
Structural performance index and convergence behavior during optimization. (a) Stiffness-to-weight ratio of the structure. (b) Convergence curve.

Fig 13(b) shows that, for the Kiewitt-Lamella Suspen-Dome structure, the arrangement of members in the upper reticulated shell is relatively regular, and the overall force transmission path of the structure is relatively clear. Therefore, at the initial stage of optimization, with the rapid adjustment of member cross-sections and topological parameters, the structural weight decreases rapidly, and the stiffness-to-weight ratio is significantly improved. Meanwhile, since the lightweighting process weakens the local stiffness to a certain extent, the stiffness index in the convergence curve first decreases. Subsequently, under the combined action of the Structural Mapping Method and the pointer adjustment mechanism, the optimization process is gradually corrected toward a more favorable topological direction, and the stiffness is restored to a certain extent and tends to become stable. This result indicates that the proposed method can effectively coordinate the contradiction between lightweight design and stiffness retention in this type of Suspen-Dome structure with regular topology.

As shown in Table 6, the optimal topology yields a total steel consumption of 271.56 t under the given load, corresponding to a unit steel usage of 34.6 kg/m^2^. This represents a significant saving in steel while ensuring structural safety.

The prestress values of the four circumferential cable rings are 142.26 kN, 198.02 kN, 188.82 kN, and 211.63 kN, respectively, which are slightly lower than expected. The reason is that maximizing stiffness in the multi-objective function increases the stiffness contribution of the reticulated shell—which dominates overall stiffness—while reducing the relative stiffness of the cable-strut system, thereby leading to lower prestress values. Nevertheless, owing to the application of the SMM, the layout of the upper reticulated shell remains rational, resulting in reduced structural weight and simultaneously improved stiffness.

Under the optimal topology, the maximum vertical displacement is far below the code limit of 250 mm, indicating excellent structural stiffness. The eigenvalue buckling factor of 8.15 further demonstrates that the structure can withstand a relatively high critical load.

As shown in Fig 14, the histograms of stress-ratio distributions for the upper reticulated shell members in the optimized schemes exhibit approximately normal distributions, with stress ratios primarily concentrated in the ranges of 0.2–0.5, 0.3–0.6, and 0.3–0.5. In the final major iteration, the distribution becomes more concentrated around the midrange interval, and the peak is more pronounced, indicating that a greater proportion of members exhibit stress ratios close to the average value and thus experience more uniform stress.

**Fig 14:**
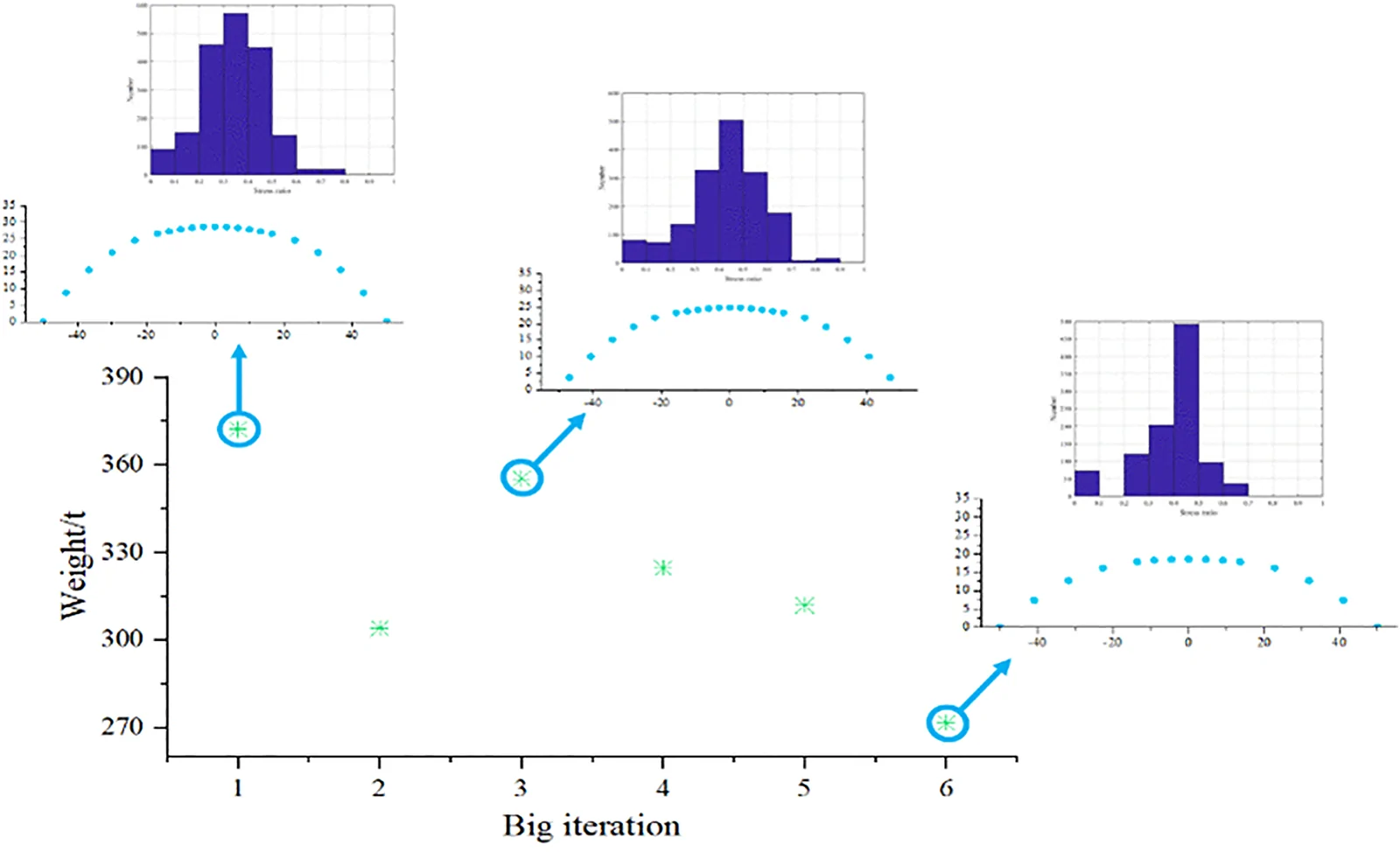
The optimum individuals of every final big iteration.

In line with the design philosophy of the structural system, the lower cable-strut system adopts a full-stress design to maximize material utilization, while the upper reticulated shell retains a certain safety reserve. This combined approach realizes a partial full-stress design, achieving a balance between minimizing weight and maximizing stiffness. Consequently, the optimized scheme not only ensures uniform stress distribution within the shell but also makes rational use of the residual load-bearing capacity of certain members, thereby attaining a superior trade-off between weight reduction and stiffness enhancement. This outcome is consistent with the overall optimization concept of Suspen-Dome structures.

For the prefabricated Kiewitt–Lamella Suspen-Dome with a span of 100 m, the optimal rise-to-span ratio under the dual objectives of minimum weight and maximum stiffness is approximately 0.14. The corresponding number of rings and circumferential divisions are preferably 12 and 13, respectively. These findings provide useful guidance for practical structural design and selection.

### 4.3. Static analysis of the optimized structure

The displacement and stress contour diagrams of the optimal topology are shown in Figs 15–17. In these Figs, the units are: length in meters (m), internal force in Newtons (N), and stress in Pascals (Pa). After optimization, none of the structural materials exceed the yield strength, and no stress concentration is observed, indicating that the structure remains in a safe state with an adequate safety reserve.

**Fig 15:**
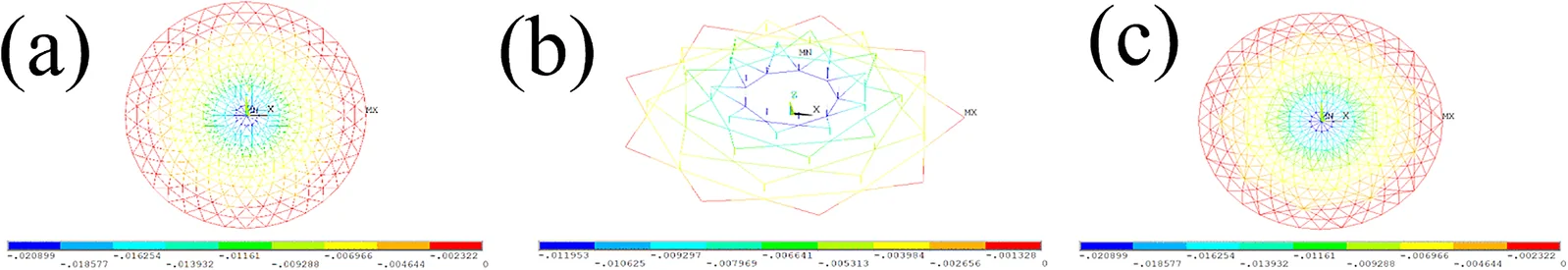
Displacement contours of the Kiewitt–Lamella Suspen-Dome. (a) upper reticulated shell. (b) cable-strut system. (c) entire structure.

**Fig 16:**
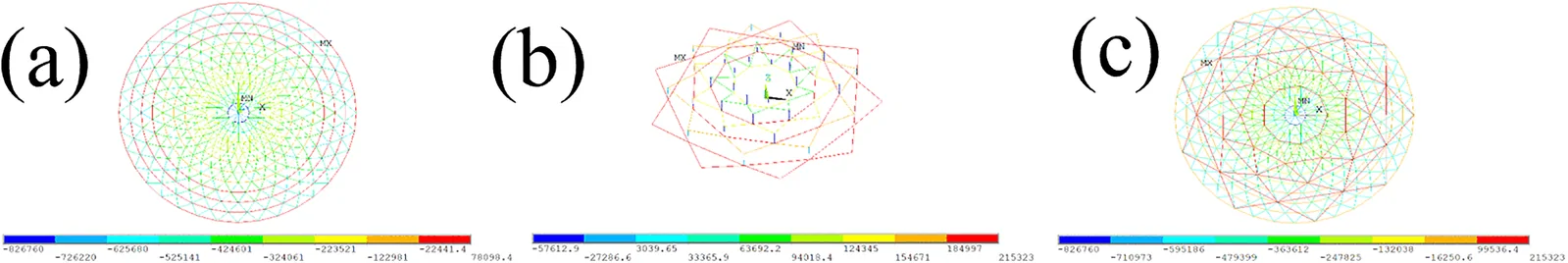
Internal force contours of the Kiewitt–Lamella Suspen-Dome. (a) upper reticulated shell. (b) cable-strut system. (c) entire structure.

**Fig 17:**
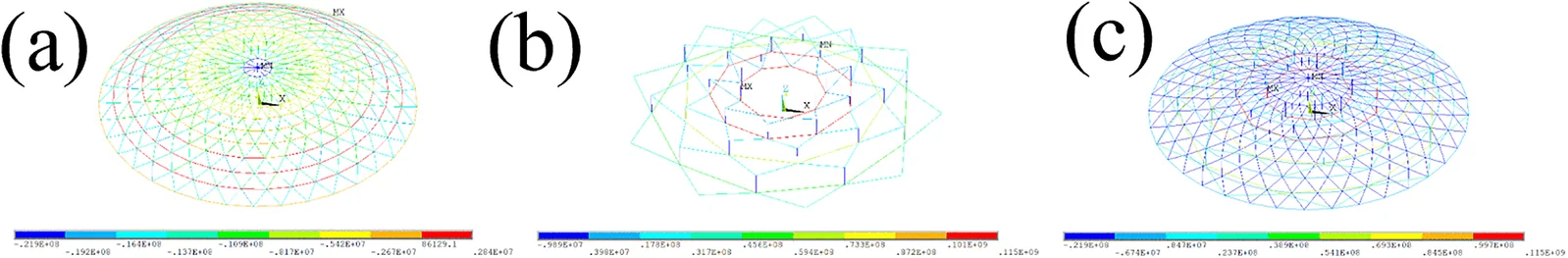
Stress contours of the Kiewitt–Lamella Suspen-Dome. (a) upper reticulated shell. (b) cable-strut system. (c) entire structure.

From the displacement contours, the vertical displacement of the reticulated shell is 20.9 mm, which is far below the code limit of 250 mm, demonstrating excellent stiffness performance. From the stress contours, the stress distribution of the reticulated shell is uniform with no concentration; circumferential members are in tension, while radial members are in compression. This indicates that under the SMM, the radial members primarily carry loads along the transfer direction, whereas the circumferential members control the transverse displacements at the joints by remaining in tension, thereby maintaining structural stability.

The cable-strut system shows small displacements without unfavorable deformations. Its stress state does not exceed the tensile limit, and the reinforcement strategy is consistent with the prestress design values. Overall, the optimized structure is safe, satisfies functional requirements, exhibits rational load transfer, and achieves efficient utilization of materials.

### 4.4. Modal analysis of the optimized structure

As shown in Fig 18, a modal analysis of the Kiewitt–Lamella Suspen-Dome was conducted for the first six modes, with frequencies ranging from 3.94 Hz to 5.93 Hz. The first mode corresponds to vertical symmetric vibration, while the second and third modes are characterized by vertical and horizontal bending. From the fourth to the sixth modes, no local instabilities are observed; instead, all are manifested as global instability. In these cases, the unstable regions tend to coordinate deformation with adjacent areas, propagating the instability to a wider region and ultimately resulting in global structural instability.

**Fig 18:**
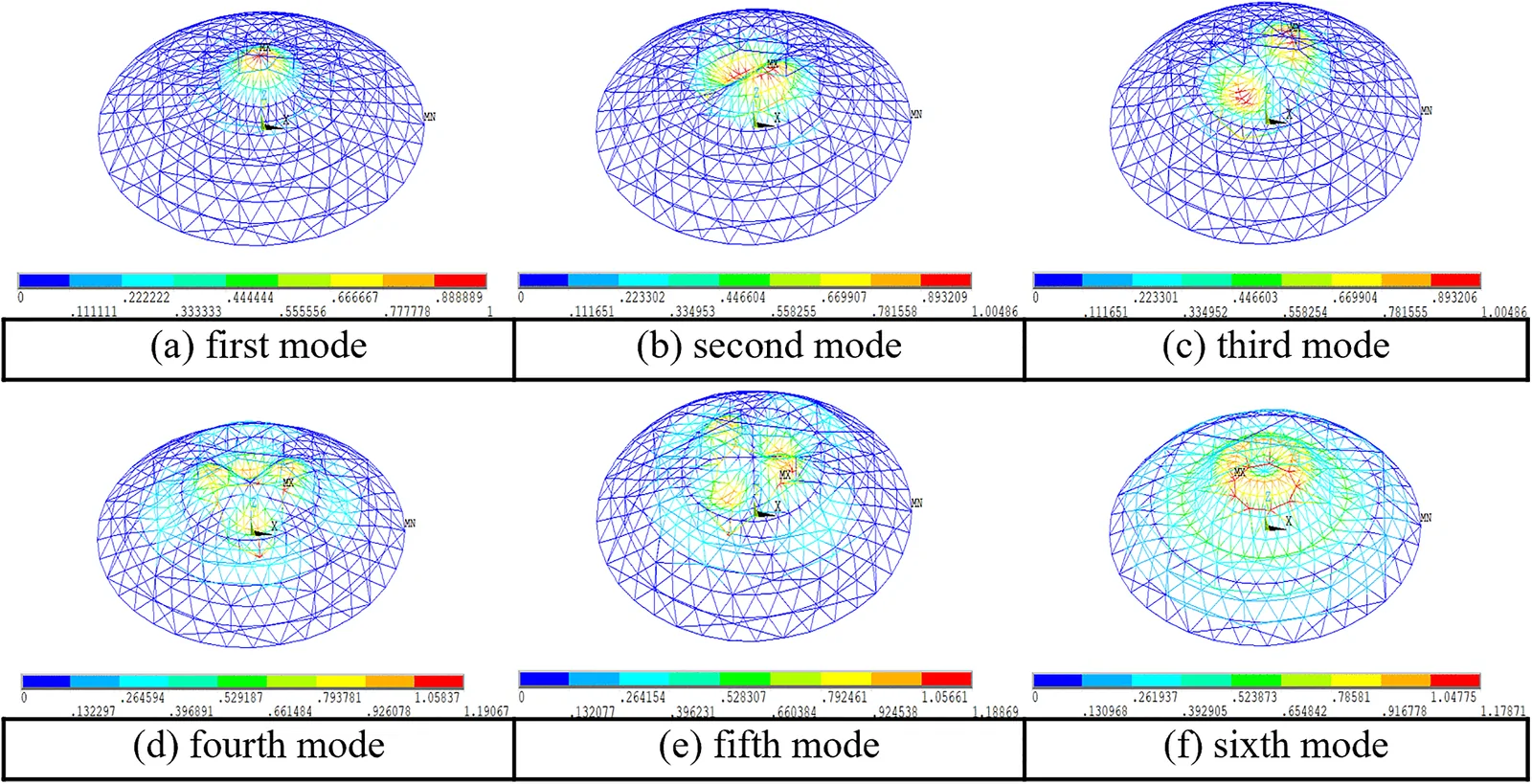
First six vibration modes of the Kiewitt–Lamella Suspen-Dome. (a) first mode; (b) second mode; (c) third mode; (d) fourth mode; (e) fifth mode; (f) sixth mode.

According to Table 7, The structural frequencies are relatively high and do not fall in the low-frequency range; however, they remain within the dominant seismic frequency band (0.5–10 Hz), and thus the potential influence of external seismic excitations should be taken into account.

**Table 7:** First six modal parameters of the prefabricated long-span Kiewitt–Lamella Suspen-Dome.

Modal order	Eigenvalue	Frequency
1	612.26	3.94
2	685.79	4.17
3	685.79	4.17
4	1236.79	5.60
5	1386.85	5.93
6	1386.85	5.93

## 5. Topology Optimization of prefabricated rib-ring long-span Suspen-Dome

### 5.1. Initial structure

The initial circumferential division number is set to ***Nx*** = 36, and the number of rings to ***Kn*** = 11. All groups of members are initially assigned cross-section type No. 60, and the mathematical model is defined in Section 3.1. The initial structure has a span of 110 m, a rise of 31 m, and a rise-to-span ratio of 0.28. To ensure that the length of the outermost circumferential members remains reasonable, the feasible domain of the circumferential division number ***Nx*** is restricted to [2040]. Similarly, to guarantee rational lengths of radial members, the feasible domain of the number of rings ***Kn*** is restricted to [8,15]. All other parameters are the same as those specified in Section 4.1.

### 5.2. Constraint conditions and parameter layout

The initial numbering of the cable-strut system is set to 1. The initial weight of the structure is 3.36 × 10^2^ t, and the initial stiffness is 2.33 × 10^11^. With the minimization of total structural weight and the maximization of total structural stiffness as the optimization objectives, the mathematical model is formulated as described in Section 3.1. The initial structural configuration is shown in Fig 19.

**Fig 19:**
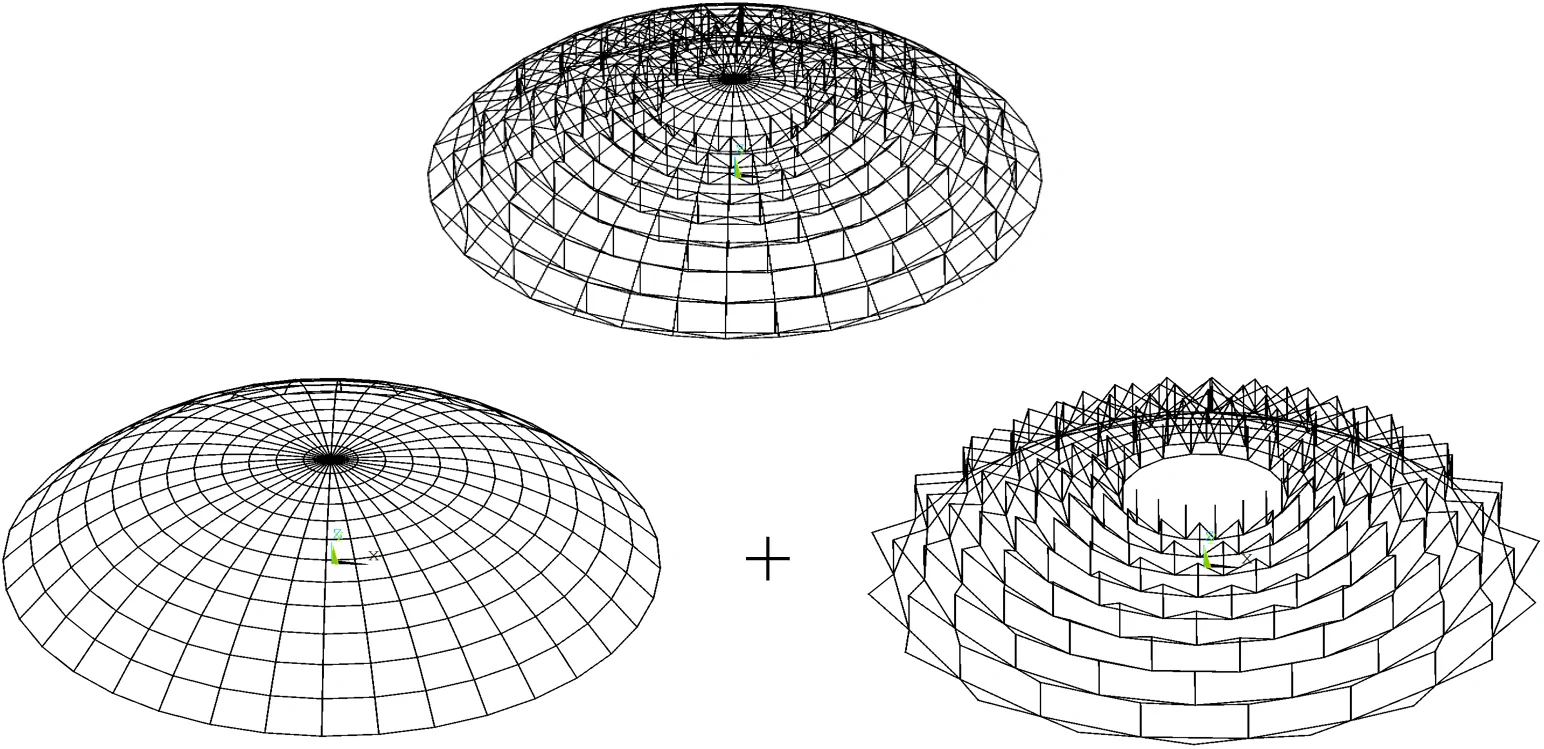
Initial Suspen-Dome model. (span = 110 m, rise = 36 m, ***Nx*** = 36, ***Kn*** = 11).

Figs 20 and 21 illustrate the evolution of the structural configuration and parameter variations of the rib-ring Suspen-Dome during the topology optimization process. After six major iterations, the optimal solution and corresponding configuration were identified. The detailed optimization results are presented in Table 8.

**Table 8:** Topology optimization results of the prefabricated long-span rib-ring Suspen-Dome.

Optimization result	
Span	110m
Rise	21.74m
Rise-to-span ratio	0.198
***Kn***	30
***Nx***	9
Radial interval number	1
Circumferential interval number	1
Cable prestress design values for each ring (kN, from inner to outer)	173.65, 663.39, 859.27
Strut lengths of each ring (m)	6.00, 7.40, 10.43
Strut cross-sectional areas of each ring (cm^2^)	18.12, 77.31, 54.73
Tie lengths of each ring (m)	13.36, 14.41, 15.91
Tie cross-sectional areas of each ring (cm^2^)	89.61, 104.21, 127.17
Total number of members in the reticulated shell	540
Section IDs of circumferential members (from inner to outer)	39,40,40,39,39,41,39,39,39,
Section IDs of radial members (from inner to outer)	39,40,39,39,40,39,40,39,39
Maximum vertical displacement	13.24mm
Total structural mass	176.20
Nonlinear buckling safety factor	3.14
Eigenvalue buckling safety factor	6.68
$q_{input}$	1200

**Fig 20:**
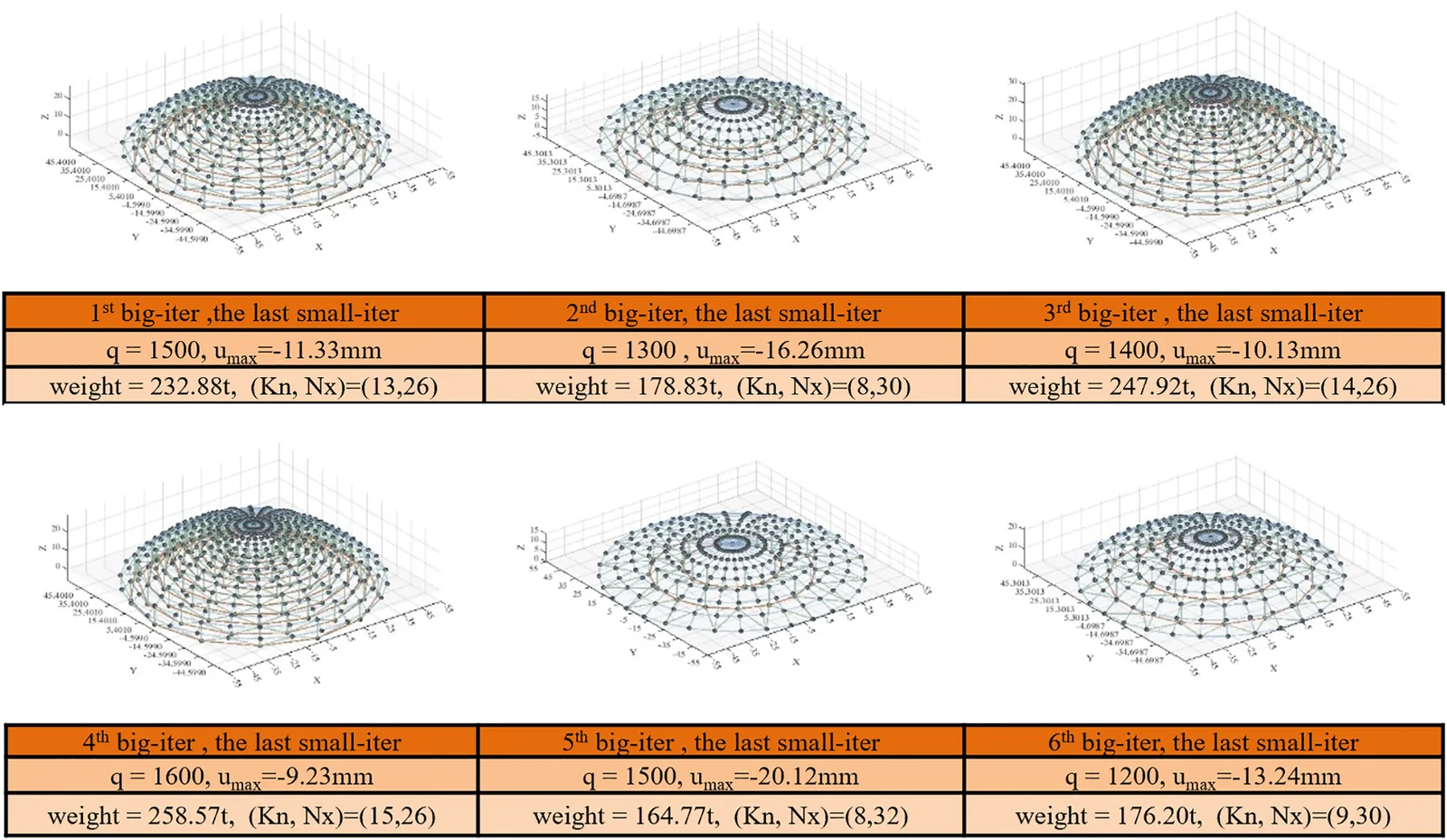
Topology-optimized configuration and parameter variations of the prefabricated long-span rib-ring Suspen-Dome.

**Fig 21:**
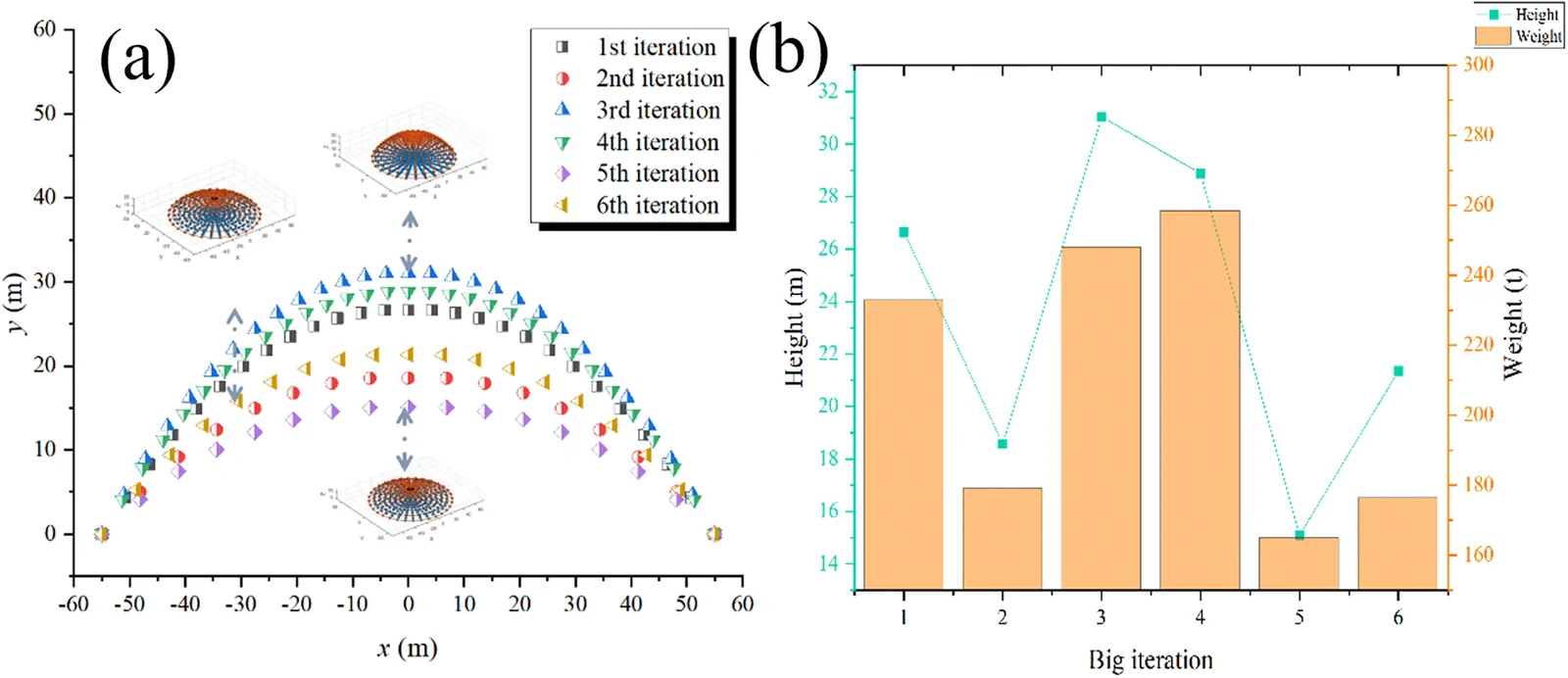
Parameter variations during the topology optimization process. (a) geometric configuration changes in major iterations. (b) variations of rise and weight in major iterations.

It can be seen in Fig 22, for the rib-ring Suspen-Dome structure, because its rib-ring arrangement makes the structural stiffness distribution and force transmission more sensitive to local configuration changes, the stiffness-to-weight ratio and convergence curve exhibit more pronounced stage-wise fluctuations during the optimization process. In particular, when the topological parameters and local member sizes are updated, the structural stiffness first decreases with the lightweight adjustment, and then gradually recovers and stabilizes under the action of prestress correction and pointer-direction adjustment. Compared with the Kiewitt-Lamella structure, the optimization response of the rib-ring structure better reflects the adaptability of the proposed method to topologies with local sensitivity, indicating that this method is not only applicable to Suspen-Dome structures with strong regularity, but can also be used for structural forms that are more significantly affected by local topological features.

**Fig 22:**
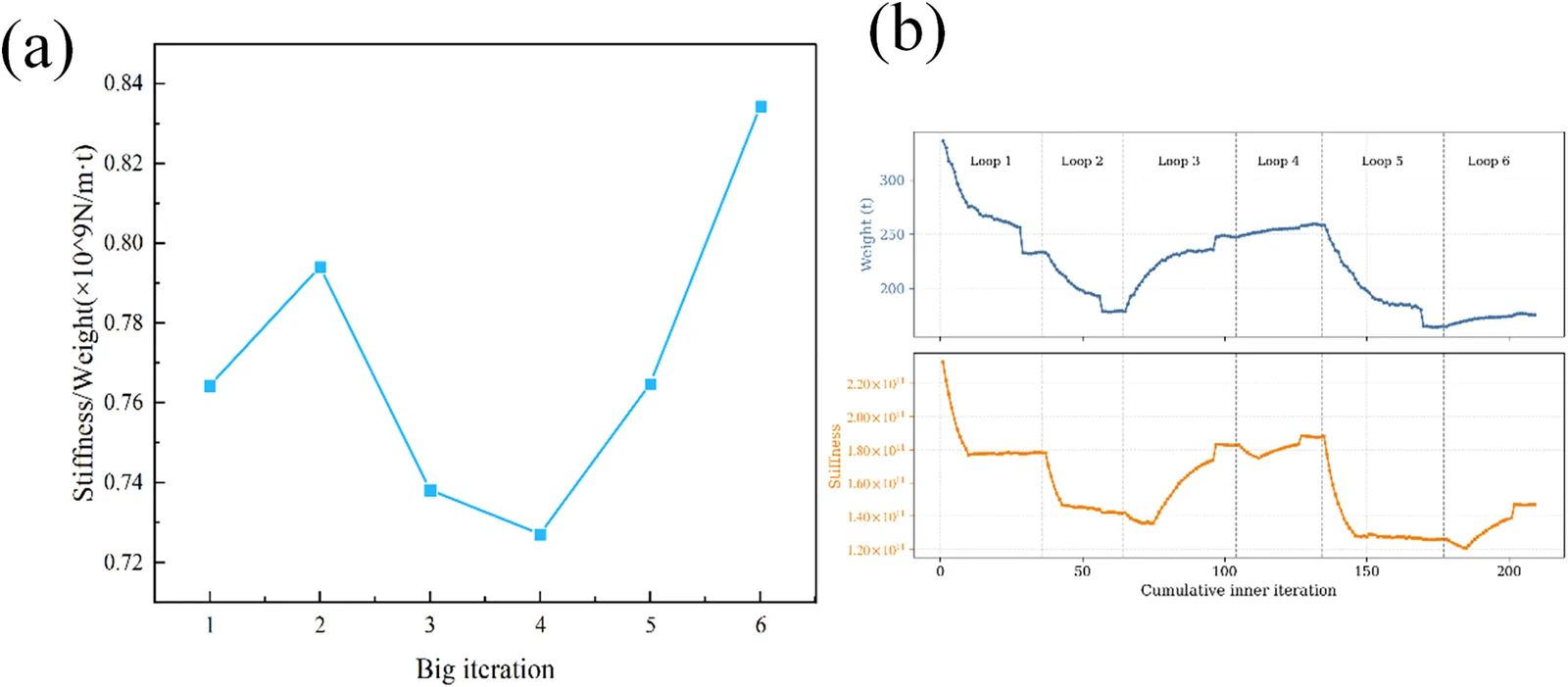
Structural performance index and convergence behavior during optimizatio. (a) Stiffness-to-weight ratio of the structure. (b) Convergence curve.

According to Table 8, the optimal topology results in a total steel consumption of 176.20 t under the given load, corresponding to a unit steel usage of 18.6 kg/m^2^. This represents a substantial saving in steel while ensuring structural safety.

The prestress values of the three circumferential cable rings are 173.65 kN, 663.39 kN, and 859.27 kN, respectively, all within a reasonable range. Under the optimal topology, the maximum vertical displacement is well below the code limit of 250 mm, confirming that the structural stiffness is satisfactory. The eigenvalue buckling factor of 6.68 further demonstrates that the structure can resist relatively high critical loads.

In Fig 23, the histograms of stress-ratio distributions for the reticulated shell members in the optimized schemes exhibit multi-peak characteristics. In the first histogram, a large number of members fall within the 0–0.1 range, while the 0.1–0.9 range is relatively uniform with a noticeable concentration at 0.7–0.9. In the second histogram, stress ratios are mainly distributed in the 0–0.3 and 0.6–0.9 ranges. In the third histogram, stress ratios are concentrated in the 0–0.7 range, with a particularly high proportion in the 0.6–0.7 interval.

**Fig 23:**
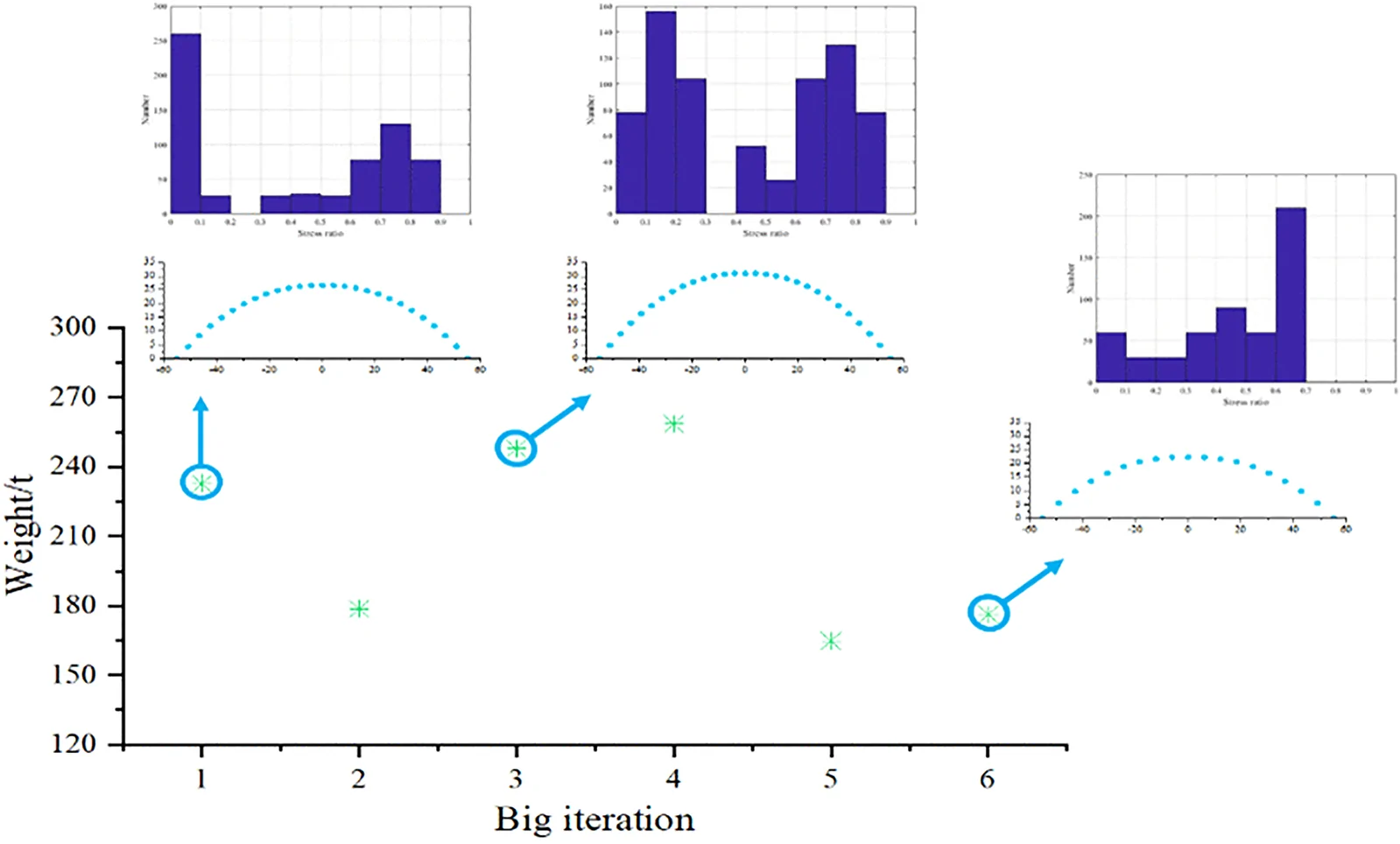
The optimum individuals of every final big iteration.

In line with the design concept of the rib-ring Suspen-Dome, the lower cable-strut system adopts a full-stress design to maximize material utilization, whereas the upper reticulated shell retains an appropriate safety reserve to improve overall stiffness and stability. The third histogram corresponds to the optimal result, where more members fall into the relatively high but non-extreme range of 0.6–0.7, achieving a more uniform stress distribution and avoiding excessive stress concentration. This highlights the advantages of a partial full-stress design, striking a better balance between minimizing weight and maximizing stiffness, and is consistent with the overall optimization philosophy of the rib-ring Suspen-Dome.

For the prefabricated rib-ring Suspen-Dome with a span of 110 m, the optimal rise-to-span ratio under the dual objectives of minimum weight and maximum stiffness is approximately 0.20. The corresponding number of rings and circumferential divisions are preferably 9 and 30, respectively, providing a useful reference for practical structural design and selection.

### 5.3. Static analysis of the optimized structure

The displacement, internal force, and stress contour diagrams of the optimal topology are shown in Figs 24–26. After optimization, none of the structural materials exceed the yield strength, and no stress concentration is observed, indicating that the structure remains in a safe state with adequate safety reserve.

**Fig 24:**
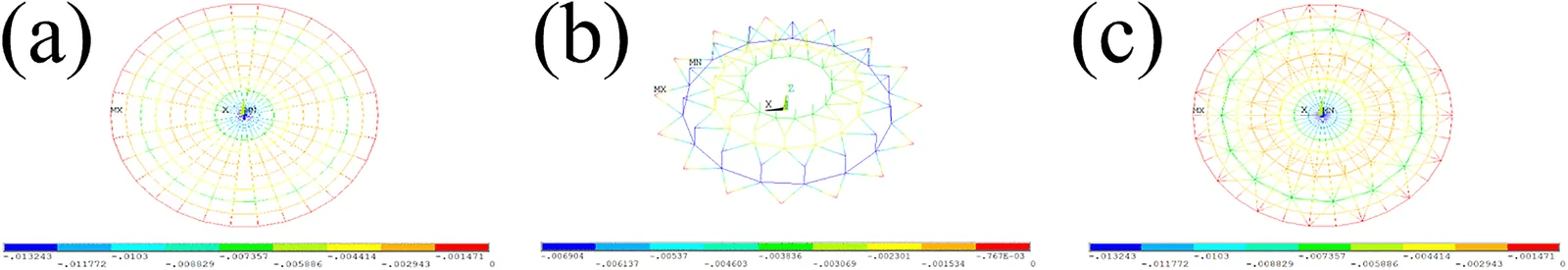
Displacement contours of the Rib-Ring Suspen-Dome. (a) upper reticulated shell. (b) cable-strut system. (c) entire structure.

**Fig 25:**
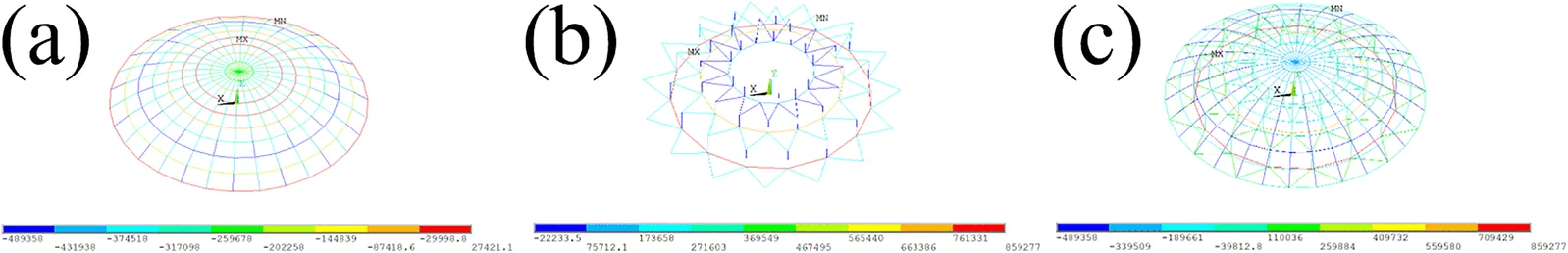
Internal force contours of the Rib-Ring Suspen-Dome. (a) upper reticulated shell. (b) cable-strut system. (c) entire structure.

**Fig 26:**
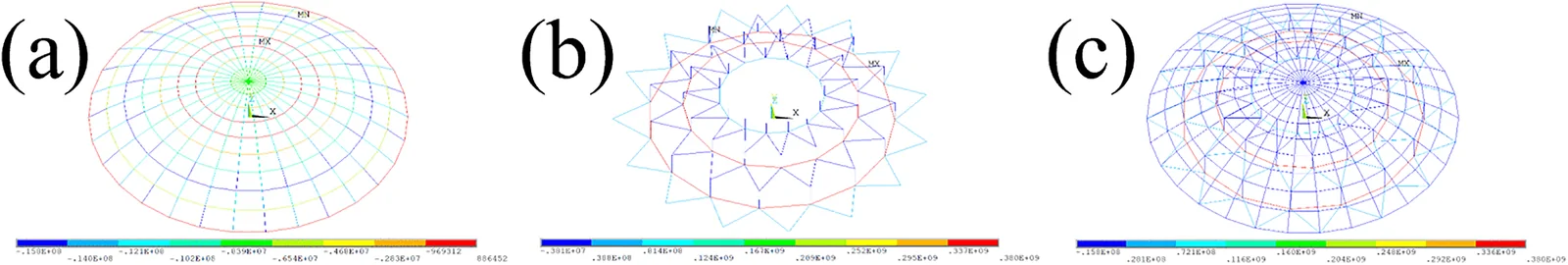
Stress contours of the Rib-Ring Suspen-Dome. (a) upper reticulated shell. (b) cable-strut system. (c) entire structure.

From the displacement contours, the maximum vertical displacement of the reticulated shell is 13.24 mm, which is far below the limit of 275 mm, demonstrating favorable stiffness performance. From the stress contours, the stress distribution of the reticulated shell is uniform, without stress concentration; most circumferential and radial members are in compression. This indicates that under the SMM, the rib-ring reticulated shell members mainly carry compressive forces.

The cable-strut system exhibits small displacements without unfavorable deformations. Its stress state does not exceed the tensile limit, and the reinforcement strategy is consistent with the prestress design values. Overall, the optimized structure is safe, maintains normal service functionality, demonstrates rational load transfer, and achieves efficient material utilization.

### 5.4. Modal analysis of the optimized structure

As shown in Fig 27, a modal analysis of the rib-ring Suspen-Dome was conducted for the first six modes, with frequencies ranging from 1.46 Hz to 3.31 Hz. The first and second modes correspond to vertical and horizontal symmetric vibrations, while the third through sixth modes manifest as global instabilities. The maximum deformations occur between the sixth and eighth rings, mainly in the form of torsional deformation modes, indicating that the structure is susceptible to torsional failure of the reticulated shell.

**Fig 27:**
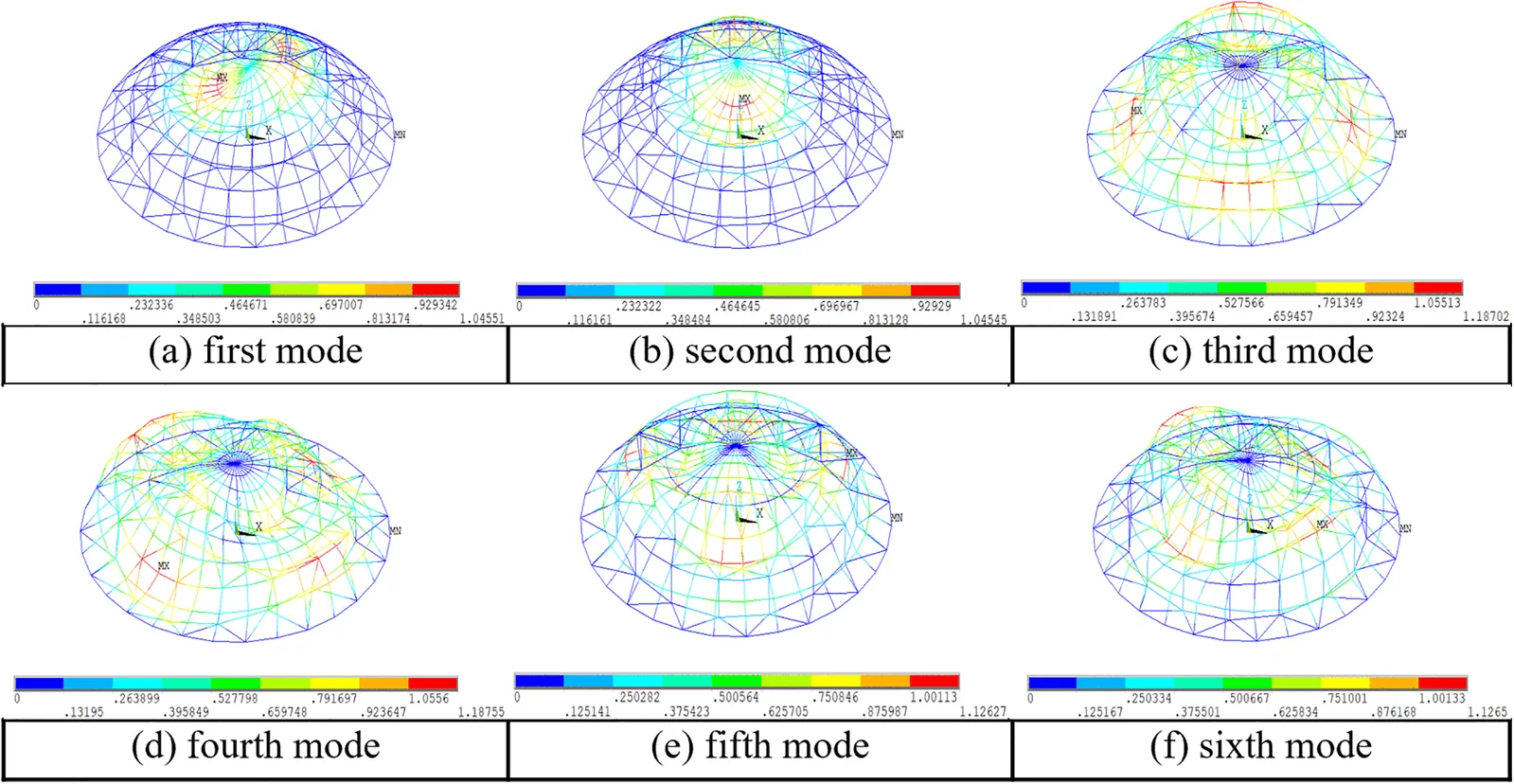
First six vibration modes of the Rib-Ring Suspen-Dome. (a) first mode; (b) second mode; (c) third mode; (d) fourth mode; (e) fifth mode; (f) sixth mode.

According to Table 9, the buckling factors (structural buckling eigenvalues) are relatively large, suggesting that the structure is not prone to buckling failure and thus exhibits superior stability. In comparison with the Kiewitt–Lamella Suspen-Dome, the rib-ring dome shows smaller buckling factors, implying relatively lower stability. The first six natural frequencies lie within the range of 1.46 Hz to 3.31 Hz, indicating that the structure possesses a certain degree of flexibility. This endows it with favorable deformation coordination capacity and energy dissipation characteristics under seismic and wind excitations, thereby enhancing its overall ductility and delaying failure.

**Table 9:** First six modal parameters of the prefabricated long-span Rib-Ring Suspen-Dome.

Modal order	Eigenvalue	Frequency
1	84.06	1.46
2	84.06	1.46
3	376.56	3.09
4	376.56	3.09
5	432.09	3.31
6	432.09	3.31

As shown in Fig 28, the unit-mass eigenvalues of the Kiewitt–Lamella Suspen-Dome in the first to sixth modes are generally higher than those of the rib-ring Suspen-Dome, indicating superior overall stiffness and stability. In addition, the eigenvalue distribution of the Kiewitt–Lamella Suspen-Dome across modes is relatively smooth without abrupt fluctuations, suggesting a more uniform stiffness distribution and a lower likelihood of local weak zones. Therefore, the Kiewitt–Lamella configuration demonstrates a more efficient balance between weight reduction and global stability, making it a preferable choice for long-span Suspen-Dome design.

**Fig 28:**
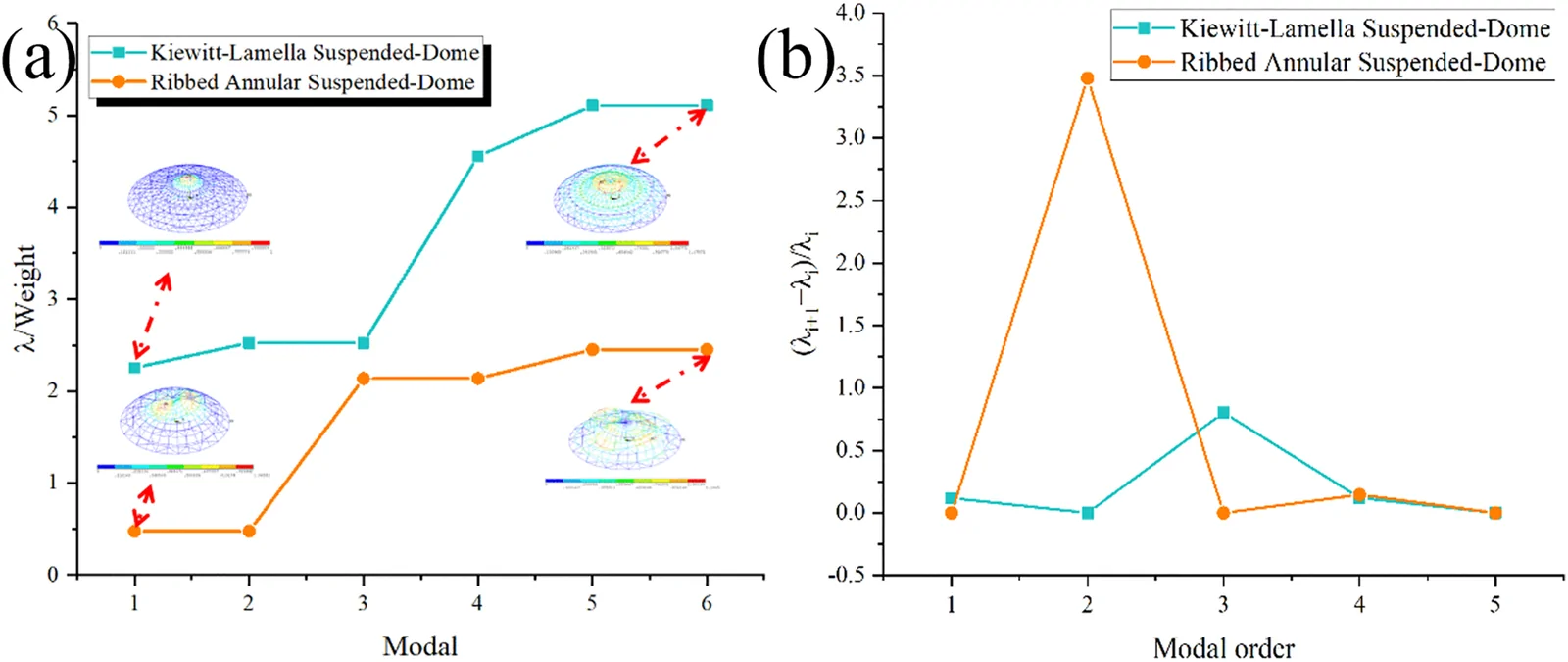
Comparison of optimization effectiveness between the two Suspen-Dome structures. (a) eigenvalue-to-weight ratio; (b) variation rate of eigenvalues during topology optimization.

## 6. Sensitivity,robustness and comparative studies

The 100 m-span Kiewitt–Lamella Suspen-Dome described in Section 4 was selected as the representative example in this section. Except for the investigated load level or random seed, all other initial conditions, design-variable ranges, constraint conditions, and algorithm parameters were kept consistent with those adopted in Section 4. The sensitivity of the final optimization results and the robustness of the stochastic search process were investigated through optimizations under different load levels and repeated optimizations using different random seeds, respectively.

### 6.1. Sensitivity analysis of optimization results under different load levels

To investigate the influence of load-level variations on the optimization results, the 100 m-span Kiewitt–Lamella Suspen-Dome described in Section 4 was optimized under load levels equal to 1.0, 1.2, and 1.4 times the baseline load. Except for the load level, the design-variable ranges, search step sizes, constraint conditions, algorithm parameters, and termination conditions were kept identical for all cases. The same criterion was adopted to select the representative Pareto solution for each case, and the optimization results are presented in Table 10.

**Table 10:** Sensitivity analysis of optimization results under different load levels.

Indicator	Group 1	Group 2	Group 3
Structural weight/t	271.56	299.77	301.10
Maximum vertical displacement/mm	20.90	45.56	34.47
*Kn*	11	15	11
*Nx*	12	10	14
*q*	2000	2200	1800
Interval	1,3	4,1	3,2

As shown in Table 10, when the load level was increased to 1.2 and 1.4 times the baseline value, the optimized structural weight increased by 10.39% and 10.88%, respectively, indicating that the structural weight of the optimized solutions was relatively insensitive to load variations within the investigated range. By contrast, the maximum vertical displacement increased by 118.00% and 64.93%, respectively, demonstrating that the structural deformation index was more sensitive to the load level and the resulting optimized topological configuration. As the load level varied, the algorithm correspondingly adjusted the number of rings, the number of radial divisions, the initial force-density parameter, and the cable-strut arrangement intervals. Feasible solutions satisfying all constraint conditions were obtained under all three load cases, indicating that the proposed method can accommodate topology and size optimization requirements under different load levels. It should be noted that a complete optimization was independently performed for each load case. Therefore, the results in the table reflect the influence of the load level on the final optimized solutions rather than the direct responses of a fixed structure subjected to different loads.

### 6.2. Robustness analysis

To evaluate the influence of the stochastic search process on the optimization results, three independent optimization runs were performed using different random seeds under identical initial parameters and constraint conditions. The same criterion was adopted to select the representative Pareto solution in each run, and the mean, standard deviation, and coefficient of variation of the structural weight, maximum vertical displacement, buckling factors, and number of iterations were calculated. The detailed results are presented in Table 11. The results show that the coefficients of variation of the main performance indicators were all within 10%, indicating that the optimization results obtained from different stochastic search processes exhibited good consistency.

**Table 11:** Statistical results of the optimization under different random runs.

Indicator	Group 1	Group 2	Group 3
Structural weight/t	271.56	285.38	274.87
Maximum vertical displacement/mm	20.90	28.62	26.45
*Kn*	11	11	11
*Nx*	12	12	12
Strut length	6,6,6,7	4.5,5,6,7	5,5,6,7
*q*	2000	2200	2100
Interval	1,3	1,3	1,3
Section IDs of circumferential members (from inner to outer)	39,40,40,43,43,41,39,41,41,41,39	34,39,44,44,44,40,38,44,40,34,39	44,34,39,40,44,38,40,40,39,40,40
Section IDs of radial members (from inner to outer)	41,40,37,40,43,39,40,40,40,37,43	38,39,44,34,34,39,40,40,40,44,40,38	34,44,40,40,38,38,44,40,44,38,49

As shown in Table 11, all three independent runs produced the same overall topological parameters, namely, *Kn* = 11and *Nx* = 12, while the radial and circumferential arrangement intervals were both 1 and 3. This indicates that the proposed algorithm exhibited good consistency in identifying the overall topological configuration of the Suspen-Dome. The mean structural weight obtained from the three runs was 277.27 t, with a coefficient of variation of only 2.60%. The coefficient of variation of *q* was 4.76%, indicating that the structural weight and overall prestress-related parameters were only slightly affected by the stochastic search path. The mean maximum vertical displacement was 25.32 mm, with a coefficient of variation of 15.72%, showing greater variation than the structural weight.Meanwhile, differences were also observed in the strut lengths of individual rings and the section indices of the reticulated-shell members. This indicates that, even with the same overall topology, the algorithm can obtain multiple feasible solutions with similar structural weights but different local section configurations and deformation performances. This result reflects the trade-off between structural weight and deformation performance in multi-objective optimization. Although the local size variables and displacement results exhibited some variation, all solutions obtained from the three runs satisfied the structural constraints, while the overall topology and structural weight remained highly consistent, demonstrating that the proposed method possesses a certain degree of robustness against stochastic search variations.

### 6.3. Comparison between sequential and integrated optimization strategies

To provide direct comparative evidence for the methodological significance of treating the upper and lower structural systems within a unified optimization process, the 100 m-span Kiewitt–Lamella Suspen-Dome presented in Section 4 was selected for a controlled comparison between sequential and integrated optimization strategies. The purpose of this comparison is to examine whether allowing the two mechanically coupled subsystems to participate simultaneously in the optimization changes the attainable design solutions relative to conventional sequential treatment.

In the sequential strategy, the upper reticulated shell was optimized first. During this stage, only the upper-system variables were included in the optimization search. The design rules of the lower cable–strut system were fixed; however, its nodal connectivity and geometric parameters were regenerated according to the current upper-shell configuration, and the corresponding prestress equilibrium state was recalculated using the same procedure described in Section 3.2.1. These quantities were therefore treated as dependent structural quantities rather than optimization variables. After the representative upper-shell solution was obtained, its topology, geometry, and member sections were fixed, and the lower cable–strut system was subsequently optimized.

In the integrated strategy, the upper- and lower-system variables were optimized within the same optimization process, as described in Section 3.3. The two strategies employed identical initial structural conditions, material properties, loading conditions, objective functions, structural constraints, design-variable bounds, finite-element models, and algorithmic parameters. Therefore, the comparison isolates, as far as practicable, the effect of sequential versus integrated treatment of the two mechanically coupled subsystems, rather than differences caused by the underlying optimization algorithm or structural analysis model.

As shown in Table 12, the integrated and sequential optimization strategies produced different final structural configurations and performance characteristics. The integrated strategy reduced the structural mass from 303.50 t to 271.56 t, corresponding to a reduction of 10.52%, while the maximum vertical displacement decreased from 30.06 mm to 20.90 mm. The resulting topological parameters also differed, with Kn changing from 13 to 11 and the cable–strut arrangement interval changing from 1,1–1,3. Although the nonlinear buckling factor of the integrated solution was slightly lower than that of the sequential solution (3.32 versus 3.79), both solutions satisfied the prescribed stability requirements. These results indicate that the integrated treatment enables the topology, size, and prestress-related variables of the upper and lower subsystems to interact during the optimization process, leading to a different and more lightweight design solution than that obtained through sequential optimization.

**Table 12:** Comparison between sequential and integrated optimization strategies.

Indicator	Integrated optimization	Sequential optimization
Structural weight/t	271.56	303.50
Maximum vertical displacement/mm	20.90	30.06
*Kn*	11	13
*Nx*	12	12
Nonlinear buckling factor	3.32	3.79
*q*	2000	1850
Interval	1,3	1,1

## 7. Discussion

The surrogate-assisted multi-objective topology optimization method proposed in this study enables the coordinated optimization of the upper reticulated shell and lower cable-strut system of prefabricated long-span Suspen-Dome structures within a unified framework. The controlled comparison in Section 6.3 provides direct evidence of the effect of treating the upper reticulated shell and lower cable–strut system within the same optimization process. Although the sequential and integrated strategies employ the same structural model, objective functions, constraints, and underlying optimization algorithm, they produce different solution distributions and final structural configurations. This indicates that the contribution of the integrated treatment is not simply an increase in the number of design variables; rather, it allows the topology, member sizing, and prestress-related parameters of the two mechanically coupled subsystems to interact during the search process. Nevertheless, the proposed method still has certain limitations. The reliability of the MLP screening model depends on the size of the training dataset and the coverage of the parameter space, and its generalization capability for new spans, topologies, or loading conditions outside the training distribution requires further verification. The present study mainly considers static performance and stability constraints, while seismic effects, wind-induced vibration, construction stages, and long-term service effects have not yet been incorporated. In addition, PFSD is a design strategy developed specifically according to the mechanical characteristics of Suspen-Dome structures, and its applicability to other spatial structural systems requires further validation through additional engineering examples and comparative studies. Future research will further expand the training dataset and structural types and incorporate dynamic performance, construction processes, and life-cycle indicators into the optimization framework.

## 8. Conclusions

This study proposes a multi-objective topology optimization design method for prefabricated long-span Suspen-Dome structures based on the SMM-iPGSA approach. The main conclusions are as follows:

1. Taking prefabricated long-span Suspen-Dome as examples, the optimization simultaneously considers the upper reticulated shell (size, geometry, and topology) and the lower cable-strut system (topology, strut/tie sizes, and cable prestress) under global stability constraints. This integrated design not only significantly improves structural performance and economic efficiency while ensuring safety, but also achieves a rational prestress distribution that can be directly applied in engineering practice.
2. An SMM-iPGSA-based optimization framework was extended to the coupled topology–size–prestress design of Suspen-Dome structures. By allowing the topology, geometry, and member sections of the upper reticulated shell to be optimized together with the layout, member sizing, and cable prestress of the lower cable–strut system, the method enables the two mechanically coupled subsystems to interact within the same search process. The controlled comparison with sequential optimization further confirms that this coupled treatment leads to different structural configurations and performance outcomes under otherwise identical optimization conditions.
3. A new method for determining cable forces is introduced by combining the tension compensation method with the elastic support method. The elastic support method is first used to control cable forces within the vicinity of the target values, followed by iterative refinement using the tension compensation method to achieve more accurate prestress values. This hybrid approach addresses the issue of overestimated prestress levels inherent in the elastic support method and reduces the excessive dependence on initial strain assumptions in the tension compensation method.
4. The MLP-based screening model provides an effective pre-filtering tool for reducing repeated ANSYS analyses. It achieved an accuracy of 92.12%, a recall of 95.81%, and an F1-score of 95.24% on the testing set. Since all retained candidates are still verified by ANSYS, the reliability of the final optimization results is not compromised.
5. For the 100 m-span Kiewitt–Lamella Suspen-Dome, the structural mass was reduced from 1,380.00 t to 271.56 t, corresponding to a reduction of 80.32%. The maximum vertical displacement was 20.90 mm, while the nonlinear buckling factor and eigenvalue buckling factor were 3.32 and 8.15, respectively. For the 110 m-span rib-ring Suspen-Dome, the structural mass was reduced from 336.00 t to 176.20 t, corresponding to a reduction of 47.56%. The maximum vertical displacement was 13.24 mm, while the nonlinear buckling factor and eigenvalue buckling factor were 3.14 and 6.68, respectively. These results demonstrate that the optimized schemes substantially reduced steel consumption while maintaining relatively small vertical deformations and sufficient overall stability reserves, thereby validating the capability of the proposed method to coordinate structural lightweighting, deformation control, and stability performance.
6. The controlled comparison with sequential optimization showed that the integrated strategy reduced the structural mass from 303.50 t to 271.56 t (10.52%) and the maximum vertical displacement from 30.06 mm to 20.90 mm, while producing different topological parameters. Although the nonlinear buckling factor decreased from 3.79 to 3.32, both solutions satisfied the prescribed stability requirements. The comparison confirms that the coupled treatment of topology, size, and prestress-related variables has a direct influence on the final structural design.

## Supporting information

S1 File Data. (CSV)
