# Supplementary material for: Surrogate-assisted multi-objective topology optimization design method for prefabricated large-span Suspen-Dome based on structural-mapping-method and improved-PGSA
Source: PLoS One. 2026 Nov 2;21(11):e0361862. doi: 10.1371/journal.pone.0361862 (PMC13726994; doi:10.1371/journal.pone.0361862)
Supplement: Supplementary file 1 [file pone.0361862.s003.docx]

Reviewer #1: This manuscript focuses on investigating the integrated optimization of prefabricated large-span Suspen-Dome structures by simultaneously considering the size, geometric configuration, and topology of the upper reticulated shell, together with the topology and cable forces of the lower cable-strut system. To ensure practical applicability, the design variables are classified into topological and size variables. The former define the structural configuration and connectivity, while the latter determine material-related properties. The optimization aims to maximize stiffness and minimize weight, subject to member slenderness, material strength, local stability, and overall structural stability constraints. An multilayer perceptron (MLP)-based candidate screening model is introduced to accelerate the optimization algorithm and reduce unnecessary ANSYS analyses. A partial fully stressed design (PFSD) concept is proposed, in which the reticulated shell serves as a structural safety reserve. The proposed structural mapping method-improved plant growth algorithm (SMM-iPGSA) is validated through two examples. Results show that the optimized structures exhibit improved stability, more uniform stress distribution, and displacements well below the allowable limits.
The manuscript describes a technically sound piece of scientific research with data that supports the conclusions. Experiments have been conducted rigorously, with appropriate controls, replication, and sample sizes. The conclusions are drawn appropriately based on the data presented. The statistical analysis has been performed appropriately and rigorously. The manuscript is presented in an intelligible fashion and written in Standard English. Therefore, the evaluation of this manuscript is generally favorable and positive. However, there are still some issues that need to be carefully addressed, as listed below:

**Q1. For the problem addressed in this manuscript, what is the advantage of the proposed method compared with other existing similar approaches? A balanced discussion of both advantages and limitations will help clarify the contribution and novelty of the proposed method.**

**Author’s Response:**

Thank you for this valuable comment. We agree that the original manuscript did not sufficiently discuss the advantages of the proposed method over existing approaches or clearly define its range of applicability. In response, a more balanced discussion of the advantages and limitations of the proposed method has been added to the revised manuscript.

Specifically, the main advantages of the proposed method are as follows: the topology, size, and prestress variables of both the upper reticulated shell and the lower cable–strut system are simultaneously considered within a unified optimization framework; the structural mapping method enables coordinated representation of different types of design variables; the MLP-based candidate screening model reduces unnecessary finite element analyses; and the PFSD strategy coordinates structural safety reserve with material utilization efficiency. Compared with existing methods that optimize only an individual subsystem or adopt a fixed topology, the proposed method provides a broader design space and improved applicability to engineering design.

At the same time, the revised manuscript explicitly clarifies the limitations of the proposed method. Its performance depends on the coverage of the training samples, while dynamic response, construction stages, and long-term service performance have not yet been considered. In addition, the applicability of the PFSD strategy to other types of spatial structures requires further validation. These additions provide a more objective definition of the contributions, applicability, and limitations of the proposed method, as well as directions for future research.

**Q2. The introduction section needs to be further strengthened. Specifically, topology optimization, as the most comprehensive category of structural optimization and a powerful design tool, covers a wide range of application fields. There are many commonly-used methods for structural topology optimization in recent times, such as the enhanced proportional topology optimization method (https://doi.org/10.1080/15397734.2026.2661100), the parameterized level set method (https://doi.org/10.1080/15397734.2025.2571736), and the asymptotic concentration method (https://doi.org/10.3934/era.2023196). If possible, it is recommended to briefly comment on them to enrich the scalability of this manuscript.**

**Author’s Response:**

Thank you for this valuable suggestion. We agree that topology optimization is an important and widely applicable design approach in the field of structural optimization, and that an appropriate discussion of representative recent developments can provide a more comprehensive research background and better clarify the positioning of the present study.

Following the reviewer’s suggestion, we have expanded the Introduction by adding a brief review of recent developments in structural topology optimization. The recommended studies on the enhanced proportional topology optimization method, the parameterized level-set method, and the asymptotic concentration method have been incorporated into the revised manuscript. Their main characteristics and applications in structural topology optimization are briefly discussed, together with their relationship to and differences from the optimization framework adopted in the present study.

These revisions provide a broader overview of recent developments in structural topology optimization and further clarify the positioning and potential extension of the present study in the coordinated topology and size optimization of large-span Suspen-Dome structures. The corresponding references have also been added to the revised manuscript.

(Pages 2,lines 49-63; Pages 36, lines 957-965)

**Q3. Most of the images in the manuscript have low resolution and remain blurry even when enlarged, which makes it difficult to see their content clearly. Especially the images in Figures 3, 6, 8, 10, 11, 12, 14, 15, 16, 17, 19, 20, 21, 23, 24, 25, 26, 27. If applicable, it is recommended to carefully make appropriate modifications to the similar issues together.**

**Author’s Response:**

Thank you for this careful comment. In response, we re-examined all figures throughout the manuscript, with particular attention to Figs. 3, 6, 8, 10–12, 14–17, 19–21, and 23–27, as specifically identified by the reviewer. For figures that could be regenerated or re-exported, higher-resolution versions were produced, and the sizes of the text, axis labels, legends, and other annotations were appropriately increased to improve their readability both at the normal display size and when enlarged.

For some finite element contour plots, the figures contain relatively dense information, including the overall structural model, contour distributions, color scales, and numerical values. For these figures, the output resolution, figure dimensions, display range, and annotation sizes were readjusted as far as practicable without altering the original computational results or contour information, thereby improving their clarity and readability. In addition, the other figures in the manuscript were also carefully checked, and those with similar issues were revised accordingly. All revised figures have been updated in the manuscript.

(Pages 7,lines 221; Pages 12,lines 361; Pages 14,lines 409; Pages 15, lines 464; Pages 18,19,lines 567; Pages 24, lines 685-687; Pages 30,lines 777-780)

**Q4. If possible, it is recommended to change the color of the images in Tables 4 and 6, so as to enhance content recognition and readability.**

**Author’s Response:**

Thank you for this valuable suggestion. In response, the images in Tables 4 and 6 were carefully re-examined and revised. Each table contains three structural diagrams showing the complete structure, the upper reticulated shell, and the lower cable–strut system, respectively. For the separate diagrams of the upper reticulated shell and the lower cable–strut system, clearer and more distinguishable colors were adopted to improve the identification of different structural components and enhance the overall readability of the figures.

For the complete structural diagrams, we also tested the use of different colors to distinguish the upper reticulated shell from the lower cable–strut system, together with increased line widths. However, because the complete structures contain a large number of members and considerable overlap occurs between the upper and lower components in the projected view, the use of multiple colors and thicker lines introduced additional visual congestion and made the overall topology and member connectivity less clear than in the original monochromatic representation. Therefore, after comparison, the original monochromatic presentation was retained for the complete structural diagrams in Tables 4 and 6, while the separate diagrams of the upper reticulated shell and the lower cable–strut system were revised using clearer colors. The corresponding figures have been updated in the revised manuscript.

(Pages 21-22; Pages 27-28)

**Q5. The PLOS Data policy requires authors to make all data underlying the findings described in their manuscript fully available without restriction, with rare exception (please refer to the Data Availability Statement in the manuscript PDF file). The data should be provided as part of the manuscript or its supporting information, or deposited to a public repository. Although some data are provided in the manuscript, it does not seem to meet the requirements of the PLOS Data policy. Therefore, it is suggested that the authors make appropriate modifications based on the specific circumstances.**

**Author’s Response:**

Thank you for this valuable comment. We agree that the accessibility of the data associated with this study should be further improved in accordance with the PLOS Data Policy. In response, we organized the data used for the machine-learning component of the proposed method and added a CSV data file as Supporting Information to the revised manuscript.

The CSV file contains the dataset used to train the MLP-based candidate screening model, including the corresponding structural design parameters, statistical features of the member sections, and sample feasibility labels. This dataset was used for the training and validation of the MLP screening model adopted in the optimization process to reduce unnecessary finite element analyses. Providing this dataset enables readers to more clearly understand the source and input format of the MLP training data and facilitates further verification of the associated training and screening process.

The **Data Availability Statement** has also been revised accordingly to specify that the CSV dataset is provided as Supporting Information with the revised manuscript. These revisions further improve the transparency and reproducibility of the data associated with the MLP model training.

**Q6. PLOS ONE does not copyedit accepted manuscripts, so the language in submitted articles must be clear, correct, and unambiguous. Any typographical or grammatical errors should be corrected at revision, so the specific errors are noted as follows.**

(1) When abbreviations first appear, they should be accompanied by their corresponding full names. This is a writing principle. However, in the abstract (line 18) and main text (line 120), the abbreviation “MLP” violates this principle. Moreover, the abbreviation “GSI&PS-PGSA” (line 203) violates this principle. If applicable, it is recommended to carefully make appropriate modifications to the similar issues together.

(2) The citations in the text should be accurate. This is also a writing principle. However, both "Kaveh [8]" (line 42) and "Adam, by contrast, considered minimizing member stresses and maximizing overall structural stiffness [31]" (lines 95-96) violate this principle, as both References [8] and [31] have more than one contributor. If applicable, it is recommended to carefully make appropriate modifications to the similar issues together.

(3) The typographical errors in the text should be eliminated. This is also a writing principle. However, "Examples 1" (line 199) violates this principle, and its correct form should be 'Example 1'. If applicable, it is recommended to carefully make appropriate modifications to the similar issues together.

(4) All Figures should be mentioned in the main text. This is also a writing principle. However, Figure 1 was not mentioned in the main text, which violates this principle. If applicable, it is recommended to carefully make appropriate modifications to the similar issues together.

**Author’s Response:**

Thank you for these careful comments. In response, we systematically checked the entire manuscript with particular attention to language expression, abbreviation definitions, citation style, typographical consistency, and figure citations, and the relevant issues have been revised throughout.

Specifically, (1) the full terms have now been provided when abbreviations first appear in the abstract and main text, including MLP and GSI&PS-PGSA, and other abbreviations throughout the manuscript were also checked for consistent first-use definitions; (2) the author citation style in the main text was reviewed, and citations involving references with multiple authors were revised to the appropriate form, together with a check of other similar cases; (3) typographical and grammatical errors such as “Examples 1” were corrected, and the manuscript was further checked for similar language and formatting issues; and (4) the first citation of Fig. 1 was added to the main text, and all other figures were reviewed to ensure that each figure is appropriately referred to in the manuscript.

These revisions have been incorporated into the revised manuscript to further improve the accuracy of the language, the consistency of the formatting, and the overall readability of the paper.

(1): (Pages1 ,lines18,19-20, 43; Pages 4, lines 134; Pages 7, lines223-224)

(2): (Pages1 ,lines 37,41,42; Pages 2 ,lines 63,65; Pages 3, lines95,109)

(3): (Pages6 ,lines 220)

**Q7. If possible, it is recommended to carefully check and revise the entire manuscript, strive to eliminate any ambiguities and unreadable points, and thus improve the presentation of the manuscript.**

**Author’s Response:**

Thank you for this valuable suggestion. In response, we conducted a further systematic review and language revision of the entire manuscript, with particular attention to sentences that could be ambiguous, unclear, or difficult to understand. Relevant expressions were reorganized and revised where necessary. Several overly long sentences or sentences with unclear logical relationships were also divided or rewritten, and the terminology, abbreviations, symbols, and related expressions were further standardized throughout the manuscript. In addition, grammatical, spelling, and formatting issues were carefully rechecked and corrected.

These revisions have further improved the clarity, logical coherence, and readability of the manuscript, and the corresponding changes have been incorporated into the revised version.

**Reviewer #2: The manuscript presents a surrogate-assisted multi-objective topology optimization framework for prefabricated Suspen-Dome structures by integrating a structural mapping method (SMM), an improved plant growth simulation algorithm (iPGSA), a neural-network-based screening strategy, and a partial fully stressed design concept. Although the topic is relevant to structural optimization, the manuscript suffers from substantial methodological and scientific deficiencies. The claimed novelty is not sufficiently demonstrated, and many important methodological details are missing or lack rigorous validation. Therefore, I do not recommend publication in its current form.**

Major comments:
**Q1. The claimed methodological novelty is weak. The work mainly integrates existing methods rather than proposing a fundamentally new optimization methodology.**

**Author’s Response:**

Thank you for this valuable comment. We agree that the original manuscript did not sufficiently clarify the hierarchy and boundaries of the methodological contributions, which may have made the structural mapping method, iPGSA, the pointer adjustment method, and the auxiliary computational modules appear to be a simple combination of existing techniques. However, the present study does not merely place these existing methods side by side.

The structural mapping method and iPGSA constitute the basic solution framework of this study, while the random parallel search and optimal-value elimination mechanisms in iPGSA were developed in the authors’ previous work. The main methodological contribution of the present study lies in adapting the bidirectional evolutionary concept of BESO to the coupled discrete topology–size–prestress optimization problem of Suspen-Dome structures and transforming it into a pointer adjustment mechanism suitable for discrete structural parameters. Conventional BESO generally adds or removes finite elements according to element sensitivities. In contrast, the present method does not directly add or remove elements. Instead, the number of rings, number of radial divisions, arrangement intervals of the cable–strut system, and member section parameters are taken as the adjustment variables. The direction in which the design variables should increase or decrease in the next iteration is determined according to the variation relationship between structural mass and a comprehensive axial-stiffness index in adjacent iterations.

Therefore, the contribution of this study is not to re-propose SMM or iPGSA themselves, but to extend the existing optimization framework specifically for the discrete coupled design problem of Suspen-Dome structures. This extension enables the topology, geometry, and section parameters of the upper reticulated shell to be optimized simultaneously with the topology, size, and prestress parameters of the lower cable–strut system, rather than optimizing the two subsystems separately. In addition, the elastic-support–tension-compensation iterative method proposed in this study is used to improve the accuracy of cable prestress determination, while the MLP model serves as a candidate pre-screening tool to reduce unnecessary finite element analyses.

Following the reviewer’s comment, we revised the statements regarding novelty in the abstract, Introduction, and Conclusions. The revised manuscript no longer describes the entire SMM-iPGSA framework as a completely new fundamental optimization algorithm. Instead, the contribution of this study is more accurately positioned as a methodological extension for the coupled discrete topology–size–prestress optimization of Suspen-Dome structures, including the pointer adjustment mechanism and its new application to integrated collaborative optimization. We believe that the variable representation, directional guidance, and coupled solution mechanisms developed for this specific structural system constitute a problem-driven methodological innovation rather than a simple assembly of existing methods.

**Q2. The mathematical formulation is incomplete and inconsistent. The multi-objective formulation is mathematically unclear and the definition of structural stiffness is questionable.**

**Author’s Response:**

Thank you for this valuable comment. We agree that simply expressing the two objectives in the original manuscript as “minf_1_+maxf_2_” does not accurately represent a Pareto multi-objective optimization problem. In addition, directly referring to the summation of EA/L over all members as “structural stiffness” could lead readers to interpret it as a rigorous measure of the overall structural stiffness derived from the global stiffness matrix or the load–displacement relationship. Therefore, the original terminology was not sufficiently precise.

In response, the mathematical formulations in Sections 3.1.1 and 3.1.4 have been reorganized in the revised manuscript. First, structural mass and the aggregate axial stiffness index are converted into two dimensionless minimization objectives as follows:

$$minF(x)=[\begin{matrix} f_{1}(x) \\ f_{2}(x) \end{matrix}]=[\begin{matrix} \frac{M(x)}{M_{0}} \\ \frac{K_{0}}{K(x)} \end{matrix}]$$

where

$$M\left( x \right)=\sum_{e}^{N_{e}} \rho\cdot A(x)\cdot L(x)$$

and

$$K\left( x \right)=\sum_{e}^{N_{e}} \frac{E\cdot A(x)}{L(x)}$$

The term “structural stiffness” has been consistently replaced by “aggregate axial stiffness index” throughout the revised manuscript. This index is no longer interpreted as the rigorous overall stiffness of the structure, but is used only to compare the overall level of member axial stiffness among different candidate designs. The actual global deformation and stability performance of the structure are still constrained and verified through the maximum vertical displacement, member strength, slenderness ratio, compression–bending stability, eigenvalue buckling factor, and nonlinear buckling factor.

Second, the design variables have been reorganized into topological variables, geometric variables, section variables, and prestress and form-finding variables. The corresponding upper and lower bounds, together with the treatment of discrete integer variables, have also been clarified. In addition, the mathematical direction of the stability constraints has been corrected, such that the calculated buckling factors are required to be no less than their allowable values.

Furthermore, the Pareto dominance relationship and the updating rule for the non-dominated solution set have been added to the algorithmic procedure. Therefore, the revised objective functions, design variables, constraint conditions, and Pareto-based screening process are now mathematically consistent throughout the optimization formulation.

(Pages 9,lines281-289; Pages 10-11,lines 330-333)

**Q3. The optimization algorithm lacks sufficient implementation details for reproducibility, including constraint handling, Pareto strategy, and algorithm parameters.**

**Author’s Response:**

Thank you for this valuable comment. Although the original manuscript described the processes of candidate-solution generation, finite element analysis, Pareto screening, and iterative updating through the optimization flowchart and detailed algorithmic steps, some parameter definitions, candidate-retention rules, and termination criteria were not sufficiently specified, which could indeed affect the reproducibility of the method. The authors’ previous work had already presented the basic procedures for candidate generation, removal of duplicate solutions, finite element constraint checking, calculation of morphactin concentration, and loop termination. However, the original manuscript did not fully specify the implementation parameters of these procedures in the present multi-objective optimization problem.

To address this issue, we supplemented and standardized the existing flowchart and algorithmic steps. The main revisions are as follows:

The maximum capacity of the candidate pool used for selecting the next growth point in each iteration was specified as k=50. When fewer than 50 valid candidates are available, all candidates are retained. When the number exceeds 50, the candidates are ranked according to their morphactin concentrations, and the top 95% are retained.

The MLP classification threshold was specified as 0.2. When the predicted feasibility probability is not lower than 0.2, the candidate solution proceeds to ANSYS finite element verification; otherwise, it is eliminated before finite element analysis. The final feasibility of all candidate solutions is still determined by the ANSYS results together with the structural constraints.

The Pareto-set updating rule was added. After each iteration, the newly generated feasible non-dominated solutions are merged with the existing Pareto solution set, followed by a new non-dominated sorting procedure. No fixed maximum capacity is imposed on the Pareto solution set in this study. The parameter k=50 is used only to limit the size of the candidate pool for selecting the next growth point and does not limit the number of Pareto solutions.

The termination criteria were clarified. The maximum number of iterations is 300 for the inner size and prestress optimization and 10 for the outer topology optimization. The corresponding loop is terminated when the prescribed maximum number of iterations is reached or when no valid candidate solution exists in the current cycle. Objective-function stability is not adopted as an additional termination criterion in this study.

The constraint-handling procedure was further clarified. The MLP is used only for preliminary screening. Candidate solutions that pass the MLP screening must still be analyzed using ANSYS and are allowed to enter the Pareto non-dominated analysis only when they simultaneously satisfy the displacement, strength, slenderness, local stability, overall stability, and cable-tension-state constraints.

Instead of adding a new flowchart or separate algorithm box, these parameters and rules were incorporated into the existing flowchart and algorithmic steps, and the descriptions in the figures, main text, and actual computational procedure were made consistent. Through these revisions, the procedures for candidate generation, screening, constraint handling, Pareto-set updating, and termination are now more clearly defined, thereby improving the clarity and reproducibility of the proposed method.

(Pages 18 , lines 552-567; )

**Q4. Validation of the improved PGSA is insufficient. Only limited benchmark tests are provided without statistical analysis.**

**Author’s Response:**

Thank you for this valuable comment. The two main improvement mechanisms of iPGSA and their benchmark validation have been described in detail in the authors’ previous study. In that study, unimodal and multimodal benchmark functions, together with a K6 reticulated-shell optimization example, were used to evaluate iPGSA, and the results demonstrated improvements in search efficiency and optimization performance compared with the conventional PGSA, GA, ANGA, and other methods.

To avoid substantial duplication with the previous study, the revised manuscript does not reproduce all of these benchmark examples. Instead, the relevant study has been cited and its main validation results have been briefly summarized. In addition, following the reviewer’s suggestion, multiple independent runs were conducted for the two benchmark functions considered in the present manuscript. Statistical indicators, including the mean best objective value, standard deviation, success rate, and average number of iterations, were added to evaluate the stability and repeatability of the algorithmic results. The corresponding statistical results have been added to Table 1.

(Pages 7 ,lines 237-240)

**Q5. No comparison is made with state-of-the-art topology optimization approaches such as SIMP, BESO, level-set, or gradient-based methods.**

**Author’s Response:**

Thank you for this valuable suggestion. In response to this comment to include recent advanced topology optimization methods, the Introduction has been expanded to include relevant studies on the enhanced proportional topology optimization method, the parameterized level-set method, and the asymptotic concentration–isogeometric analysis method, thereby providing a more comprehensive review of current topology optimization approaches.

It should be noted that the SIMP method, conventional BESO, level-set methods, and most gradient-based approaches mentioned by the reviewer are primarily developed for continuum topology optimization. Their basic design variables are generally material densities or boundary-description parameters defined within a fixed design domain. In contrast, the Suspen-Dome considered in this study is a discrete spatial member system with explicit connectivity relationships. Its design variables include the number of rings, number of radial divisions, arrangement intervals of the cable–strut system, discrete section indices, and cable prestress. In addition, the optimization must satisfy engineering constraints involving member strength, slenderness ratio, local stability, overall stability, and the tension-only behavior of cables.

Therefore, the two classes of methods differ fundamentally in terms of topology representation, design variables, and constraint formulation. Applying SIMP or level-set methods directly to the present problem would require the discrete member system to be reformulated as a continuum material design domain, or an additional mapping relationship to be established between member existence and material density. Such a reformulation would change the parameterized Suspen-Dome optimization problem considered in this study and would therefore make a fair quantitative comparison under identical design variables and constraints difficult.

For this reason, continuum topology optimization methods were not directly adopted for numerical comparison in the present study. Instead, GA, PSO, ANGA, and related heuristic optimization methods, which are more suitable for discrete structural variables and nonlinear engineering constraints, were selected as comparison algorithms. The revised manuscript further clarifies the distinction between continuum topology optimization and the discrete member-system topology optimization considered herein, together with the rationale for selecting the comparison methods.

In addition, the pointer-based method developed in this study based on the BESO concept does not directly employ the conventional BESO procedure of adding and removing finite elements. Rather, it draws on the bidirectional evolutionary concept of BESO to provide directional guidance for the discrete topological and stiffness-related parameters of the Suspen-Dome. This distinction has also been further clarified in the revised manuscript.

(Pages 2,lines 49-63; Pages 36, lines 957-965)

**Q6. The surrogate MLP model is insufficiently validated. Dataset generation, sample size, cross-validation, and robustness are not adequately discussed.**

**Author’s Response:**

Thank you for this valuable comment. We agree that, although the original manuscript presented the loss curve of the MLP model together with Accuracy, Precision, Recall, and F1-score, the description of the dataset generation procedure, sample size, and cross-validation was not sufficiently detailed.

In response, Section 3.2.5 has been expanded to provide additional information on the dataset generation procedure, the total number of samples, the numbers of feasible and infeasible samples, and the criteria used to assign the corresponding feasibility labels. The proportions used to divide the dataset into training, validation, and test sets, together with the main training parameters of the MLP model, have also been clarified.

In addition, five-fold stratified cross-validation was performed, and the mean and standard deviation of Accuracy, Precision, Recall, and F1-score were reported to evaluate the stability of the model under different data partitions. The cross-validation results are generally consistent with those obtained from the independent test set, indicating that the MLP screening model provides relatively stable classification performance.

It should be emphasized that the MLP model is used only as an auxiliary classifier for candidate pre-screening, with the purpose of eliminating clearly infeasible structural candidates before finite element analysis. All retained candidates and final optimization results are still verified using ANSYS. In addition, the statement in the original manuscript that “the number of ANSYS calls was reduced by 25.6%” has been corrected to “the average computational time was reduced by approximately 25.6%,” so that the description is consistent with the actual computational results.

(Pages 15, lines 448-460,465-469; Pages 16, lines 471-472)

**Q7. The proposed PFSD concept is heuristic and lacks theoretical justification.**

**Author’s Response:**

Thank you for this valuable comment. We agree that the original manuscript did not sufficiently explain the rationale behind the proposed PFSD concept. It should be clarified that PFSD is not intended as a new structural theory, but rather as a design strategy developed according to the different mechanical roles of the upper reticulated shell and the lower cable-strut system in a Suspen-Dome.

In the revised manuscript, additional explanations have been added in Section 3.2.4 to clarify its mechanical basis. Specifically, the upper reticulated shell is regarded as the primary load-carrying subsystem that provides global stiffness and stability reserve, and therefore should retain an appropriate stress margin rather than strictly satisfying the full-stress criterion. By contrast, the lower cable system mainly resists axial tension without buckling and is responsible for prestress regulation, making it more suitable for a near full-stress design. Therefore, PFSD represents a mechanics-based design strategy for balancing structural safety reserve and material utilization, instead of a newly established structural theory.

(Pages 14-15, lines 417-436)

**Q8. The optimization examples are limited, with no sensitivity or robustness studies.**

**Author’s Response:**

Thank you for this valuable suggestion. The original manuscript employed two optimization examples with different spans and topological configurations, namely a 100 m-span Kiewitt–Lamella Suspen-Dome and a 110 m-span rib-ring Suspen-Dome, to provide an initial assessment of the applicability of the proposed method to different Suspen-Dome configurations. However, we agree that the original manuscript did not specifically investigate the effects of load variation or the stochastic nature of the optimization algorithm.

In response, a new Section 6, entitled “Sensitivity and Robustness Study,” has been added to the revised manuscript, using the 100 m-span Kiewitt–Lamella Suspen-Dome presented in Section 4 as the representative example.

For the sensitivity analysis, complete optimization procedures were independently performed under load levels of 1.0, 1.2, and 1.4 times the baseline load, while the design-variable ranges, algorithmic parameters, constraint conditions, and termination criteria were kept unchanged. The optimized structural masses under the three load levels were 271.56 t, 299.77 t, and 301.10 t, respectively. Compared with the baseline case, the structural masses under the latter two load levels increased by 10.39% and 10.88%, respectively. The corresponding maximum vertical displacements were 20.90 mm, 45.56 mm, and 34.47 mm. These results indicate that the optimized structural mass is relatively less sensitive to the investigated load variation, whereas structural deformation and the resulting topology and member configuration are more sensitive to the load level. All optimized solutions obtained under the three load cases satisfied the relevant structural constraints, indicating that the proposed method can adapt the topology, member sections, and prestress parameters to different load levels and obtain feasible designs.

For the robustness analysis, the optimization was independently repeated using three different random seeds while maintaining identical initial conditions, design-variable ranges, and algorithmic parameters. All three runs resulted in the same overall topological parameters, namely ***Kn***=11 and ***Nx***=12, with cable–strut arrangement intervals of 1 and 3. The mean, standard deviation, and coefficient of variation of the structural mass were 277.27 t, 7.22 t, and 2.60%, respectively. For the maximum vertical displacement, the corresponding values were 25.32 mm, 3.98 mm, and 15.72%, respectively. These results show good consistency in structural mass and overall topology among different stochastic search paths, although some variations remain in the local section configurations and deformation responses. Such variations reflect the trade-off between structural weight and deformation performance inherent in the multi-objective optimization problem.

Through these revisions, the manuscript now includes both a sensitivity analysis of the optimization results under different load levels and a robustness analysis based on independent runs with different random seeds.

(Pages 32-33 ,lines 812-866)

**Q9. Engineering conclusions are largely qualitative and not fully supported by quantitative evidence.**

**Author’s Response:**

Thank you for this valuable comment. We agree that some statements in the conclusions of the original manuscript relied mainly on qualitative descriptions such as “significantly reduced,” “small displacement,” and “good stability,” without sufficiently using the quantitative results of structural mass, displacement, and buckling analyses already presented in the main text.

In response, quantitative results for the two optimization examples have been added after Conclusion (5) in the revised manuscript. For the 100 m-span Kiewitt–Lamella Suspen-Dome, the structural mass was reduced from 1380.00 t to 271.56 t, corresponding to a reduction of 80.32%. The maximum vertical displacement was 20.90 mm, while the nonlinear buckling factor and eigenvalue buckling factor were 3.32 and 8.15, respectively. For the 110 m-span rib-ring Suspen-Dome, the structural mass was reduced from 336.00 t to 176.20 t, corresponding to a reduction of 47.56%. The maximum vertical displacement was 13.24 mm, and the nonlinear buckling factor and eigenvalue buckling factor were 3.14 and 6.68, respectively.

Accordingly, the original qualitative conclusion has been revised to state that the proposed method can substantially reduce structural mass while maintaining relatively small vertical deformation and an appropriate overall stability reserve. The corresponding conclusions are now supported by specific quantitative indicators, including the structural mass reduction ratio, maximum vertical displacement, and buckling factors.

In addition, stronger statements such as “can be directly applied to engineering practice” have been moderated and replaced by “can provide quantitative references for the preliminary design and parameter selection of large-span Suspen-Dome structures.” This revision ensures that the engineering implications of the conclusions remain consistent with the scope of evidence supported by the numerical analyses conducted in this study.

(Pages 35, lines 921-930; )

**Q10. The manuscript requires substantial improvement in language, equation presentation, and organization.**

**Author’s Response:**

Thank you for this valuable suggestion. In response, we conducted a further systematic review and language revision of the entire manuscript, with particular attention to sentences that could be ambiguous, unclear, or difficult to understand. Relevant expressions were reorganized and revised where necessary. Several overly long sentences or sentences with unclear logical relationships were also divided or rewritten, and the terminology, abbreviations, symbols, and related expressions were further standardized throughout the manuscript. In addition, grammatical, spelling, and formatting issues were carefully rechecked and corrected.

These revisions have further improved the clarity, logical coherence, and readability of the manuscript, and the corresponding changes have been incorporated into the revised version.

**Reviewer #3: This manuscript presents a multiobjective optimization algorithm and a shift from the common separated design approach for Suspen-Dome structures toward a holistic, integrated optimization that simultaneously addresses size, geometry, and topology of the upper shell along with the topology and prestress of the lower cable-strut system. Although the manuscript is well written, there are some ambiguities in it, some of which are mentioned:**

**•Q1: There are some computational ambiguities in this manuscript. For example, on page 4, the creation of the connectivity matrix requires an example.**

**Author’s Response:**

Thank you for this careful comment. We agree that the original manuscript provided the definition and construction procedure of the connectivity matrix without a specific example, which could make the formulation difficult to follow. In response, a simple illustrative example has been added to the corresponding part of the revised manuscript. Using a local structural system with clearly defined node numbers and member connectivity, the example demonstrates how the connectivity matrix is constructed from the end-node information of each member. This addition clarifies the correspondence among node numbering, member connectivity, and the entries of the connectivity matrix, making the construction procedure more intuitive and easier to understand.

( Pages 5, lines 167-174)

**• Q2:On page 5, can “mmm” be represented mathematically in R3 space, or can the authors come up with a better mathematical expression for the representation?**

**Author’s Response:**

Thank you for this careful comment. We agree that the mathematical representation in this part of the original manuscript was not sufficiently precise. In response, we re-examined the mathematical formulation and revised the relevant expressions . The corresponding symbols and their meanings have also been further clarified to improve the rigor and readability of the formulation. The relevant revisions have been incorporated into the revised manuscript.

(Pages 6 ,lines205)
**• Q3:The author should define all parameters used, for example the parameter β in equation 7.**

**Author’s Response:**

Thank you for this careful comment. In response, the definition and meaning of the parameter β in Eq. (8)( after correct ) have been added at the corresponding location in the revised manuscript. In addition, all parameters and symbols used in the equations throughout the manuscript were further checked. Definitions have been added or clarified for parameters whose meanings were not sufficiently explained at their first occurrence in the original manuscript, thereby improving the completeness and consistency of the mathematical notation throughout the paper.

(Pages 10, lines 319, Table 3)

**• Q4:The domain space for design or population variables, such as Kn, should be defined.**

**Author’s Response:**

Thank you for this careful comment. We agree that, although design variables such as ***Kn*** and ***Nx*** were introduced in the original manuscript, their admissible ranges and the basis for determining these ranges were not sufficiently specified. In response, the revised manuscript now explicitly defines the domains of the main design variables, including the upper and lower bounds and integer requirements for the topological variables *Kn* and ***Nx*** , the arrangement intervals of the cable–strut system, and the other discrete design variables. This revision provides a clearer definition of the optimization search space.

It should be noted that the ranges of these topological variables are not selected arbitrarily. Taking the circumferential division number ***Nx*** as an example, its value is determined by considering the structural span, shell radius, and target grid size. In this study, the outer-ring member length and the radial grid dimension are controlled within comparable ranges, with the principal grid dimensions maintained at approximately 6–10 m. This criterion provides relatively uniform grid geometry while also considering overall structural stiffness, stability of compression members, and practical constructability. Based on this criterion, the initial circumferential division numbers were set to 26 and 40 for the 100 m-span Kiewitt–Lamella Suspen-Dome and the 110 m-span rib-ring Suspen-Dome, respectively. The corresponding variable ranges and their discrete characteristics have now been explicitly specified in the revised manuscript.

(Pages 19, lines 579-586)

**• Q5:In Table 1. The population used or produced during the pressing process should be written for each algorithm.**

**Author’s Response:**

Thank you for this careful comment. In response, Table 2(after correct) has been revised to include the population size adopted or generated by each comparison algorithm during the optimization process. This additional information further clarifies the parameter settings and computational scale of the different algorithms. The corresponding population-size information is now consistently reported in Table 2 of the revised manuscript, thereby improving the transparency and reproducibility of the algorithmic comparison.

(Pages 8, lines 254-255)
