# Supplementary material for: Surrogate-assisted multi-objective topology optimization design method for prefabricated large-span Suspen-Dome based on structural-mapping-method and improved-PGSA
Source: PLoS One. 2026 Nov 2;21(11):e0361862. doi: 10.1371/journal.pone.0361862 (PMC13726994; doi:10.1371/journal.pone.0361862)
Supplement: Supplementary file 2 [file pone.0361862.s004.docx]

Thank you for submitting your manuscript to PLOS One. After careful consideration, we feel that it has merit but does not fully meet PLOS One’s publication criteria as it currently stands. Therefore, we invite you to submit a revised version of the manuscript that addresses the points raised during the review process.

Please, address the concerns related to originality, methodological significance, and validation as stated by one of the reviewers.

**Author’s Response:**

Thank you for again raising the important concerns regarding the **originality, methodological significance, and adequacy of validation** of the manuscript. We understand that the central concern in this round is no longer simply whether the methodological procedure is sufficiently described, but whether the integrated optimization treatment adopted in this study produces a substantive effect beyond a straightforward combination or problem-specific adaptation of existing techniques, and whether the available numerical evidence adequately supports this methodological significance. In response, we have further revised both the positioning of the contribution and the validation presented in the manuscript.

First, regarding **originality**, we have further clarified that the present study does not re-propose the Structural Mapping Method (SMM) or iPGSA themselves as new methods, nor are the MLP screening model and PFSD strategy claimed as independent general-purpose optimization theories. The principal contribution of this study is the extension of the existing modeling and search procedures to the coupled discrete topology–size–prestress optimization problem of Suspen-Dome structures, in which the topology, geometry, and member sections of the upper reticulated shell are optimized together with the layout, member sizing, and cable prestress of the lower cable–strut system within the same design space and under the same structural constraints. The Introduction and Discussion have therefore been revised to more clearly define this problem-oriented methodological contribution and to avoid overstating the independent novelty of the auxiliary components.

Second, regarding **methodological significance**, we agreed that the original manuscript mainly explained the need for integrated optimization conceptually, without providing sufficiently direct comparative evidence. Therefore, a new Section 6.3, entitled “Comparison between Sequential and Integrated Optimization Strategies,” has been added using the 100 m-span Kiewitt–Lamella Suspen-Dome as a controlled example. In the sequential strategy, the upper reticulated shell is optimized first. During this stage, the lower cable–strut system is not treated as a set of optimization variables; however, its nodal connectivity, geometric parameters, and prestress equilibrium state are updated according to the current upper-shell configuration to maintain geometric compatibility and structural equilibrium. After the representative upper-shell solution is obtained, its topology, geometry, and member sections are fixed, and the lower cable–strut system is subsequently optimized. In contrast, the integrated strategy allows the design variables associated with both subsystems to be adjusted within the same optimization process. The two strategies use identical initial structural conditions, material properties, loading conditions, objective functions, structural constraints, design-variable bounds, finite-element models, and algorithmic parameters. Thus, the comparison primarily reflects the effect of sequential versus integrated treatment of the two mechanically coupled subsystems rather than differences in the underlying optimization algorithm or structural analysis model.

The newly added Table 12 shows that the two strategies lead to different structural configurations and performance characteristics. The structural mass obtained by the integrated strategy is 271.56 t, compared with 303.50 t for the sequential strategy, corresponding to a reduction of 10.52%. The maximum vertical displacement is reduced from 30.06 mm to 20.90 mm. Differences are also observed in the resulting topological parameters: Kn changes from 13 in the sequential strategy to 11 in the integrated strategy, while the cable–strut arrangement interval changes from 1,1 to 1,3. The nonlinear buckling factor of the integrated solution is slightly lower than that of the sequential solution (3.32 versus 3.79), but both solutions satisfy the prescribed stability requirements. These results demonstrate that sequential and integrated treatment of the upper and lower structural systems leads to different attainable design solutions under otherwise identical structural constraints. In particular, allowing the topology, size, and prestress-related variables of the two mechanically coupled subsystems to interact within the same search process results in a lighter structural solution with smaller vertical deformation. We believe that this controlled comparison provides more direct numerical evidence of the methodological effect of the integrated treatment than was available in the previous version of the manuscript.

Third, regarding **validation**, the validation strategy has been reorganized and strengthened. The revised manuscript now includes: (1) algorithm-level validation of iPGSA using benchmark functions and a reticulated-shell optimization example with comparison algorithms; (2) complete optimization of two Suspen-Dome configurations, namely the 100 m-span Kiewitt–Lamella Suspen-Dome and the 110 m-span rib-ring Suspen-Dome; (3) independent testing and five-fold cross-validation of the MLP screening model; (4) sensitivity analysis under different load levels; (5) robustness analysis using different random seeds; and (6) the newly added controlled comparison between sequential and integrated optimization strategies. At the same time, statements regarding general applicability and superiority have been further moderated. The present study does not claim universal superiority over all topology optimization methods or unrestricted applicability to arbitrary spatial structural systems; the conclusions are limited to the investigated Suspen-Dome configurations and parameter ranges.

Through these revisions, we have sought to more clearly distinguish the problem-oriented methodological extension developed in the present study from the existing underlying methods and to provide direct comparative evidence for the practical significance of the integrated optimization treatment. We sincerely appreciate the reviewer’s comment, which helped us further clarify the scope of the claimed originality and strengthen the corresponding comparative validation and scientific evidence.

(Pages 3-4, lines 126-155; Pages 33, lines 823; Pages34-35, lines 878-911; Pages 35-36, lines 915-923; Pages 36, lines944-950; Pages 37, lines971-976 )

**Reviewer #2:** Thank you for the authors' detailed responses and revisions. However, after carefully considering the revised manuscript, I am still not convinced that the major concerns raised in the previous review have been adequately resolved.

The central issue remains the limited methodological novelty and insufficient validation of the proposed framework. The authors now clarify that SMM and iPGSA are existing methods and that the main contribution is an extension based on a BESO-inspired pointer adjustment mechanism for the coupled discrete optimization of Suspen-Dome structures. However, the revised manuscript still does not provide sufficiently convincing evidence that this mechanism constitutes a substantial methodological advance beyond the integration and problem-specific adaptation of existing optimization techniques. The additional MLP screening and PFSD strategy also appear largely auxiliary or heuristic, and their generality and effectiveness are not sufficiently demonstrated.

The authors have added more implementation details, cross-validation, sensitivity analysis, and robustness tests. Nevertheless, these additions mainly improve the completeness of the presentation rather than fundamentally addressing the above scientific concern. In particular, the validation remains limited to a small number of structural examples, and there is still insufficient comparative evidence to demonstrate the superiority, general applicability, and robustness of the proposed methodology over appropriate alternative approaches.

Therefore, although the revised manuscript is clearer than the previous version, I consider that the fundamental concerns regarding originality, methodological significance, and rigorous validation remain unresolved. In my opinion, addressing these issues would require substantial further methodological development and validation rather than another round of editorial revision.

I therefore cannot recommend publication of the manuscript.

**Author’s Response:**

Thank you for again raising the important concerns regarding the **originality, methodological significance, and adequacy of validation** of the manuscript. We understand that the central concern in this round is no longer simply whether the methodological procedure is sufficiently described, but whether the integrated optimization treatment adopted in this study produces a substantive effect beyond a straightforward combination or problem-specific adaptation of existing techniques, and whether the available numerical evidence adequately supports this methodological significance. In response, we have further revised both the positioning of the contribution and the validation presented in the manuscript.

First, regarding **originality**, we have further clarified that the present study does not re-propose the Structural Mapping Method (SMM) or iPGSA themselves as new methods, nor are the MLP screening model and PFSD strategy claimed as independent general-purpose optimization theories. The principal contribution of this study is the extension of the existing modeling and search procedures to the coupled discrete topology–size–prestress optimization problem of Suspen-Dome structures, in which the topology, geometry, and member sections of the upper reticulated shell are optimized together with the layout, member sizing, and cable prestress of the lower cable–strut system within the same design space and under the same structural constraints. The Introduction and Discussion have therefore been revised to more clearly define this problem-oriented methodological contribution and to avoid overstating the independent novelty of the auxiliary components.

Second, regarding **methodological significance**, we agreed that the original manuscript mainly explained the need for integrated optimization conceptually, without providing sufficiently direct comparative evidence. Therefore, a new Section 6.3, entitled “Comparison between Sequential and Integrated Optimization Strategies,” has been added using the 100 m-span Kiewitt–Lamella Suspen-Dome as a controlled example. In the sequential strategy, the upper reticulated shell is optimized first. During this stage, the lower cable–strut system is not treated as a set of optimization variables; however, its nodal connectivity, geometric parameters, and prestress equilibrium state are updated according to the current upper-shell configuration to maintain geometric compatibility and structural equilibrium. After the representative upper-shell solution is obtained, its topology, geometry, and member sections are fixed, and the lower cable–strut system is subsequently optimized. In contrast, the integrated strategy allows the design variables associated with both subsystems to be adjusted within the same optimization process. The two strategies use identical initial structural conditions, material properties, loading conditions, objective functions, structural constraints, design-variable bounds, finite-element models, and algorithmic parameters. Thus, the comparison primarily reflects the effect of sequential versus integrated treatment of the two mechanically coupled subsystems rather than differences in the underlying optimization algorithm or structural analysis model.

The newly added Table 12 shows that the two strategies lead to different structural configurations and performance characteristics. The structural mass obtained by the integrated strategy is 271.56 t, compared with 303.50 t for the sequential strategy, corresponding to a reduction of 10.52%. The maximum vertical displacement is reduced from 30.06 mm to 20.90 mm. Differences are also observed in the resulting topological parameters: Kn changes from 13 in the sequential strategy to 11 in the integrated strategy, while the cable–strut arrangement interval changes from 1,1 to 1,3. The nonlinear buckling factor of the integrated solution is slightly lower than that of the sequential solution (3.32 versus 3.79), but both solutions satisfy the prescribed stability requirements. These results demonstrate that sequential and integrated treatment of the upper and lower structural systems leads to different attainable design solutions under otherwise identical structural constraints. In particular, allowing the topology, size, and prestress-related variables of the two mechanically coupled subsystems to interact within the same search process results in a lighter structural solution with smaller vertical deformation. We believe that this controlled comparison provides more direct numerical evidence of the methodological effect of the integrated treatment than was available in the previous version of the manuscript.

Third, regarding **validation**, the validation strategy has been reorganized and strengthened. The revised manuscript now includes: (1) algorithm-level validation of iPGSA using benchmark functions and a reticulated-shell optimization example with comparison algorithms; (2) complete optimization of two Suspen-Dome configurations, namely the 100 m-span Kiewitt–Lamella Suspen-Dome and the 110 m-span rib-ring Suspen-Dome; (3) independent testing and five-fold cross-validation of the MLP screening model; (4) sensitivity analysis under different load levels; (5) robustness analysis using different random seeds; and (6) the newly added controlled comparison between sequential and integrated optimization strategies. At the same time, statements regarding general applicability and superiority have been further moderated. The present study does not claim universal superiority over all topology optimization methods or unrestricted applicability to arbitrary spatial structural systems; the conclusions are limited to the investigated Suspen-Dome configurations and parameter ranges.

Through these revisions, we have sought to more clearly distinguish the problem-oriented methodological extension developed in the present study from the existing underlying methods and to provide direct comparative evidence for the practical significance of the integrated optimization treatment. We sincerely appreciate the reviewer’s comment, which helped us further clarify the scope of the claimed originality and strengthen the corresponding comparative validation and scientific evidence.

(Pages 3-4, lines 126-155; Pages 33, lines 823; Pages34-35, lines 878-911; Pages 35-36, lines 915-923; Pages 36, lines944-950; Pages 37, lines971-976 )
