# Supplementary material for: Surrogate-assisted multi-objective topology optimization design method for prefabricated large-span Suspen-Dome based on structural-mapping-method and improved-PGSA
Source: PLoS One. 2026 Nov 2;21(11):e0361862. doi: 10.1371/journal.pone.0361862 (PMC13726994; doi:10.1371/journal.pone.0361862)
Supplement: Peer review history

# Peer review: Decision Letter 0

**DOI:** 10.1371/journal.pone.0361862.r001

**Authors:** Antonio Riveiro Rodríguez

19 Jul 2026

PLOS One

Dear Dr. Dai,

Thank you for submitting your manuscript to PLOS ONE. After careful consideration, we feel that it has merit but does not fully meet PLOS ONE’s publication criteria as it currently stands. Therefore, we invite you to submit a revised version of the manuscript that addresses the points raised during the review process.

Please submit your revised manuscript by Sep 17 2026 11:59PM. If you will need more time than this to complete your revisions, please reply to this message or contact the journal office at plosone@plos.org. When you're ready to submit your revision, log on to https://www.editorialmanager.com/pone/ and select the 'Submissions Needing Revision' folder to locate your manuscript file.

- A letter that responds to each point raised by the academic editor and reviewer(s). You should upload this letter as a separate file labeled 'Response to Reviewers'.
- A marked-up copy of your manuscript that highlights changes made to the original version. You should upload this as a separate file labeled 'Revised Manuscript with Track Changes'.
- An unmarked version of your revised paper without tracked changes. You should upload this as a separate file labeled 'Manuscript'.

If you would like to make changes to your financial disclosure, please include your updated statement in your cover letter. Guidelines for resubmitting your figure files are available below the reviewer comments at the end of this letter.

If applicable, we recommend that you deposit your laboratory protocols in protocols.io to enhance the reproducibility of your results. Protocols.io assigns your protocol its own identifier (DOI) so that it can be cited independently in the future. For instructions see: https://journals.plos.org/plosone/s/submission-guidelines#loc-laboratory-protocols. Additionally, PLOS ONE offers an option for publishing peer-reviewed Lab Protocol articles, which describe protocols hosted on protocols.io. Read more information on sharing protocols at https://plos.org/protocols?utm_medium=editorial-email&utm_source=authorletters&utm_campaign=protocols.

As the corresponding author, your ORCID iD is verified in the submission system and will appear in the published article. PLOS supports the use of ORCID, and we encourage all coauthors to register for an ORCID iD and use it as well. Please encourage your coauthors to verify their ORCID iD within the submission system before final acceptance, as unverified ORCID iDs will not appear in the published article. *Only*  the individual author can complete the verification step; PLOS staff *cannot*  verify ORCID iDs on behalf of authors.

We look forward to receiving your revised manuscript.

Kind regards,

Antonio Riveiro Rodríguez, PhD

Academic Editor

PLOS One

Journal Requirements:

When submitting your revision, we need you to address these additional requirements.

1.Please ensure that your manuscript meets PLOS ONE's style requirements, including those for file naming. The PLOS ONE style templates can be found at

https://journals.plos.org/plosone/s/file?id=wjVg/PLOSOne_formatting_sample_main_body.pdf and

https://journals.plos.org/plosone/s/file?id=ba62/PLOSOne_formatting_sample_title_authors_affiliations.pdf

2. PLOS requires an ORCID iD for the corresponding author in Editorial Manager on papers submitted after December 6th, 2016. Please ensure that you have an ORCID iD and that it is validated in Editorial Manager. To do this, go to ‘Update my Information’ (in the upper left-hand corner of the main menu), and click on the Fetch/Validate link next to the ORCID field. This will take you to the ORCID site and allow you to create a new iD or authenticate a pre-existing iD in Editorial Manager.

3. Please note that PLOS One has specific guidelines on code sharing for submissions in which author-generated code underpins the findings in the manuscript. In these cases, we expect all author-generated code to be made available without restrictions upon publication of the work. Please review our guidelines at https://journals.plos.org/plosone/s/materials-and-software-sharing#loc-sharing-code and ensure that your code is shared in a way that follows best practice and facilitates reproducibility and reuse.

4. Thank you for stating the following financial disclosure:

“The work is mainly supported by the Key Program of the National Natural Science Foundation of China under Grant No. 52130809.”

Please state what role the funders took in the study.  If the funders had no role, please state: "The funders had no role in study design, data collection and analysis, decision to publish, or preparation of the manuscript."

If this statement is not correct you must amend it as needed.

Please include this amended Role of Funder statement in your cover letter; we will change the online submission form on your behalf.

5. Thank you for stating the following in the Acknowledgments Section of your manuscript:

“The work is mainly supported by the Key Program of the National Natural Science Foundation of China under Grant No. 52130809.”

We note that you have provided funding information that is not currently declared in your Funding Statement. However, funding information should not appear in the Acknowledgments section or other areas of your manuscript. We will only publish funding information present in the Funding Statement section of the online submission form.

Please remove any funding-related text from the manuscript and let us know how you would like to update your Funding Statement. Currently, your Funding Statement reads as follows:

“The work is mainly supported by the Key Program of the National Natural Science Foundation of China under Grant No. 52130809.”

Please include your amended statements within your cover letter; we will change the online submission form on your behalf.

6. In the online submission form, you indicated that “Data will be available if request.”

All PLOS journals now require all data underlying the findings described in their manuscript to be freely available to other researchers, either 1. In a public repository, 2. Within the manuscript itself, or 3. Uploaded as supplementary information.

This policy applies to all data except where public deposition would breach compliance with the protocol approved by your research ethics board. If your data cannot be made publicly available for ethical or legal reasons (e.g., public availability would compromise patient privacy), please explain your reasons on resubmission and your exemption request will be escalated for approval.

7. Please ensure that you refer to Figure 1, 5, 6 and 21 in your text as, if accepted, production will need this reference to link the reader to the figure.

8. We note you have included a table to which you do not refer in the text of your manuscript. Please ensure that you refer to Table 1 in your text; if accepted, production will need this reference to link the reader to the Table.

9. If the reviewer comments include a recommendation to cite specific previously published works, please review and evaluate these publications to determine whether they are relevant and should be cited. There is no requirement to cite these works unless the editor has indicated otherwise.

Additional Editor Comments:

The manuscript's principal weakness is that it relies on the integration of established methods without demonstrating a clear methodological advance over existing approaches. Critical mathematical details are missing, key algorithmic components are insufficiently described, validation of both the optimization and surrogate models is inadequate, comparisons with state-of-the-art methods are lacking, and the claimed innovations are not sufficiently substantiated.

While the work may eventually be suitable after substantial redevelopment and more rigorous validation, the current version does not provide the level of novelty, methodological rigor, reproducibility, and scientific evidence required for publication.

The manuscript requires major conceptual, methodological, and validation-related improvements before it can be considered for publication in this journal.

[Note: HTML markup is below. Please do not edit.]

Reviewer's Responses to Questions

**Comments to the Author**

1. Is the manuscript technically sound, and do the data support the conclusions?

Reviewer #1: Yes

Reviewer #2: No

Reviewer #3: Yes

**********

2. Has the statistical analysis been performed appropriately and rigorously?

Reviewer #1: Yes

Reviewer #2: N/A

Reviewer #3: No

**********

3. Have the authors made all data underlying the findings in their manuscript fully available?

The PLOS Data policy

Reviewer #1: No

Reviewer #2: No

Reviewer #3: No

**********

4. Is the manuscript presented in an intelligible fashion and written in standard English?

Reviewer #1: Yes

Reviewer #2: No

Reviewer #3: Yes

**********

Reviewer #1: This manuscript focuses on investigating the integrated optimization of prefabricated large-span Suspen-Dome structures by simultaneously considering the size, geometric configuration, and topology of the upper reticulated shell, together with the topology and cable forces of the lower cable-strut system. To ensure practical applicability, the design variables are classified into topological and size variables. The former define the structural configuration and connectivity, while the latter determine material-related properties. The optimization aims to maximize stiffness and minimize weight, subject to member slenderness, material strength, local stability, and overall structural stability constraints. An multilayer perceptron (MLP)-based candidate screening model is introduced to accelerate the optimization algorithm and reduce unnecessary ANSYS analyses. A partial fully stressed design (PFSD) concept is proposed, in which the reticulated shell serves as a structural safety reserve. The proposed structural mapping method-improved plant growth algorithm (SMM-iPGSA) is validated through two examples. Results show that the optimized structures exhibit improved stability, more uniform stress distribution, and displacements well below the allowable limits.

The manuscript describes a technically sound piece of scientific research with data that supports the conclusions. Experiments have been conducted rigorously, with appropriate controls, replication, and sample sizes. The conclusions are drawn appropriately based on the data presented. The statistical analysis has been performed appropriately and rigorously. The manuscript is presented in an intelligible fashion and written in Standard English. Therefore, the evaluation of this manuscript is generally favorable and positive. However, there are still some issues that need to be carefully addressed, as listed below:

1. For the problem addressed in this manuscript, what is the advantage of the proposed method compared with other existing similar approaches? A balanced discussion of both advantages and limitations will help clarify the contribution and novelty of the proposed method.

2. The introduction section needs to be further strengthened. Specifically, topology optimization, as the most comprehensive category of structural optimization and a powerful design tool, covers a wide range of application fields. There are many commonly-used methods for structural topology optimization in recent times, such as the enhanced proportional topology optimization method (https://doi.org/10.1080/15397734.2026.2661100), the parameterized level set method (https://doi.org/10.1080/15397734.2025.2571736), and the asymptotic concentration method (https://doi.org/10.3934/era.2023196). If possible, it is recommended to briefly comment on them to enrich the scalability of this manuscript.

3. Most of the images in the manuscript have low resolution and remain blurry even when enlarged, which makes it difficult to see their content clearly. Especially the images in Figures 3, 6, 8, 10, 11, 12, 14, 15, 16, 17, 19, 20, 21, 23, 24, 25, 26, 27. If applicable, it is recommended to carefully make appropriate modifications to the similar issues together.

4. If possible, it is recommended to change the color of the images in Tables 4 and 6, so as to enhance content recognition and readability.

5. The PLOS Data policy requires authors to make all data underlying the findings described in their manuscript fully available without restriction, with rare exception (please refer to the Data Availability Statement in the manuscript PDF file). The data should be provided as part of the manuscript or its supporting information, or deposited to a public repository. Although some data are provided in the manuscript, it does not seem to meet the requirements of the PLOS Data policy. Therefore, it is suggested that the authors make appropriate modifications based on the specific circumstances.

6. PLOS ONE does not copyedit accepted manuscripts, so the language in submitted articles must be clear, correct, and unambiguous. Any typographical or grammatical errors should be corrected at revision, so the specific errors are noted as follows.

(1) When abbreviations first appear, they should be accompanied by their corresponding full names. This is a writing principle. However, in the abstract (line 18) and main text (line 120), the abbreviation “MLP” violates this principle. Moreover, the abbreviation “GSI&PS-PGSA” (line 203) violates this principle. If applicable, it is recommended to carefully make appropriate modifications to the similar issues together.

(2) The citations in the text should be accurate. This is also a writing principle. However, both "Kaveh [8]" (line 42) and "Adam, by contrast, considered minimizing member stresses and maximizing overall structural stiffness [31]" (lines 95-96) violate this principle, as both References [8] and [31] have more than one contributor. If applicable, it is recommended to carefully make appropriate modifications to the similar issues together.

(3) The typographical errors in the text should be eliminated. This is also a writing principle. However, "Examples 1" (line 199) violates this principle, and its correct form should be 'Example 1'. If applicable, it is recommended to carefully make appropriate modifications to the similar issues together.

(4) All Figures should be mentioned in the main text. This is also a writing principle. However, Figure 1 was not mentioned in the main text, which violates this principle. If applicable, it is recommended to carefully make appropriate modifications to the similar issues together.

7. If possible, it is recommended to carefully check and revise the entire manuscript, strive to eliminate any ambiguities and unreadable points, and thus improve the presentation of the manuscript.

Reviewer #2: The manuscript presents a surrogate-assisted multi-objective topology optimization framework for prefabricated Suspen-Dome structures by integrating a structural mapping method (SMM), an improved plant growth simulation algorithm (iPGSA), a neural-network-based screening strategy, and a partial fully stressed design concept. Although the topic is relevant to structural optimization, the manuscript suffers from substantial methodological and scientific deficiencies. The claimed novelty is not sufficiently demonstrated, and many important methodological details are missing or lack rigorous validation. Therefore, I do not recommend publication in its current form.

Major comments:

1. The claimed methodological novelty is weak. The work mainly integrates existing methods rather than proposing a fundamentally new optimization methodology.

2. The mathematical formulation is incomplete and inconsistent. The multi-objective formulation is mathematically unclear and the definition of structural stiffness is questionable.

3. The optimization algorithm lacks sufficient implementation details for reproducibility, including constraint handling, Pareto strategy, and algorithm parameters.

4. Validation of the improved PGSA is insufficient. Only limited benchmark tests are provided without statistical analysis.

5. No comparison is made with state-of-the-art topology optimization approaches such as SIMP, BESO, level-set, or gradient-based methods.

6. The surrogate MLP model is insufficiently validated. Dataset generation, sample size, cross-validation, and robustness are not adequately discussed.

7. The proposed PFSD concept is heuristic and lacks theoretical justification.

8. The optimization examples are limited, with no sensitivity or robustness studies.

9. Engineering conclusions are largely qualitative and not fully supported by quantitative evidence.

10. The manuscript requires substantial improvement in language, equation presentation, and organization.

Reviewer #3: This manuscript presents a multiobjective optimization algorithm and a shift from the common separated design approach for Suspen-Dome structures toward a holistic, integrated optimization that simultaneously addresses size, geometry, and topology of the upper shell along with the topology and prestress of the lower cable-strut system. Although the manuscript is well written, there are some ambiguities in it, some of which are mentioned:

• There are some computational ambiguities in this manuscript. For example, on page 4, the creation of the connectivity matrix requires an example.

• On page 5, can “mmm” be represented mathematically in R3 space, or can the authors come up with a better mathematical expression for the representation?

• The author should define all parameters used, for example the parameter β in equation 7.

• The domain space for design or population variables, such as Kn, should be defined.

• In Table 1. The population used or produced during the pressing process should be written for each algorithm.

**********

what does this mean?). If published, this will include your full peer review and any attached files.

If you choose “no”, your identity will remain anonymous but your review may still be made public.

**Do you want your identity to be public for this peer review?**  For information about this choice, including consent withdrawal, please see our Privacy Policy

Reviewer #1: No

Reviewer #2: No

Reviewer #3: No

**********

[NOTE: If reviewer comments were submitted as an attachment file, they will be attached to this email and accessible via the submission site. Please log into your account, locate the manuscript record, and check for the action link "View Attachments". If this link does not appear, there are no attachment files.]

To ensure your figures meet our technical requirements, please review our figure guidelines: https://journals.plos.org/plosone/s/figures

You may also use PLOS’s free figure tool, NAAS, to help you prepare publication quality figures: https://journals.plos.org/plosone/s/figures#loc-tools-for-figure-preparation.

NAAS will assess whether your figures meet our technical requirements by comparing each figure against our figure specifications.

# Author comment: Author response to Decision Letter 1

**DOI:** 10.1371/journal.pone.0361862.r002

10 Aug 2026

Reviewer #1: This manuscript focuses on investigating the integrated optimization of prefabricated large-span Suspen-Dome structures by simultaneously considering the size, geometric configuration, and topology of the upper reticulated shell, together with the topology and cable forces of the lower cable-strut system. To ensure practical applicability, the design variables are classified into topological and size variables. The former define the structural configuration and connectivity, while the latter determine material-related properties. The optimization aims to maximize stiffness and minimize weight, subject to member slenderness, material strength, local stability, and overall structural stability constraints. An multilayer perceptron (MLP)-based candidate screening model is introduced to accelerate the optimization algorithm and reduce unnecessary ANSYS analyses. A partial fully stressed design (PFSD) concept is proposed, in which the reticulated shell serves as a structural safety reserve. The proposed structural mapping method-improved plant growth algorithm (SMM-iPGSA) is validated through two examples. Results show that the optimized structures exhibit improved stability, more uniform stress distribution, and displacements well below the allowable limits.

The manuscript describes a technically sound piece of scientific research with data that supports the conclusions. Experiments have been conducted rigorously, with appropriate controls, replication, and sample sizes. The conclusions are drawn appropriately based on the data presented. The statistical analysis has been performed appropriately and rigorously. The manuscript is presented in an intelligible fashion and written in Standard English. Therefore, the evaluation of this manuscript is generally favorable and positive. However, there are still some issues that need to be carefully addressed, as listed below:

Q1. For the problem addressed in this manuscript, what is the advantage of the proposed method compared with other existing similar approaches? A balanced discussion of both advantages and limitations will help clarify the contribution and novelty of the proposed method.

Author’s Response:

Thank you for this valuable comment. We agree that the original manuscript did not sufficiently discuss the advantages of the proposed method over existing approaches or clearly define its range of applicability. In response, a more balanced discussion of the advantages and limitations of the proposed method has been added to the revised manuscript.

Specifically, the main advantages of the proposed method are as follows: the topology, size, and prestress variables of both the upper reticulated shell and the lower cable–strut system are simultaneously considered within a unified optimization framework; the structural mapping method enables coordinated representation of different types of design variables; the MLP-based candidate screening model reduces unnecessary finite element analyses; and the PFSD strategy coordinates structural safety reserve with material utilization efficiency. Compared with existing methods that optimize only an individual subsystem or adopt a fixed topology, the proposed method provides a broader design space and improved applicability to engineering design.

At the same time, the revised manuscript explicitly clarifies the limitations of the proposed method. Its performance depends on the coverage of the training samples, while dynamic response, construction stages, and long-term service performance have not yet been considered. In addition, the applicability of the PFSD strategy to other types of spatial structures requires further validation. These additions provide a more objective definition of the contributions, applicability, and limitations of the proposed method, as well as directions for future research.

Q2. The introduction section needs to be further strengthened. Specifically, topology optimization, as the most comprehensive category of structural optimization and a powerful design tool, covers a wide range of application fields. There are many commonly-used methods for structural topology optimization in recent times, such as the enhanced proportional topology optimization method (https://doi.org/10.1080/15397734.2026.2661100), the parameterized level set method (https://doi.org/10.1080/15397734.2025.2571736), and the asymptotic concentration method (https://doi.org/10.3934/era.2023196). If possible, it is recommended to briefly comment on them to enrich the scalability of this manuscript.

Author’s Response:

Thank you for this valuable suggestion. We agree that topology optimization is an important and widely applicable design approach in the field of structural optimization, and that an appropriate discussion of representative recent developments can provide a more comprehensive research background and better clarify the positioning of the present study.

Following the reviewer’s suggestion, we have expanded the Introduction by adding a brief review of recent developments in structural topology optimization. The recommended studies on the enhanced proportional topology optimization method, the parameterized level-set method, and the asymptotic concentration method have been incorporated into the revised manuscript. Their main characteristics and applications in structural topology optimization are briefly discussed, together with their relationship to and differences from the optimization framework adopted in the present study.

These revisions provide a broader overview of recent developments in structural topology optimization and further clarify the positioning and potential extension of the present study in the coordinated topology and size optimization of large-span Suspen-Dome structures. The corresponding references have also been added to the revised manuscript.

(Pages 2,lines 49-63; Pages 36, lines 957-965)

Q3. Most of the images in the manuscript have low resolution and remain blurry even when enlarged, which makes it difficult to see their content clearly. Especially the images in Figures 3, 6, 8, 10, 11, 12, 14, 15, 16, 17, 19, 20, 21, 23, 24, 25, 26, 27. If applicable, it is recommended to carefully make appropriate modifications to the similar issues together.

Author’s Response:

Thank you for this careful comment. In response, we re-examined all figures throughout the manuscript, with particular attention to Figs. 3, 6, 8, 10–12, 14–17, 19–21, and 23–27, as specifically identified by the reviewer. For figures that could be regenerated or re-exported, higher-resolution versions were produced, and the sizes of the text, axis labels, legends, and other annotations were appropriately increased to improve their readability both at the normal display size and when enlarged.

For some finite element contour plots, the figures contain relatively dense information, including the overall structural model, contour distributions, color scales, and numerical values. For these figures, the output resolution, figure dimensions, display range, and annotation sizes were readjusted as far as practicable without altering the original computational results or contour information, thereby improving their clarity and readability. In addition, the other figures in the manuscript were also carefully checked, and those with similar issues were revised accordingly. All revised figures have been updated in the manuscript.

(Pages 7,lines 221; Pages 12,lines 361; Pages 14,lines 409; Pages 15, lines 464; Pages 18,19,lines 567; Pages 24, lines 685-687; Pages 30,lines 777-780)

Q4. If possible, it is recommended to change the color of the images in Tables 4 and 6, so as to enhance content recognition and readability.

Author’s Response:

Thank you for this valuable suggestion. In response, the images in Tables 4 and 6 were carefully re-examined and revised. Each table contains three structural diagrams showing the complete structure, the upper reticulated shell, and the lower cable–strut system, respectively. For the separate diagrams of the upper reticulated shell and the lower cable–strut system, clearer and more distinguishable colors were adopted to improve the identification of different structural components and enhance the overall readability of the figures.

For the complete structural diagrams, we also tested the use of different colors to distinguish the upper reticulated shell from the lower cable–strut system, together with increased line widths. However, because the complete structures contain a large number of members and considerable overlap occurs between the upper and lower components in the projected view, the use of multiple colors and thicker lines introduced additional visual congestion and made the overall topology and member connectivity less clear than in the original monochromatic representation. Therefore, after comparison, the original monochromatic presentation was retained for the complete structural diagrams in Tables 4 and 6, while the separate diagrams of the upper reticulated shell and the lower cable–strut system were revised using clearer colors. The corresponding figures have been updated in the revised manuscript.

(Pages 21-22; Pages 27-28)

Q5. The PLOS Data policy requires authors to make all data underlying the findings described in their manuscript fully available without restriction, with rare exception (please refer to the Data Availability Statement in the manuscript PDF file). The data should be provided as part of the manuscript or its supporting information, or deposited to a public repository. Although some data are provided in the manuscript, it does not seem to meet the requirements of the PLOS Data policy. Therefore, it is suggested that the authors make appropriate modifications based on the specific circumstances.

Author’s Response:

Thank you for this valuable comment. We agree that the accessibility of the data associated with this study should be further improved in accordance with the PLOS Data Policy. In response, we organized the data used for the machine-learning component of the proposed method and added a CSV data file as Supporting Information to the revised manuscript.

The CSV file contains the dataset used to train the MLP-based candidate screening model, including the corresponding structural design parameters, statistical features of the member sections, and sample feasibility labels. This dataset was used for the training and validation of the MLP screening model adopted in the optimization process to reduce unnecessary finite element analyses. Providing this dataset enables readers to more clearly understand the source and input format of the MLP training data and facilitates further verification of the associated training and screening process.

The Data Availability Statement has also been revised accordingly to specify that the CSV dataset is provided as Supporting Information with the revised manuscript. These revisions further improve the transparency and reproducibility of the data associated with the MLP model training.

Q6. PLOS ONE does not copyedit accepted manuscripts, so the language in submitted articles must be clear, correct, and unambiguous. Any typographical or grammatical errors should be corrected at revision, so the specific errors are noted as follows.

(1) When abbreviations first appear, they should be accompanied by their corresponding full names. This is a writing principle. However, in the abstract (line 18) and main text (line 120), the abbreviation “MLP” violates this principle. Moreover, the abbreviation “GSI&PS-PGSA” (line 203) violates this principle. If applicable, it is recommended to carefully make appropriate modifications to the similar issues together.

(2) The citations in the text should be accurate. This is also a writing principle. However, both "Kaveh [8]" (line 42) and "Adam, by contrast, considered minimizing member stresses and maximizing overall structural stiffness [31]" (lines 95-96) violate this principle, as both References [8] and [31] have more than one contributor. If applicable, it is recommended to carefully make appropriate modifications to the similar issues together.

(3) The typographical errors in the text should be eliminated. This is also a writing principle. However, "Examples 1" (line 199) violates this principle, and its correct form should be 'Example 1'. If applicable, it is recommended to carefully make appropriate modifications to the similar issues together.

(4) All Figures should be mentioned in the main text. This is also a writing principle. However, Figure 1 was not mentioned in the main text, which violates this principle. If applicable, it is recommended to carefully make appropriate modifications to the similar issues together.

Author’s Response:

Thank you for these careful comments. In response, we systematically checked the entire manuscript with particular attention to language expression, abbreviation definitions, citation style, typographical consistency, and figure citations, and the relevant issues have been revised throughout.

Specifically, (1) the full terms have now been provided when abbreviations first appear in the abstract and main text, including MLP and GSI&PS-PGSA, and other abbreviations throughout the manuscript were also checked for consistent first-use definitions; (2) the author citation style in the main text was reviewed, and citations involving references with multiple authors were revised to the appropriate form, together with a check of other similar cases; (3) typographical and grammatical errors such as “Examples 1” were corrected, and the manuscript was further checked for similar language and formatting issues; and (4) the first citation of Fig. 1 was added to the main text, and all other figures were reviewed to ensure that each figure is appropriately referred to in the manuscript.

These revisions have been incorporated into the revised manuscript to further improve the accuracy of the language, the consistency of the formatting, and the overall readability of the paper.

(1): (Pages1 ,lines18,19-20, 43; Pages 4, lines 134; Pages 7, lines223-224)

(2): (Pages1 ,lines 37,41,42; Pages 2 ,lines 63,65; Pages 3, lines95,109)

(3): (Pages6 ,lines 220)

Q7. If possible, it is recommended to carefully check and revise the entire manuscript, strive to eliminate any ambiguities and unreadable points, and thus improve the presentation of the manuscript.

Author’s Response:

Thank you for this valuable suggestion. In response, we conducted a further systematic review and language revision of the entire manuscript, with particular attention to sentences that could be ambiguous, unclear, or difficult to understand. Relevant expressions were reorganized and revised where necessary. Several overly long sentences or sentences with unclear logical relationships were also divided or rewritten, and the terminology, abbreviations, symbols, and related expressions were further standardized throughout the manuscript. In addition, grammatical, spelling, and formatting issues were carefully rechecked and corrected.

These revisions have further improved the clarity, logical coherence, and readability of the manuscript, and the corresponding changes have been incorporated into the revised version.

Reviewer #2: The manuscript presents a surrogate-assisted multi-objective topology optimization framework for prefabricated Suspen-Dome structures by integrating a structural mapping method (SMM), an improved plant growth simulation algorithm (iPGSA), a neural-network-based screening strategy, and a partial fully stressed design concept. Although the topic is relevant to structural optimization, the manuscript suffers from substantial methodological and scientific deficiencies. The claimed novelty is not sufficiently demonstrated, and many important methodological details are missing or lack rigorous validation. Therefore, I do not recommend publication in its current form.

Major comments:

Q1. The claimed methodological novelty is weak. The work mainly integrates existing methods rather than proposing a fundamentally new optimization methodology.

Author’s Response:

Thank you for this valuable comment. We agree that the original manuscript did not sufficiently clarify the hierarchy and boundaries of the methodological contributions, which may have made the structural mapping method, iPGSA, the pointer adjustment method, and the auxiliary computational modules appear to be a simple combination of existing techniques. However, the present study does not merely place these existing methods side by side.

The structural mapping method and iPGSA constitute the basic solution framework of this study, while the random parallel search and optimal-value elimination mechanisms in iPGSA were developed in the authors’ previous work. The main methodological contribution of the present study lies in adapting the bidirectional evolutionary concept of BESO to the coupled discrete topology–size–prestress optimization problem of Suspen-Dome structures and transforming it into a pointer adjustment mechanism suitable for discrete structural parameters. Conventional BESO generally adds or removes finite elements according to element sensitivities. In contrast, the present method does not directly add or remove elements. Instead, the number of rings, number of radial divisions, arrangement intervals of the cable–strut system, and member section parameters are taken as the adjustment variables. The direction in which the design variables should increase or decrease in the next iteration is determined according to the variation relationship between structural mass and a comprehensive axial-stiffness index in adjacent iterations.

Therefore, the contribution of this study is not to re-propose SMM or iPGSA themselves, but to extend the existing optimization framework specifically for the discrete coupled design problem of Suspen-Dome structures. This extension enables the topology, geometry, and section parameters of the upper reticulated shell to be optimized simultaneously with the topology, size, and prestress parameters of the lower cable–strut system, rather than optimizing the two subsystems separately. In addition, the elastic-support–tension-compensation iterative method proposed in this study is used to improve the accuracy of cable prestress determination, while the MLP model serves as a candidate pre-screening tool to reduce unnecessary finite element analyses.

Following the reviewer’s comment, we revised the statements regarding novelty in the abstract, Introduction, and Conclusions. The revised manuscript no longer describes the entire SMM-iPGSA framework as a completely new fundamental optimization algorithm. Instead, the contribution of this study is more accurately positioned as a methodological extension for the coupled discrete topology–size–prestress optimization of Suspen-Dome structures, including the pointer adjustment mechanism and its new application to integrated collaborative optimization. We believe that the variable representation, directional guidance, and coupled solution mechanisms developed for this specific structural system constitute a problem-driven methodological innovation rather than a simple assembly of existing methods.

Q2. The mathematical formulation is incomplete and inconsistent. The multi-objective formulation is mathematically unclear and the definition of structural stiffness is questionable.

Author’s Response:

Thank you for this valuable comment. We agree that simply expressing the two objectives in the original manuscript as “minf1+maxf2” does not accurately represent a Pareto multi-objective optimization problem. In addition, directly referring to the summation of EA/L over all members as “structural stiffness” could lead readers to interpret it as a rigorous measure of the overall structural stiffness derived from the global stiffness matrix or the load–displacement relationship. Therefore, the original terminology was not sufficiently precise.

In response, the mathematical formulations in Sections 3.1.1 and 3.1.4 have been reorganized in the revised manuscript. First, structural mass and the aggregate axial stiffness index are converted into two dimensionless minimization objectives as follows:

minF(x)=[\begin{matrix}f_1(x)\\f_2(x)\\\end{matrix}]=[M(x)M0K0K(x)]

where

M\left(x\right)=\sum_{e}^{N_e}{\rho\bullet A(x)\bullet L(x)}

and

K\left(x\right)=\sum_{e}^{N_e}\frac{E\bullet A(x)}{L(x)}

The term “structural stiffness” has been consistently replaced by “aggregate axial stiffness index” throughout the revised manuscript. This index is no longer interpreted as the rigorous overall stiffness of the structure, but is used only to compare the overall level of member axial stiffness among different candidate designs. The actual global deformation and stability performance of the structure are still constrained and verified through the maximum vertical displacement, member strength, slenderness ratio, compression–bending stability, eigenvalue buckling factor, and nonlinear buckling factor.

Second, the design variables have been reorganized into topological variables, geometric variables, section variables, and prestress and form-finding variables. The corresponding upper and lower bounds, together with the treatment of discrete integer variables, have also been clarified. In addition, the mathematical direction of the stability constraints has been corrected, such that the calculated buckling factors are required to be no less than their allowable values.

Furthermore, the Pareto dominance relationship and the updating rule for the non-dominated solution set have been added to the algorithmic procedure. Therefore, the revised objective functions, design variables, constraint conditions, and Pareto-based screening process are now mathematically consistent throughout the optimization formulation.

(Pages 9,lines281-289; Pages 10-11,lines 330-333)

Q3. The optimization algorithm lacks sufficient implementation details for reproducibility, including constraint handling, Pareto strategy, and algorithm parameters.

Author’s Response:

Thank you for this valuable comment. Although the original manuscript described the processes of candidate-solution generation, finite element analysis, Pareto screening, and iterative updating through the optimization flowchart and detailed algorithmic steps, some parameter definitions, candidate-retention rules, and termination criteria were not sufficiently specified, which could indeed affect the reproducibility of the method. The authors’ previous work had already presented the basic procedures for candidate generation, removal of duplicate solutions, finite element constraint checking, calculation of morphactin concentration, and loop termination. However, the original manuscript did not fully specify the implementation parameters of these procedures in the present multi-objective optimization problem.

To address this issue, we supplemented and standardized the existing flowchart and algorithmic steps. The main revisions are as follows:

The maximum capacity of the candidate pool used for selecting the next growth point in each iteration was specified as k=50. When fewer than 50 valid candidates are available, all candidates are retained. When the number exceeds 50, the candidates are ranked according to their morphactin concentrations, and the top 95% are retained.

The MLP classification threshold was specified as 0.2. When the predicted feasibility probability is not lower than 0.2, the candidate solution proceeds to ANSYS finite element verification; otherwise, it is eliminated before finite element analysis. The final feasibility of all candidate solutions is still determined by the ANSYS results together with the structural constraints.

The Pareto-set updating rule was added. After each iteration, the newly generated feasible non-dominated solutions are merged with the existing Pareto solution set, followed by a new non-dominated sorting procedure. No fixed maximum capacity is imposed on the Pareto solution set in this study. The parameter k=50 is used only to limit the size of the candidate pool for selecting the next growth point and does not limit the number of Pareto solutions.

The termination criteria were clarified. The maximum number of iterations is 300 for the inner size and prestress optimization and 10 for the outer topology optimization. The corresponding loop is terminated when the prescribed maximum number of iterations is reached or when no valid candidate solution exists in the current cycle. Objective-function stability is not adopted as an additional termination criterion in this study.

The constraint-handling procedure was further clarified. The MLP is used only for preliminary screening. Candidate solutions that pass the MLP screening must still be analyzed using ANSYS and are allowed to enter the Pareto non-dominated analysis only when they simultaneously satisfy the displacement, strength, slenderness, local stability, overall stability, and cable-tension-state constraints.

Instead of adding a new flowchart or separate algorithm box, these parameters and rules were incorporated into the existing flowchart and algorithmic steps, and the descriptions in the figures, main text, and actual computational procedure were made consistent. Through these revisions, the procedures for candidate generation, screening, constraint handling, Pareto-set updating, and termination are now more clearly defined, thereby improving the clarity and reproducibility of the proposed method.

(Pages 18 , lines 552-567; )

Q4. Validation of the improved PGSA is insufficient. Only limited benchmark tests are provided without statistical analysis.

Author’s Response:

Thank you for this valuable comment. The two main improvement mechanisms of iPGSA and their benchmark validation have been described in detail in the authors’ previous study. In that study, unimodal and multimodal benchmark functions, together with a K6 reticulated-shell optimization example, were used to evaluate iPGSA, and the results demonstrated improvements in search efficiency and optimization performance compared with the conventional PGSA, GA, ANGA, and other methods.

To avoid substantial duplication with the previous study, the revised manuscript does not reproduce all of these benchmark examples. Instead, the relevant study has been cited and its main validation results have been briefly summarized. In addition, following the reviewer’s suggestion, multiple independent runs were conducted for the two benchmark functions considered in the present manuscript. Statistical indicators, including the mean best objective value, standard deviation, success rate, and average number of iterations, were added to evaluate the stability and repeatability of the algorithmic results. The corresponding statistical results have been added to Table 1.

(Pages 7 ,lines 237-240)

Q5. No comparison is made with state-of-the-art topology optimization approaches such as SIMP, BESO, level-set, or gradient-based methods.

Author’s Response:

Thank you for this valuable suggestion. In response to this comment to include recent advanced topology optimization methods, the Introduction has been expanded to include relevant studies on the enhanced proportional topology optimization method, the parameterized level-set method, and the asymptotic concentration–isogeometric analysis method, thereby providing a more comprehensive review of current topology optimization approaches.

It should be noted that the SIMP method, conventional BESO, level-set methods, and most gradient-based approaches mentioned by the reviewer are primarily developed for continuum topology optimization. Their basic design variables are generally material densities or boundary-description parameters defined within a fixed design domain. In contrast, the Suspen-Dome considered in this study is a discrete spatial member system with explicit connectivity relationships. Its design variables include the number of rings, number of radial divisions, arrangement intervals of the cable–strut system, discrete section indices, and cable prestress. In addition, the optimization must satisfy engineering constraints involving member strength, slenderness ratio, local stability, overall stability, and the tension-only behavior of cables.

Therefore, the two classes of methods differ fundamentally in terms of topology representation, design variables, and constraint formulation. Applying SIMP or level-set methods directly to the present problem would require the discrete member system to be reformulated as a continuum material design domain, or an additional mapping relationship to be established between member existence and material density. Such a reformulation would change the parameterized Suspen-Dome optimization problem considered in this study and would therefore make a fair quantitative comparison under identical design variables and constraints difficult.

For this reason, continuum topology optimization methods were not directly adopted for numerical comparison in the present study. Instead, GA, PSO, ANGA, and related heuristic optimization methods, which are more suitable for discrete structural variables and nonlinear engineering constraints, were selected as comparison algorithms. The revised manuscript further clarifies the distinction between continuum topology optimization and the discrete member-system topology optimization considered herein, together with the rationale for selecting the comparison methods.

In addition, the pointer-based method developed in this study based on the BESO concept does not directly employ the conventional BESO procedure of adding and removing finite elements. Rather, it draws on the bidirectional evolutionary concept of BESO to provide directional guidance for the discrete topological and stiffness-related parameters of the Suspen-Dome. This distinction has also been further clarified in the revised manuscript.

(Pages 2,lines 49-63; Pages 36, lines 957-965)

Q6. The surrogate MLP model is insufficiently validated. Dataset generation, sample size, cross-validation, and robustness are not adequately discussed.

Author’s Response:

Thank you for this valuable comment. We agree that, although the original manuscript presented the loss curve of the MLP model together with Accuracy, Precision, Recall, and F1-score, the description of the dataset generation procedure, sample size, and cross-validation was not sufficiently detailed.

In response, Section 3.2.5 has been expanded to provide additional information on the dataset generation procedure, the total number of samples, the numbers of feasible and infeasible samples, and the criteria used to assign the corresponding feasibility labels. The proportions used to divide the dataset into training, validation, and test sets, together with the main training parameters of the MLP model, have also been clarified.

In addition, five-fold stratified cross-validation was performed, and the mean and standard deviation of Accuracy, Precision, Recall, and F1-score were reported to evaluate the stability of the model under different data partitions. The cross-validation results are generally consistent with those obtained from the independent test set, indicating that the MLP screening model provides relatively stable classification performance.

It should be emphasized that the MLP model is used only as an auxiliary classifier for candidate pre-screening, with the purpose of eliminating clearly infeasible structural candidates before finite element analysis. All retained candidates and final optimization results are still verified using ANSYS. In addition, the statement in the original manuscript that “the number of ANSYS calls was reduced by 25.6%” has been corrected to “the average computational time was reduced by approximately 25.6%,” so that the description is consistent with the actual computational results.

(Pages 15, lines 448-460,465-469; Pages 16, lines 471-472)

Q7. The proposed PFSD concept is heuristic and lacks theoretical justification.

Author’s Response:

Thank you for this valuable comment. We agree that the original manuscript did not sufficiently explain the rationale behind the proposed PFSD concept. It should be clarified that PFSD is not intended as a new structural theory, but rather as a design strategy developed according to the different mechanical roles of the upper reticulated shell and the lower cable-strut system in a Suspen-Dome.

In the revised manuscript, additional explanations have been added in Section 3.2.4 to clarify its mechanical basis. Specifically, the upper reticulated shell is regarded as the primary load-carrying subsystem that provides global stiffness and stability reserve, and therefore should retain an appropriate stress margin rather than strictly satisfying the full-stress criterion. By contrast, the lower cable system mainly resists axial tension without buckling and is responsible for prestress regulation, making it more suitable for a near full-stress design. Therefore, PFSD represents a mechanics-based design strategy for balancing structural safety reserve and material utilization, instead of a newly established structural theory.

(Pages 14-15, lines 417-436)

Q8. The optimization examples are limited, with no sensitivity or robustness studies.

Author’s Response:

Thank you for this valuable suggestion. The original manuscript employed two optimization examples with different spans and topological configurations, namely a 100 m-span Kiewitt–Lamella Suspen-Dome and a 110 m-span rib-ring Suspen-Dome, to provide an initial assessment of the applicability of the proposed method to different Suspen-Dome configurations. However, we agree that the original manuscript did not specifically investigate the effects of load variation or the stochastic nature of the optimization algorithm.

In response, a new Section 6, entitled “Sensitivity and Robustness Study,” has been added to the revised manuscript, using the 100 m-span Kiewitt–Lamella Suspen-Dome presented in Section 4 as the representative example.

For the sensitivity analysis, complete optimization procedures were independently performed under load levels of 1.0, 1.2, and 1.4 times the baseline load, while the design-variable ranges, algorithmic parameters, constraint conditions, and termination criteria were kept unchanged. The optimized structural masses under the three load levels were 271.56 t, 299.77 t, and 301.10 t, respectively. Compared with the baseline case, the structural masses under the latter two load levels increased by 10.39% and 10.88%, respectively. The corresponding maximum vertical displacements were 20.90 mm, 45.56 mm, and 34.47 mm. These results indicate that the optimized structural mass is relatively less sensitive to the investigated load variation, whereas structural deformation and the resulting topology and member configuration are more sensitive to the load level. All optimized solutions obtained under the three load cases satisfied the relevant structural constraints, indicating that the proposed method can adapt the topology, member sections, and prestress parameters to different load levels and obtain feasible designs.

For the robustness analysis, the optimization was independently repeated using three different random seeds while maintaining identical initial conditions, design-variable ranges, and algorithmic parameters. All three runs resulted in the same overall topological parameters, namely Kn=11 and Nx=12, with cable–strut arrangement intervals of 1 and 3. The mean, standard deviation, and coefficient of variation of the structural mass were 277.27 t, 7.22 t, and 2.60%, respectively. For the maximum vertical displacement, the corresponding values were 25.32 mm, 3.98 mm, and 15.72%, respectively. These results show good consistency in structural mass and overall topology among different stochastic search paths, although some variations remain in the local section configurations and deformation responses. Such variations reflect the trade-off between structural weight and deformation performance inherent in the multi-objective optimization problem.

Through these revisions, the manuscript now includes both a sensitivity analysis of the optimization results under different load levels and a robustness analysis based on independent runs with different random seeds.

(Pages 32-33 ,lines 812-866)

Q9. Engineering conclusions are largely qualitative and not fully supported by quantitative evidence.

Author’s Response:

Thank you for this valuable comment. We agree that some statements in the conclusions of the original manuscript relied mainly on qualitative descriptions such as “significantly reduced,” “small displacement,” and “good stability,” without sufficiently using the quantitative results of structural mass, displacement, and buckling analyses already presented in the main text.

In response, quantitative results for the two optimization examples have been added after Conclusion (5) in the revised manuscript. For the 100 m-span Kiewitt–Lamella Suspen-Dome, the structural mass was reduced from 1380.00 t to 271.56 t, corresponding to a reduction of 80.32%. The maximum vertical displacement was 20.90 mm, while the nonlinear buckling factor and eigenvalue buckling factor were 3.32 and 8.15, respectively. For the 110 m-span rib-ring Suspen-Dome, the structural mass was reduced from 336.00 t to 176.20 t, corresponding to a reduction of 47.56%. The maximum vertical displacement was 13.24 mm, and the nonlinear buckling factor and eigenvalue buckling factor were 3.14 and 6.68, respectively.

Accordingly, the original qualitative conclusion has been revised to state that the proposed method can substantially reduce structural mass while maintaining relatively small vertical deformation and an appropriate overall stability reserve. The corresponding conclusions are now supported by specific quantitative indicators, including the structural mass reduction ratio, maximum vertical displacement, and buckling factors.

In addition, stronger statements such as “can be directly applied to engineering practice” have been moderated and replaced by “can provide quantitative references for the preliminary design and parameter selection of large-span Suspen-Dome structures.” This revision ensures that the engineering implications of the conclusions remain consistent with the scope of evidence supported by the numerical analyses conducted in this study.

(Pages 35, lines 921-930; )

Q10. The manuscript requires substantial improvement in language, equation presentation, and organization.

Author’s Response:

Thank you for this valuable suggestion. In response, we conducted a further systematic review and language revision of the entire manuscript, with particular attention to sentences that could be ambiguous, unclear, or difficult to understand. Relevant expressions were reorganized and revised where necessary. Several overly long sentences or sentences with unclear logical relationships were also divided or rewritten, and the terminology, abbreviations, symbols, and related expressions were further standardized throughout the manuscript. In addition, grammatical, spelling, and formatting issues were carefully rechecked and corrected.

These revisions have further improved the clarity, logical coherence, and readability of the manuscript, and the corresponding changes have been incorporated into the revised version.

Reviewer #3: This manuscript presents a multiobjective optimization algorithm and a shift from the common separated design approach for Suspen-Dome structures toward a holistic, integrated optimization that simultaneously addresses size, geometry, and topology of the upper shell along with the topology and prestress of the lower cable-strut system. Although the manuscript is well written, there are some ambiguities in it, some of which are mentioned:

•Q1: There are some computational ambiguities in this manuscript. For example, on page 4, the creation of the connectivity matrix requires an example.

Author’s Response:

Thank you for this careful comment. We agree that the original manuscript provided the definition and construction procedure of the connectivity matrix without a specific example, which could make the formulation difficult to follow. In response, a simple illustrative example has been added to the corresponding part of the revised manuscript. Using a local structural system with clearly defined node numbers and member connectivity, the example demonstrates how the connectivity matrix is constructed from the end-node information of each member. This addition clarifies the correspondence among node numbering, member connectivity, and the entries of the connectivity matrix, making the construction procedure more intuitive and easier to understand.

( Pages 5, lines 167-174)

• Q2:On page 5, can “mmm” be represented mathematically in R3 space, or can the authors come up with a better mathematical expression for the representation?

Author’s Response:

Thank you for this careful comment. We agree that the mathematical representation in this part of the original manuscript was not sufficiently precise. In response, we re-examined the mathematical formulation and revised the relevant expressions . The corresponding symbols and their meanings have also been further clarified to improve the rigor and readability of the formulation. The relevant revisions have been incorporated into the revised manuscript.

(Pages 6 ,lines205)

• Q3:The author should define all parameters used, for example the parameter β in equation 7.

Author’s Response:

Thank you for this careful comment. In response, the definition and meaning of the parameter β in Eq. (8)( after correct ) have been added at the corresponding location in the revised manuscript. In addition, all parameters and symbols used in the equations throughout the manuscript were further checked. Definitions have been added or clarified for parameters whose meanings were not sufficiently explained at their first occurrence in the original manuscript, thereby improving the completeness and consistency of the mathematical notation throughout the paper.

(Pages 10, lines 319, Table 3)

• Q4:The domain space for design or population variables, such as Kn, should be defined.

Author’s Response:

Thank you for this careful comment. We agree that, although design variables such as Kn and Nx were introduced in the original manuscript, their admissible ranges and the basis for determining these ranges were not sufficiently specified. In response, the revised manuscript now explicitly defines the domains of the main design variables, including the upper and lower bounds and integer requirements for the topological variables Kn and Nx , the arrangement intervals of the cable–strut system, and the other discrete design variables. This revision provides a clearer definition of the optimization search space.

It should be noted that the ranges of these topological variables are not selected arbitrarily. Taking the circumferential division number Nx as an example, its value is determined by considering the structural span, shell radius, and target grid size. In this study, the outer-ring member length and the radial grid dimension are controlled within comparable ranges, with the principal grid dimensions maintained at approximately 6–10 m. This criterion provides relatively uniform grid geometry while also considering overall structural stiffness, stability of compression members, and practical constructability. Based on this criterion, the initial circumferential division numbers were set to 26 and 40 for the 100 m-span Kiewitt–Lamella Suspen-Dome and the 110 m-span rib-ring Suspen-Dome, respectively. The corresponding variable ranges and their discrete characteristics have now been explicitly specified in the revised manuscript.

(Pages 19, lines 579-586)

• Q5:In Table 1. The population used or produced during the pressing process should be written for each algorithm.

Author’s Response:

Thank you for this careful comment. In response, Table 2(after correct) has been revised to include the population size adopted or generated by each comparison algorithm during the optimization process. This additional information further clarifies the parameter settings and computational scale of the different algorithms. The corresponding population-size information is now consistently reported in Table 2 of the revised manuscript, thereby improving the transparency and reproducibility of the algorithmic comparison.

(Pages 8, lines 254-255)

Attachment Submitted filename: ResponsetoReviewer.docx

# Peer review: Decision Letter 1

**DOI:** 10.1371/journal.pone.0361862.r003

**Authors:** Antonio Riveiro Rodríguez

31 Aug 2026

Dear Dr. Dai,

Thank you for submitting your manuscript to PLOS One. After careful consideration, we feel that it has merit but does not fully meet PLOS One’s publication criteria as it currently stands. Therefore, we invite you to submit a revised version of the manuscript that addresses the points raised during the review process.

Please submit your revised manuscript by Oct 30 2026 11:59PM. If you will need more time than this to complete your revisions, please reply to this message or contact the journal office at plosone@plos.org. When you're ready to submit your revision, log on to https://www.editorialmanager.com/pone/ and select the 'Submissions Needing Revision' folder to locate your manuscript file.

- A letter that responds to each point raised by the academic editor and reviewer(s). You should upload this letter as a separate file labeled 'Response to Reviewers'.
- A marked-up copy of your manuscript that highlights changes made to the original version. You should upload this as a separate file labeled 'Revised Manuscript with Track Changes'.
- An unmarked version of your revised paper without tracked changes. You should upload this as a separate file labeled 'Manuscript'.

If you would like to make changes to your financial disclosure, please include your updated statement in your cover letter. Guidelines for resubmitting your figure files are available below the reviewer comments at the end of this letter.

If applicable, we recommend that you deposit your laboratory protocols in protocols.io to enhance the reproducibility of your results. Protocols.io assigns your protocol its own identifier (DOI) so that it can be cited independently in the future. For instructions see: https://journals.plos.org/plosone/s/submission-guidelines#loc-laboratory-protocols. Additionally, PLOS One offers an option for publishing peer-reviewed Lab Protocol articles, which describe protocols hosted on protocols.io. Read more information on sharing protocols at https://plos.org/protocols?utm_medium=editorial-email&utm_source=authorletters&utm_campaign=protocols.

As the corresponding author, your ORCID iD is verified in the submission system and will appear in the published article. PLOS supports the use of ORCID, and we encourage all coauthors to register for an ORCID iD and use it as well. Please encourage your coauthors to verify their ORCID iD within the submission system before final acceptance, as unverified ORCID iDs will not appear in the published article. *Only*  the individual author can complete the verification step; PLOS staff *cannot*  verify ORCID iDs on behalf of authors.

We look forward to receiving your revised manuscript.

Kind regards,

Antonio Riveiro Rodríguez, PhD

Academic Editor

PLOS One

Journal Requirements:

If the reviewer comments include a recommendation to cite specific previously published works, please review and evaluate these publications to determine whether they are relevant and should be cited. There is no requirement to cite these works unless the editor has indicated otherwise.

[Note: HTML markup is below. Please do not edit.]

Reviewers' comments:

Reviewer's Responses to Questions

**Comments to the Author**

Reviewer #1: All comments have been addressed

Reviewer #2: (No Response)

Reviewer #3: All comments have been addressed

**********

2. Is the manuscript technically sound, and do the data support the conclusions?

Reviewer #1: Yes

Reviewer #2: (No Response)

Reviewer #3: Yes

**********

3. Has the statistical analysis been performed appropriately and rigorously?

Reviewer #1: Yes

Reviewer #2: (No Response)

Reviewer #3: Yes

**********

4. Have the authors made all data underlying the findings in their manuscript fully available?

The PLOS Data policy

Reviewer #1: Yes

Reviewer #2: (No Response)

Reviewer #3: Yes

**********

5. Is the manuscript presented in an intelligible fashion and written in standard English?

Reviewer #1: Yes

Reviewer #2: (No Response)

Reviewer #3: Yes

**********

Reviewer #1: (No Response)

Reviewer #2: Thank you for the authors' detailed responses and revisions. However, after carefully considering the revised manuscript, I am still not convinced that the major concerns raised in the previous review have been adequately resolved.

The central issue remains the limited methodological novelty and insufficient validation of the proposed framework. The authors now clarify that SMM and iPGSA are existing methods and that the main contribution is an extension based on a BESO-inspired pointer adjustment mechanism for the coupled discrete optimization of Suspen-Dome structures. However, the revised manuscript still does not provide sufficiently convincing evidence that this mechanism constitutes a substantial methodological advance beyond the integration and problem-specific adaptation of existing optimization techniques. The additional MLP screening and PFSD strategy also appear largely auxiliary or heuristic, and their generality and effectiveness are not sufficiently demonstrated.

The authors have added more implementation details, cross-validation, sensitivity analysis, and robustness tests. Nevertheless, these additions mainly improve the completeness of the presentation rather than fundamentally addressing the above scientific concern. In particular, the validation remains limited to a small number of structural examples, and there is still insufficient comparative evidence to demonstrate the superiority, general applicability, and robustness of the proposed methodology over appropriate alternative approaches.

Therefore, although the revised manuscript is clearer than the previous version, I consider that the fundamental concerns regarding originality, methodological significance, and rigorous validation remain unresolved. In my opinion, addressing these issues would require substantial further methodological development and validation rather than another round of editorial revision.

I therefore cannot recommend publication of the manuscript.

Reviewer #3: The authors responded appropriately to all ambiguities and comments. They implemented the suggestions. The ambiguities have been responded, and the manuscript can be accepted.

**********

what does this mean?). If published, this will include your full peer review and any attached files.

If you choose “no”, your identity will remain anonymous but your review may still be made public.

**Do you want your identity to be public for this peer review?**  For information about this choice, including consent withdrawal, please see our Privacy Policy

Reviewer #1: No

Reviewer #2: No

Reviewer #3: No

**********

[NOTE: If reviewer comments were submitted as an attachment file, they will be attached to this email and accessible via the submission site. Please log into your account, locate the manuscript record, and check for the action link "View Attachments". If this link does not appear, there are no attachment files.]

To ensure your figures meet our technical requirements, please review our figure guidelines: https://journals.plos.org/plosone/s/figures

You may also use PLOS’s free figure tool, NAAS, to help you prepare publication quality figures: https://journals.plos.org/plosone/s/figures#loc-tools-for-figure-preparation.

NAAS will assess whether your figures meet our technical requirements by comparing each figure against our figure specifications.

# Author comment: Author response to Decision Letter 2

**DOI:** 10.1371/journal.pone.0361862.r004

1 Sep 2026

Author’s Response:

Thank you for again raising the important concerns regarding the originality, methodological significance, and adequacy of validation of the manuscript. We understand that the central concern in this round is no longer simply whether the methodological procedure is sufficiently described, but whether the integrated optimization treatment adopted in this study produces a substantive effect beyond a straightforward combination or problem-specific adaptation of existing techniques, and whether the available numerical evidence adequately supports this methodological significance. In response, we have further revised both the positioning of the contribution and the validation presented in the manuscript.

First, regarding originality, we have further clarified that the present study does not re-propose the Structural Mapping Method (SMM) or iPGSA themselves as new methods, nor are the MLP screening model and PFSD strategy claimed as independent general-purpose optimization theories. The principal contribution of this study is the extension of the existing modeling and search procedures to the coupled discrete topology–size–prestress optimization problem of Suspen-Dome structures, in which the topology, geometry, and member sections of the upper reticulated shell are optimized together with the layout, member sizing, and cable prestress of the lower cable–strut system within the same design space and under the same structural constraints. The Introduction and Discussion have therefore been revised to more clearly define this problem-oriented methodological contribution and to avoid overstating the independent novelty of the auxiliary components.

Second, regarding methodological significance, we agreed that the original manuscript mainly explained the need for integrated optimization conceptually, without providing sufficiently direct comparative evidence. Therefore, a new Section 6.3, entitled “Comparison between Sequential and Integrated Optimization Strategies,” has been added using the 100 m-span Kiewitt–Lamella Suspen-Dome as a controlled example. In the sequential strategy, the upper reticulated shell is optimized first. During this stage, the lower cable–strut system is not treated as a set of optimization variables; however, its nodal connectivity, geometric parameters, and prestress equilibrium state are updated according to the current upper-shell configuration to maintain geometric compatibility and structural equilibrium. After the representative upper-shell solution is obtained, its topology, geometry, and member sections are fixed, and the lower cable–strut system is subsequently optimized. In contrast, the integrated strategy allows the design variables associated with both subsystems to be adjusted within the same optimization process. The two strategies use identical initial structural conditions, material properties, loading conditions, objective functions, structural constraints, design-variable bounds, finite-element models, and algorithmic parameters. Thus, the comparison primarily reflects the effect of sequential versus integrated treatment of the two mechanically coupled subsystems rather than differences in the underlying optimization algorithm or structural analysis model.

The newly added Table 12 shows that the two strategies lead to different structural configurations and performance characteristics. The structural mass obtained by the integrated strategy is 271.56 t, compared with 303.50 t for the sequential strategy, corresponding to a reduction of 10.52%. The maximum vertical displacement is reduced from 30.06 mm to 20.90 mm. Differences are also observed in the resulting topological parameters: Kn changes from 13 in the sequential strategy to 11 in the integrated strategy, while the cable–strut arrangement interval changes from 1,1 to 1,3. The nonlinear buckling factor of the integrated solution is slightly lower than that of the sequential solution (3.32 versus 3.79), but both solutions satisfy the prescribed stability requirements. These results demonstrate that sequential and integrated treatment of the upper and lower structural systems leads to different attainable design solutions under otherwise identical structural constraints. In particular, allowing the topology, size, and prestress-related variables of the two mechanically coupled subsystems to interact within the same search process results in a lighter structural solution with smaller vertical deformation. We believe that this controlled comparison provides more direct numerical evidence of the methodological effect of the integrated treatment than was available in the previous version of the manuscript.

Third, regarding validation, the validation strategy has been reorganized and strengthened. The revised manuscript now includes: (1) algorithm-level validation of iPGSA using benchmark functions and a reticulated-shell optimization example with comparison algorithms; (2) complete optimization of two Suspen-Dome configurations, namely the 100 m-span Kiewitt–Lamella Suspen-Dome and the 110 m-span rib-ring Suspen-Dome; (3) independent testing and five-fold cross-validation of the MLP screening model; (4) sensitivity analysis under different load levels; (5) robustness analysis using different random seeds; and (6) the newly added controlled comparison between sequential and integrated optimization strategies. At the same time, statements regarding general applicability and superiority have been further moderated. The present study does not claim universal superiority over all topology optimization methods or unrestricted applicability to arbitrary spatial structural systems; the conclusions are limited to the investigated Suspen-Dome configurations and parameter ranges.

Through these revisions, we have sought to more clearly distinguish the problem-oriented methodological extension developed in the present study from the existing underlying methods and to provide direct comparative evidence for the practical significance of the integrated optimization treatment. We sincerely appreciate the reviewer’s comment, which helped us further clarify the scope of the claimed originality and strengthen the corresponding comparative validation and scientific evidence.

(Pages 3-4, lines 126-155; Pages 33, lines 823; Pages34-35, lines 878-911; Pages 35-36, lines 915-923; Pages 36, lines944-950; Pages 37, lines971-976 )

Attachment Submitted filename: ResponsetoReviewesecond.docx

# Peer review: Decision Letter 2

**DOI:** 10.1371/journal.pone.0361862.r005

**Authors:** Antonio Riveiro Rodríguez

19 Oct 2026

Surrogate-Assisted Multi-objective Topology Optimization Design Method for Prefabricated Large-Span Suspen-Dome Based on Structural-Mapping-Method and Improved-PGSA

PONE-D-26-28981R2

Dear Dr. Dai,

We’re pleased to inform you that your manuscript has been judged scientifically suitable for publication and will be formally accepted for publication once it meets all outstanding technical requirements.

Within one week, you’ll receive an e-mail detailing the required amendments. When these have been addressed, you’ll receive a formal acceptance letter and your manuscript will be scheduled for publication.

An invoice will be generated when your article is formally accepted. Please note, if your institution has a publishing partnership with PLOS and your article meets the relevant criteria, all or part of your publication costs will be covered. Please make sure your user information is up-to-date by logging into Editorial Manager at Editorial Manager® and clicking the ‘Update My Information' link at the top of the page. For questions related to billing, please contact billing support.

If your institution or institutions have a press office, please notify them about your upcoming paper to help maximize its impact. If they’ll be preparing press materials, please inform our press team as soon as possible -- no later than 48 hours after receiving the formal acceptance. Your manuscript will remain under strict press embargo until 2 pm Eastern Time on the date of publication. For more information, please contact onepress@plos.org.

Kind regards,

Antonio Riveiro Rodríguez, PhD

Academic Editor

PLOS One

Additional Editor Comments:

Based on the reviewer comments, no further amendments are necessary.

Reviewers' comments:

Reviewer's Responses to Questions

**Comments to the Author**

Reviewer #1: All comments have been addressed

Reviewer #2: (No Response)

Reviewer #3: All comments have been addressed

**********

2. Is the manuscript technically sound, and do the data support the conclusions?

Reviewer #1: Yes

Reviewer #2: No

Reviewer #3: Yes

**********

3. Has the statistical analysis been performed appropriately and rigorously?

Reviewer #1: Yes

Reviewer #2: N/A

Reviewer #3: Yes

**********

4. Have the authors made all data underlying the findings in their manuscript fully available?

The PLOS Data policy

Reviewer #1: Yes

Reviewer #2: No

Reviewer #3: Yes

**********

5. Is the manuscript presented in an intelligible fashion and written in standard English?

Reviewer #1: Yes

Reviewer #2: No

Reviewer #3: Yes

**********

Reviewer #1: The authors have carefully revised the manuscript according to the comments provided. Both the technical content and the presentation of the manuscript have been greatly improved. Therefore, this manuscript is now acceptable for publication.

Reviewer #2: (No Response)

Reviewer #3: The comments have been addressed, and the manuscript can be accepted. All required questions have been answered and that all responses meet formatting specifications

**********

what does this mean?). If published, this will include your full peer review and any attached files.

If you choose “no”, your identity will remain anonymous but your review may still be made public.

**Do you want your identity to be public for this peer review?**  For information about this choice, including consent withdrawal, please see our Privacy Policy

Reviewer #1: No

Reviewer #2: No

Reviewer #3: No

**********

# Editor report: Acceptance letter

**DOI:** 10.1371/journal.pone.0361862.r006

**Authors:** Antonio Riveiro Rodríguez

PONE-D-26-28981R2

PLOS One

Dear Dr. Dai,

I'm pleased to inform you that your manuscript has been deemed suitable for publication in PLOS One. Congratulations! Your manuscript is now being handed over to our production team.

At this stage, our production department will prepare your paper for publication. This includes ensuring the following:

* All references, tables, and figures are properly cited

* All relevant supporting information is included in the manuscript submission,

* There are no issues that prevent the paper from being properly typeset

You will receive further instructions from the production team, including instructions on how to review your proof when it is ready. Please keep in mind that we are working through a large volume of accepted articles, so please give us a few days to review your paper and let you know the next and final steps.

Lastly, if your institution or institutions have a press office, please let them know about your upcoming paper now to help maximize its impact. If they'll be preparing press materials, please inform our press team within the next 48 hours. Your manuscript will remain under strict press embargo until 2 pm Eastern Time on the date of publication. For more information, please contact onepress@plos.org.

You will receive an invoice from PLOS for your publication fee after your manuscript has reached the completed accept phase. If you receive an email requesting payment before acceptance or for any other service, this may be a phishing scheme. Learn how to identify phishing emails and protect your accounts at https://explore.plos.org/phishing.

If we can help with anything else, please email us at customercare@plos.org.

Thank you for submitting your work to PLOS One and supporting open access.

Kind regards,

PLOS One Editorial Office Staff

on behalf of

Dr. Antonio Riveiro Rodríguez

Academic Editor

PLOS One
